# The effectiveness and safety of acupuncture for knee osteoarthritis: An overview of systematic reviews

**DOI:** 10.1097/MD.0000000000016301

**Published:** 2019-07-12

**Authors:** Juan Li, Yu-Xi Li, Liao-Jun Luo, Jing Ye, Dong-Ling Zhong, Qi-Wei Xiao, Hui Zheng, Chun-Mei Geng, Rong-Jiang Jin, Fan-Rong Liang

**Affiliations:** a School of Health Preservation and Rehabilitation, Chengdu University of Traditional Chinese Medicine; b School of Acupuncture-Moxibustion and Tuina, The Third Affiliated Hospital, Chengdu University of Traditional Chinese Medicine, Chengdu, Sichuan; c Rehabilitation Department, Kunming Municipal Hospital of Traditional Chinese Medicine, Yunnan, China.

**Keywords:** acupuncture, knee osteoarthritis, meta-analysis, overview, systematic review

## Abstract

**Background:**

Knee osteoarthritis (KOA), known as severe degenerative arthritis, commonly occurs in middle-aged and elderly people all over the world. Acupuncture as traditional oriental intervention is getting widely used and several systematic reviews (SRs) have reported the effectiveness of acupuncture on pain relief and functional recovery in patients with KOA.

**Objective:**

Conducting an overview of SRs to provide more reliable evidence-based medical references for clinical practitioners and researchers of the effectiveness and safety of acupuncture for KOA.

**Data sources:**

EMBASE, Medline, Web of science, the Cochrane library, China National Knowledge Infrastructure, the Chinese Science and Technology Periodical Database, China Biology Medicine, Wan Fang Digital Journals, and PROSPERO databases from inception to December 2018, magazines, websites, and unpublished sources.

**Selection criteria:**

Potential SRs were independently selected by 2 reviewers following a predetermined protocol.

**Data extraction:**

Data information of included SRs were independently extracted by 2 reviewers following a predetermined standardized data extraction form.

**Review appraisal:**

The risk of bias and reporting quality of included SRs were evaluated by the Risk of Bias in Systematic reviews (ROBIS) tool and the Preferred Reporting Item for Systematic Review and Meta-analysis (PRISMA) statement. The quality of evidence of outcomes was evaluated by the Grading of Recommendations Assessment, Development and Evaluation (GRADE).

**Results:**

A total of 12 SRs were included. All the SRs were published in recent 12 years, ranging from 2006 to 2017. According to ROBIS, 4 SRs were in low risk in domain 1 and 7 in domain 3 of phase 2, and 2 SRs were low risk in phase 3. Among 27 items of PRISMA, 19 items were reported over 70% of compliance. Using GRADE assessment, of 34 outcomes, high quality of evidence was found in 5 outcomes, 17 outcomes were rated moderate quality, and 11 outcomes were low quality. According to high-quality outcomes, acupuncture had more total effective rate, short-term effective rate, and less adverse reactions than western medicine in treating KOA. In terms of Lequesne index and Lysholm knee score scale score, the effectiveness of electroacupuncture was better than that of western medicine.

**Limitations:**

There might be missing information. There may be duplicated clinical trials included by each SR that might have impact on the synthetic findings.

**Conclusions:**

According to the high-quality evidence, we concluded that acupuncture may have some advantages in treating KOA. However, there are some risk of bias and reporting deficiencies still needed to be improved.

## Abstract 2

Supplemental Digital Content is available in the text

## 1 Introduction

Knee osteoarthritis (KOA), known as severe degenerative arthritis, commonly occurs in middle-aged and elderly people all over the world.^[1]^ It causes pain and restricted movement that greatly reduces the quality of life of the patients.^[2]^ It was reported that Chinese people over the age of 60 have KOA symptoms hared a high incidence (19.4%),^[3]^ the prevalence of symptomatic KOA was higher in women (10.3%) compared with men (5.7%).^[4]^ With the aging and increasing obesity of the world's population, KOA ranked the 11th highest contributor to global disability and 38th highest in disability-adjusted life years.^[5]^ The current therapeutic program for KOA is drug interventions, such as analgesics, nonsteroidal anti-inflammatory drugs which mainly relieve symptoms and restore the function of knee joint.^[6,7]^ Nevertheless, many undesirable drug-related adverse events (AEs) including bleeding, perforation ulcers of stomach,^[8]^ and increased risk of cardiovascular disease^[9]^ limit the use of these drugs. Therefore, nonpharmacological treatment has become increasingly prevalent for both doctors and patients with KOA.^[10]^

Acupuncture as traditional oriental intervention^[11]^ is getting widely used around the world. It is known as an effective and safe therapy for pain relief, which is suitable for different types of musculoskeletal pain. Recently, several systematic reviews (SRs)^[12–14]^ have reported the efficacy of acupuncture on pain relief and functional recovery in patients with KOA. SR is considered to be one of the important sources of high-quality evidence. However, its quality is easily affected by many confounding factors. Without the strict quality assessment, SR may be misleading the decision makers when it is recommended as the best evidence to guide clinical practice. Hence, it is necessary for us to assess the quality of SRs.

Overview of SRs is a method of compiling evidence and synthesizing the results of multiple SRs. The more information collected, the better quality of evidence can be provided for clinical work. An overview of SRs on Traditional Chinese Medicine (TCM) for KOA^[15]^ has been published recently, which concluded that TCM generally appeared to be effective for the treatment of KOA. Nevertheless, the evidence for the effectiveness of acupuncture as the treatment for KOA has not been thoroughly evaluated yet. Therefore, we conducted this overview of acupuncture as intervention for KOA patients, critically appraised and synthesized the results from these SRs in order to provide more reliable evidence-based medical references for clinical practitioners and researchers.

## 2 Methods

### 2.1 Registration

The protocol of this overview has been registered with the international prospective register of SRs (PROSPERO, http://www.crd.york.ac.uk/PROSPERO, registration number: CRD42018082723). The overview of SRs was reported in accordance with the guidelines of the Preferred Reporting Item for Systematic Review and Meta-analysis (PRISMA) and a pilot version checklist with Preferred Reporting Items for overview of systematic reviews (PRIO-harms)^[17]^ to promote a more balanced reporting of benefits and harms.

### 2.2 Ethics

Ethics approval is not required in overview of SRs.

### 2.3 Inclusion criteria for this overview

The SRs of acupuncture for KOA met the inclusion criteria as following were included.

#### 2.3.1 Types of studies

SRs of randomized controlled trials (RCTs) or quasi-RCTs were included, in which acupuncture was utilized as the treatment for KOA.

#### 2.3.2 Types of participants

Participants who have been diagnosed as KOA in accordance with the diagnostic criteria of standard diagnostic criteria (the Chinese Medical Association criteria or the American College of Rheumatology criteria).^[16]^ There were no restrictions on gender, age, or race.

#### 2.3.3 Types of interventions

The studies which acupuncture (electroacupuncture, auricular acupuncture, warm-acupuncture, dry needling, etc.) used as intervention to treat KOA were included.

#### 2.3.4 Types of comparators

The studies in which sham acupuncture, placebo, waiting list, medicine, or other type of nonpharmaceutical therapy were utilized as control.

#### 2.3.5 Types of outcome measures

The outcomes were recommended in the Osteoarthritis Research Society International (OARSI) Clinical Trials Recommendations,^[17]^ including benefit outcomes, patient-reported outcomes, objective outcomes, structural outcomes, biochemical biomarkers, and adverse effects.

### 2.4 Exclusion criteria for this overview

SRs which included non-RCTs (cohort study, observational study, etc.); SRs which cannot be obtained after contacting the original author; SRs which duplicate published; SRs which did not do the meta-analysis. The SRs of acupuncture for KOA met the exclusion criteria mentioned above were excluded.

### 2.5 Search methods for identification of studies

#### 2.5.1 Database and search

Four electronic international (Web of Science, The Cochrane Library, Medline, and EMBASE) and 4 Chinese electronic databases (China National Knowledge Infrastructure, the Chinese Science and Technology Periodical Database, China Biology Medicine disc, and Wan Fang Digital Journals) from their inception until December 2018 were searched for potential SRs. PROSPERO database and Cochrane Library were also searched. Magazines and websites relevant with acupuncture for KOA were searched to avoid missing eligible SRs. The concrete search strategies were presented in Appendix 1. Experts in the field were consulted for unpublished SRs. There was no restriction on language.

#### 2.5.2 Selection of SRs

All the retrieved studies were imported into Endnote (X8) and the duplicated articles were filtered. Two reviewers (L.J.L. and Y.X.L.) independently screened titles and abstracts to determine eligibility according to the inclusive and exclusive criteria. Two reviewers (L.J.L. and Y.X.L.) downloaded the full text of all possibly relevant studies for further assessment independently then cross-checked. The references of retrieved articles were reviewed for candidates. If necessary, discrepancies were resolved by consensus between 2 reviewers. A third reviewer (J.L.) was invited for consensus adjudication if discrepancy were not resolved. We compiled a list (Appendix 2) of all the excluded studies with reasons.

#### 2.5.3 Data extraction

A standardized data extraction form was designed in advance. After identifying all the eligible SRs, 2 authors (D.L.Z. and J.Y.) independently extracted data according to data extraction form and then cross-checked. Information such as year of publication, number of patients enrolled, participant characteristics, features of interventions in treatment and control groups, types of outcome assessment, methodological quality of primary studies, data analysis approaches, sources of funding, and AEs were extracted. When the data was incomplete, the reviewers tracked back to the primary studies of included SRs.

#### 2.5.4 Assessment of risk of bias and reporting quality

Two authors (D.L.Z. and J.Y.) evaluated the risk of bias and reporting quality of the included SRs independently by using ROBIS and the PRISMA. Consensus was reached by discussion between 2 reviewers or an independent decision obtained from the expert (J.L.), if necessary.

(1) ROBIS tool^[18]^: The ROBIS is a tool to assess the risk of bias of SRs which comprised of phase 2 (4 domains) and phase 3. Four domains in phase 2 are “study eligibility criteria,” “identification and selection of studies,” “data collection and study appraisal,” and “synthesis and findings.” The results of each domain and phase 3 were rated as “high risk,” “low risk,” or “unclear risk.”
(2) PRISMA statement^[19]^: The PRISMA statement for reporting quality consists of a 27-item checklist and a 4-phase flow diagram. The checklist includes items deemed essential for transparent reporting of a SR. Each item of the PRISMA form was graded as “yes,” “incomplete,” or “no” and respectively scored as 1, 0.5, or 0 points for statistical analysis purposes. The sum of all items scored for each questionnaire was divided by its maximum possible score to assess study quality as a percentage. Study quality related to its PRISMA score percentage was rated as: very poor (<30%), poor (30–50%), fair (50–70%), good (70–90%), and excellent (>90%).

#### 2.5.5 Assessment of quality of evidence

The quality of evidence of the included SRs was evaluated by the Grading of Recommendations Assessment, Development and Evaluation (GRADE) approach. This tool was designed to evaluate the quality of evidence for each outcome measure across studies. Two authors (D.L.Z. and J.L.) who were trained in the GRADE center in China (Lanzhou) independently assessed the evidence of the outcomes, and the downgraded or upgraded factors affecting the quality of evidence should be described in detail to guarantee the reliability and transparency of results. The factors were related to the risk of bias, inconsistency, indirectness, imprecision, and publication bias. The overall quality of evidence was judged as “high,” “moderate,” “low,” or “very low.”

## 3 Results

### 3.1 Characteristics of included SRs

**Figure 1:**
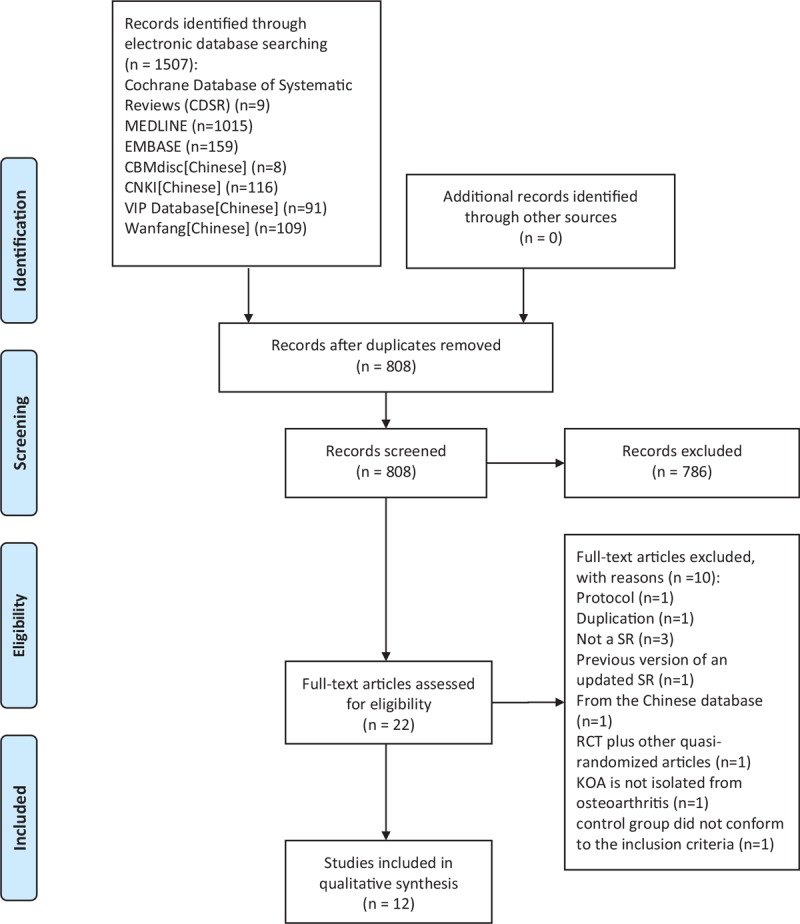
Flowchart of the systematic reviews selection process. CNKI = China National Knowledge Infrastructure, VIP = the Chinese Science and Technology Periodical Database, CBM = China Biology Medicine disc, KOA = knee osteoarthritis, RCT = randomized controlled trial; SR = systematic review.

The main characteristics (sample size, characteristics of patients, interventions, comparator outcomes, etc.) of the 12 included SRs^[13,20–30]^ were summarized in Table 1. Details of the literature search and SR selection can be found in Figure 1. All the SRs were published in recent 12 years, ranging from 2006 to 2017. All SRs contained RCTs, while 3 SRs^[28–30]^ also included quasi-RCT. Three SRs^[24,26,30]^ specified the diagnostic criteria of the included studies, while the others were unclear. The intervention was mainly acupuncture and main comparators were sham acupuncture and western medicine. For outcomes, most of the SRs (75%) considered the Western Ontario and McMaster Universities Arthritis Index, and 3 SRs^[20,25,27]^ reported visual analogue score. Only 3 SRs^[28–30]^ assessed AEs. Five SRs used the Jadad score for assessment of methodological quality and 6 used the Cochrane Collaboration's tool, 1 SR^[23]^ did not mention the appropriate method. All the 12 SRs performed meta-analysis, with 4 SRs^[23,27,28,30]^ completed subgroup analysis, and 3^[21,23,28]^ conducted sensitivity analysis. Safety associated with acupuncture was reported in 6 SRs.

**Table 1:**
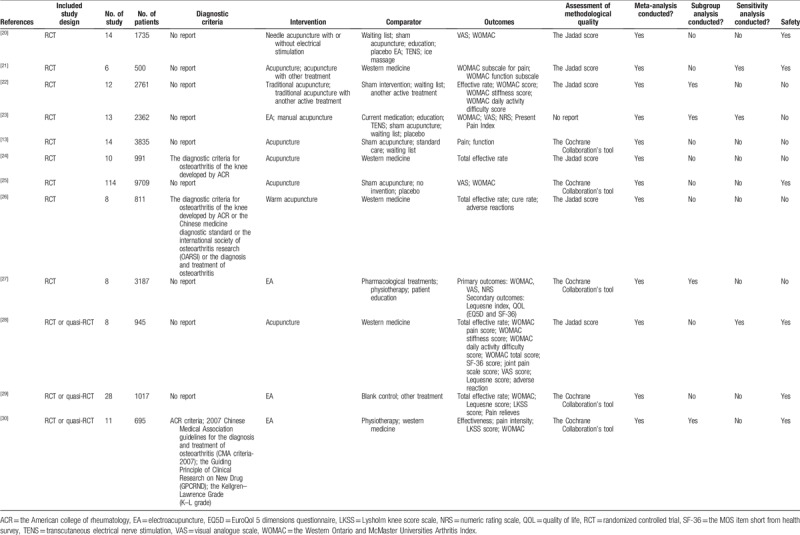
Characteristics of included systematic reviews.

### 3.2 Risk of bias of in the included SRs assessed by ROBIS

The risk of bias of the included SRs was determined using ROBIS. Table 2 presents the results of assessment. The domain 1 aimed to assess whether primary study eligibility criteria were prespecified, clear, and appropriate to the review question. Four out of 11 SRs^[20,22,23,27]^ were rated low risk and 2^[26,30]^ were unclear risk. Domain 2 focused on the identification and selection of studies in the SRs. All the articles were rated high risk in this domain. Incomplete search and incomplete search strategies were the main reasons for the downgrades. Domain 3 assessed the risk of bias through data collection and processes of appraise studies. Seven SRs were of low risk while 4 SRs^[20,23,24,26]^ were graded as high risk. Domain 4 aimed to assess whether the data was combined from the included primary studies. Only 3 SRs^[22,23,26]^ rated low risk of bias. Phase 3 focused on judging risk of bias of the SRs, 9 SRs were rated high risk and 2^[22,27]^ were low.

**Table 2:**
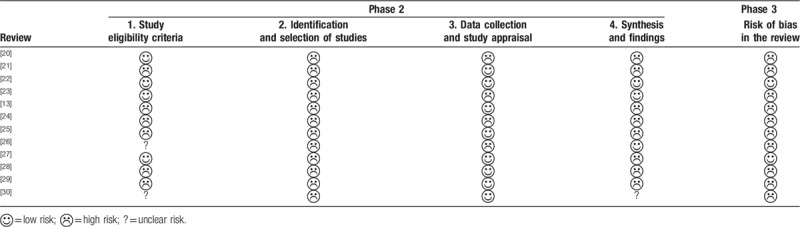
Risk of bias of the included systematic reviews assessed by risk of bias in systematic reviews.

### 3.3 Reporting quality of in the included SRs assessed by PRISMA

The reporting quality of the included SRs was accessed using PRISMA. Table 3 presents the results of assessment. Of the 27 items, 19 items were reported over 70% of compliance. The 12 items that were adequately reported with 100% of compliance were as followed: provide a structured summary (item 2); describe the rationale for the review in the context of what is already known (item 3); provide an explicit statement of questions (item 4); specify study characteristics and report characteristics (item 6); describe all information sources in the search (item 7); state the principal summary measures (item 13); describe the methods of handling data and combining results of studies (item 14); present the results of individual studies (item 20); present results of each meta-analysis done (item 21); summarize the main findings with relevance to key groups (item 24); discuss limitations at study and outcome level (item 25); and provide a general interpretation of the results in the context of other evidence (item 26). Three items with compliance lower than 40% were the main reporting limitations to be blamed: indicating if a protocol exists or is registered (item 5, 16.67%); present full electronic search strategy for at least 1 database (item 8, 33.33%); specify any assessment of risk of bias that may affect the cumulative evidence (item 15, 25%); and present results of any assessment of risk of bias across studies (item 22, 33.3%). On the whole, the reporting quality of 3 reviews were rated as “fair,” 8 were rated as “good,” and 1 was rated as “excellent.”

**Table 3:**
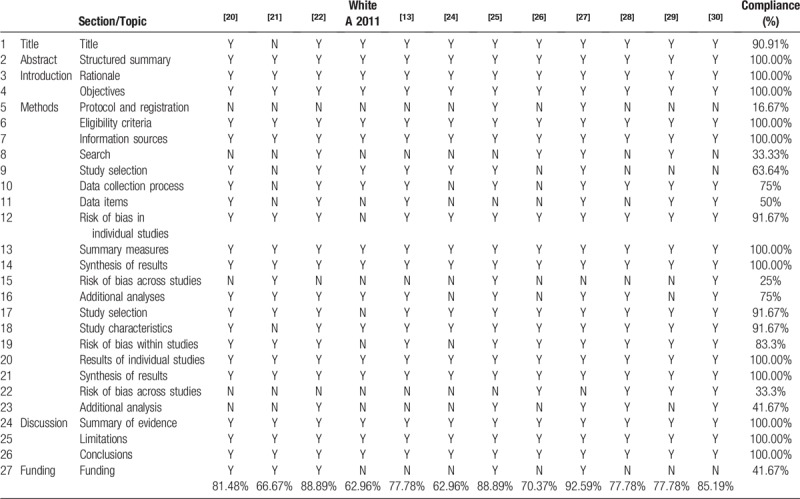
Reporting quality of the included systematic reviews assessed by preferred reporting items for systematic review and meta-analysis.

### 3.4 Quality of evidence in the included SRs assessed by GRADE

The quality of evidence for main outcomes in 12 included SRs is presented in Table 4. By using the GRADE approach, high quality of evidence was found in 5 outcomes of the included SRs, 17 outcomes were rated moderate quality, and 11 outcomes were low quality. The evidence was downgraded to either “moderate” or “low” quality because of the following limitations: the majority outcomes were downgraded by the small number of participants. The number of cases included in the studies did not reach the optimal information size. We subsequently downgraded the quality of evidence based on imprecision. For nearly half of the outcomes, owing to the high *I*^2^ values, and statistically significant heterogeneity of effect estimates could not provide a convincing explanation for differences in results between studies. Some of the outcomes had high probability of publication bias which could not be ruled out because of the incomprehensive literature search.

**Table 4:**
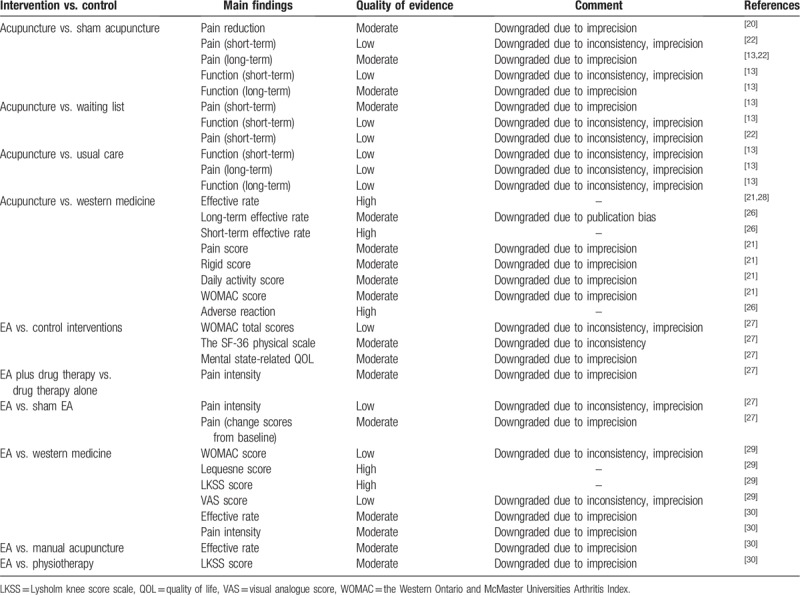
Quality of evidence in the included systematic reviews assessed by Grading of Recommendations Assessment, Development and Evaluation.

### 3.5 The effectiveness and safety of acupuncture for KOA

According to the high quality of evidence assessed by GRADE, 2 SRs^[28,29]^ suggested that patients with KOA who received acupuncture had better effects than their counterparts who were treated with western medicine measured with total effective rate (odds ratio = 4.08, 95% confidence interval [CI] [2.42, 6.88], *P* < .00001) and short-term effective rate^[26]^ (risk ratio [RR] = 2.35, 95% CI [1.59, 3.45], *P* < .0001). And 1 SR^[26]^ also reported less adverse reactions (RR = 0.20, 95% CI [0.05, 0.75], *P* = 0.02). One SR^[29]^ showed that electroacupuncture was better in improving knee function compared to western medicine measured with Lequesne index (mean diffrence [MD] = −1.79, 95% CI [−2.22, −1.37], *P* < .00001) and Lysholm knee score scale (LKSS) score (MD = 9.00, 95% CI [4.53, 13.47], *P* < .00001).

## 4 Discussion

### 4.1 Summary of main findings

This overview aimed to critically evaluate the evidence from SRs and provide a summary of effects on acupuncture in treatment of KOA. By using the ROBIS according to low risk of bias in ROBIS, 4 SRs were in low risk in domain 1, 7 in domain 3, and 2 in phase 3. By using PRISMA to assess SRs respectively, most of the SRs were regarded as relatively good reporting quality. The results of GRADE suggested that, acupuncture has more total effective rate, short-term effective rate, and less adverse reactions than western medicine as a treatment for KOA; the effectiveness of electroacupuncture is better than western medicine in terms of Lequesne index and LKSS score.

### 4.2 Implication for future study

By assessing the risk of bias and reporting quality of the included SRs respectively, we found that there were several common areas needed to be improved. Of the 11 SRs assessed, only 2 SRs managed to provide a documented protocol or registration. The registration item is one of the preferred reporting items in the PRISMA guidelines^[31]^ and critical in phase 2 checklist of ROBIS, which means it is important to register before conducting a SR. The registration helps promote transparency, minimize potential bias in the conduct and reporting of the review, reduce duplication of effort between groups, and keep SRs updated.^[31,32]^ To maintain a higher caliber evidence, the reviewers are strongly suggested to apply a registration in advance. A free and open database, the International Prospective Register of Systematic Reviews (PROSPERO, http://www.crd.york.ac.uk/prospero), has been advocated and recommended.^[33,34]^

Although all the SRs had searched at least 2 databases, only 1^[22]^ reported the full search strategies and supplemented by other relevant search of possible studies. Efficient literature searching and the application of formal rules of evidence in evaluating the clinical literature are the 2 key skills defining the practice of evidence-based medicine.^[35]^ Not only the bibliographic databases (e.g., EMBASE, Central, and Medline) should be searched, the published reviews, specialized registers, studies found by reviewing the reference list from searched articles and information from experts in the particular field of study are needed to be supplemented. Authors of SRs also should have searched trial registries, conference abstracts, dissertations, and unpublished reports on personal websites for grey literature in order to complete a comprehensive search.

The phase 3 is regarded as an important part in ROBIS checklist. However, only 2 SRs were rated as “low risk.” Authors of review should have a clear understanding and make a correct evaluation of their own studies, which makes studies more objective and gives convincing advice for further study. In consequence, it is crucial for authors of review to give appropriate and detailed explanation of the bias in SRs.

The risk of bias across studies is mainly caused by inadequate reporting or incomplete information, which may result in inaccuracy of a review. In our overview, only 2 SRs^[22,25]^ intended to explore the possible biased data and 3 SRs^[22,24,26]^ actually assessed the publication bias by evaluating a funnel plot. Researchers found that findings were more likely to be published if the results were positive,^[36]^ outcomes that are statistically significant have higher odds of being fully reported.^[37]^ The nonpublication of research is a serious risk of bias, which may mislead those conducting SRs or relying on the published literature for evidence about health and social care.^[38]^ Hence, authors are required to explore and report any possible bias across studies.

The items discussed above were the main weakness to be blamed in the assessment of risk of bias and reporting quality, which could be avoided or reduced by using ROBIS or PRISMA as designing and reporting guidance. In order to achieve a more precise and convincing evidence, guiding by ROBIS or PRISMA to design, report, and assess SRs needs to be advocated.

### 4.3 Strengths and limitations

Firstly, this overview provides the latest evidence on acupuncture for KOA based on the findings of SRs, which indicated that acupuncture may have more total effective rate, short-term effective rate, and less adverse reactions in treating KOA than western medicine. Secondly, this overview is predesigned, which helps restrict the likelihood of biased decisions in reviewing. Thirdly, comprehensive search strategies were conducted for a wide range of data. Fourthly, independent reviewers were engaged in searching, screening, and assessing the potential studies and there was a high consistency among the reviewers.

Apart from the strengths, there are several limitations which may influence the reliability of our findings. There might be missing information since we only included studies written in English and Chinese. There may be duplicated clinical trials included by each SR that might have impact on the synthetic findings.

## 5 Conclusion

According to the high-quality evidence, we concluded that acupuncture may have some advantages in treating KOA. However, there are some risk of bias and reporting deficiencies still needed to improve.

## Acknowledgments

We would like to thank Professor Yao-Long Chen and Mr. Nan Yang from GRADE center in China (Lanzhou) for their assistance with the GRADE assessment.

## Author contributions

Literature search and data analysis: J.Y. and D.L.Z.

Manuscript preparation: J.L., Y.X.L., and L.J.L.

Critical revision of manuscript: Q.W.X, H.Z., and C.M.G.

All authors approved the publication of this study.

**Conceptualization:** Rong-Jiang Jin, Fan-Rong Liang.

**Data curation:** Jing Ye.

**Formal analysis:** Juan Li, Yu-Xi Li, Dong-Ling Zhong.

**Methodology:** Liao-Jun Luo.

**Writing – original draft:** Juan Li, Yu-Xi Li, Liao-Jun Luo.

**Writing – review & editing:** Qi-Wei Xiao, Hui Zheng, Chun-Mei Geng.

## Supplementary Material

Supplemental Digital Content
