# Supplementary material for: The effectiveness and safety of acupuncture for knee osteoarthritis: An overview of systematic reviews
Source: Medicine (Baltimore). 2019 Jul 12;98(28):e16301. doi: 10.1097/MD.0000000000016301 (PMC6641846; doi:10.1097/MD.0000000000016301)
Supplement: Supplemental Digital Content [file medi-98-e16301-s001.doc]

**Appendix 1. Search strategies**

MEDLINE

1. exp osteoarthritis/
2. osteoarthr$.tw.
3. (degenerative adj2 arthritis).tw.
4. arthrosis.tw.
5. or/1-4
6. Knee/
7. exp Knee Joint/
8. knee$.tw.
9. or/6-8

10. exp acupuncture/

11. exp acupuncture therapy/

12. exp acupuncture points/

13. exp acupuncture ear/

14. exp auriculotherapy/

15. exp electroacupuncture/

16. exp electric stimulation therapy/

17. exp acupressure/

18. exp moxibustion/

19. (acupuncture$ or electroacupuncture$).tw.

20. (acupuncture$ or moxibustion ).tw.

21. or/10-20

22.review.pt
23.meta analysis/

24.(systematic$ adj review$).tw

25.(meta analy$).tw

26.or/22-25

27. 5 and 9 and 21 and 26

EMBASE

1. exp osteoarthritis/
2. osteoarthr$.tw.
3. (degenerative adj2 arthritis).tw.
4. arthrosis.tw.
5. or/1-4
6. Knee/
7. knee$.tw.
8. 6 or 7

9. exp acupuncture/

10. exp electroacupuncture/

11. exp acupressure/

12. exp moxibustion/

13. (acupuncture $ or electroacupuncture $ ).tw.

14. ( acupuncture $ or moxibustion ).tw.

15.or/9-14

16. .review.pt
17.meta analysis/

18.(systematic$ adj review$).tw

19.(meta analy$).tw

20.or/16-19

21. 5 and 8 and 15 and 21

COCHRANE Library

1. MeSH descriptor Osteoarthritis explode all trees
2. (degenerative next arthritis):ti,ab
3. osteoarthr*:ti,ab
4. arthrosis:ti,ab
5. (#1 OR #2 OR #3 OR #4)
6. MeSH descriptor Knee explode all trees
7. MeSH descriptor Knee Joint explode all trees
8. knee*:ti,ab
9. (#6 OR #7 OR #8)

10. MeSH descriptor Acupuncture explode all trees

11. MeSH descriptor Acupuncture Therapy explode all trees

12. MeSH descriptor Acupuncture Points explode all trees

13. MeSH descriptor Acupuncture ,Ear explode all trees

14. MeSH descriptor Electroacupuncture explode all trees

15. MeSH descriptor Electric Stimulation Therapy explode all trees

16. MeSH descriptor Acupressure explode all trees

17. MeSH descriptor Moxibustion explode all trees

18. acupuncture* or electroacupuncture*

19. acupressure* or moxibustion

20. (#10 OR #11 OR #12 OR #13 OR #14 #15 OR #16 OR #17 OR #18 OR #19 )

21 . review.pt
22. meta analysis.pt

23. (systematic$ adj review$).tw

24. (meta analy$).tw

25. (#21 OR #22 OR #23 OR #24 )

26. (#5 and #9 and #20 and #25)

**Appendix 2. Excluded articles with reasons for exclusion**

| **Number** | **Reference** | **Reasons for exclusion** |
| --- | --- | --- |
|  | Puett DW, Griffin MR. Published trials of nonmedicinal and noninvasive therapies for hip and knee osteoarthritis. Annals of Internal Medicine 1994;121(2):133-40 | Contra to protocol: included studies involving patients with other chronic diseases and not a systematic review. |
|  | Deyo RAMDMPH. Drug Therapy for Back Pain: Which Drugs Help Which Patients? Spine 1996;21(24):2840-49 | Contra to protocol:Did not focused on knee osteoarthritis |
|  | Feine JSab, Lund JPacd. An assessment of the efficacy of physical therapy and physical modalities for the control of chronic musculoskeletal pain. Pain 1997;71(1):5-23 | Contra to protocol:included studies involving patients with other diseases. Interventions are not acupuncture and moxibustion. |
|  | Markovits EMD, Gilhar AMD. Capsaicin - an effective topical treatment in pain. International Journal of Dermatology 1997;36(6):401-04 | Contra to protocol：Study on Non-acupuncture or Non-moxibustion interventions |
|  | Feine JSDDSHDRa, Widmer CGDDSMSb, Lund JPBDSPc. Physical therapy: A critique. Oral Surgery, Oral Medicine, Oral Pathology, Oral Radiology, & Endodontics 1997;83(1):123-27 | Contra to protocol: Irrelevant to the gist of the article. |
|  | Balint G, Szebenyi B. Non-pharmacological therapies in osteoarthritis. Baillieres Clinical Rheumatology 1997;11(4):795-815 | Contra to protocol:not a systematic review |
|  | Brandt KD. The importance of nonpharmacologic approaches in management of osteoarthritis. American Journal of Medicine 1998;105(1B):39S-44S | Contra to protocol: included studies involving patients with other chronic diseases and not a systematic review. |
|  | Current World Literature. Current Opinion in Neurology 1998;11(5):563-615 | Contra to protocol: Irrelevant to the gist of the article. |
|  | Gordon AMD, Merenstein JHMD, D'Amico FP, et al. The Effects of Therapeutic Touch on Patients with Osteoarthritis of the Knee. Journal of Family Practice 1998;47(4):271-77 | Contra to protocol：Study on Non-acupuncture or Non-moxibustion interventions |
|  | Hsu DT, Diehl DL. The West gets the point. Lancet 1998;352 Supplement(IV):1SIV | Contra to protocol: Irrelevant to the gist of the article. |
|  | Creamer P, Flores R, Hochberg MC. Management of osteoarthritis in older adults. Clinics in Geriatric Medicine 1998;14(3):435-54 | Contra to protocol: included studies involving patients with other chronic diseases and not a systematic review. |
|  | Current World Literature. Current Opinion in Neurology 1999;12(6):771-821 | Contra to protocol: Irrelevant to the gist of the article. |
|  | Turturro MAMDF, Ducharme JMDCMF. Pain Management In The ED: Prompt, Cost-Effective, State-Of-The-Art Strategies. Emergency Medicine Practice 1999;1(7):1-15 | Contra to protocol: Irrelevant to the gist of the article. |
|  | Creamer PMDM. Osteoarthritis pain and its treatment. Current Opinion in Rheumatology 2000;12(5):450-55 | Contra to protocol:not a systematic review |
|  | American College Of Rheumatology Subcommittee On Osteoarthritis G. RECOMMENDATIONS FOR THE MEDICAL MANAGEMENT OF OSTEOARTHRITIS OF THE HIP AND KNEE: 2000 Update. Arthritis & Rheumatism 2000;43(9):1905-15 | Contra to protocol: included studies involving patients with other chronic diseases and not a systematic review. |
|  | Bouter LM. [Insufficient scientific evidence for efficacy of widely used electrotherapy, laser therapy, and ultrasound treatment in physiotherapy]. Nederlands Tijdschrift voor Geneeskunde 2000;144(11):502-5 | Contra to protocol:did not focused on knee osteoarthritis |
|  | *Bibliography Current World Literature. [Miscellaneous]*: Current Opinion in Rheumatology March 2000;12(2):B25, 2000 | Contra to protocol: Irrelevant to the gist of the article. |
|  | Osiri M, Welch V, Brosseau L, et al. Transcutaneous electrical nerve stimulation for knee osteoarthritis. Cochrane Database of Systematic Reviews 2000(4):CD002823 | Contra to protocol：Study on Non-acupuncture or Non-moxibustion interventions |
|  | Von Muhlen CA. Osteoarthritis. [Portuguese] Osteoartrose. Revista Brasileira de Medicina 2000;57(3):109-24 | Contra to protocol:not a systematic review |
|  | Burkhart SSMDD. A 26-Year-Old Woman With Shoulder Pain. JAMA 2000;284(12):1559-67 | Contra to protocol:did not focused on knee osteoarthritis |
|  | Della-Giustina DMDF, Kilcline BAMD, Denny MMD, et al. Back Pain: Cost-Effective Strategies For Distinguishing Between Benign And Life-Threatening Causes. Emergency Medicine Practice 2000;2(2):1-23 | Contra to protocol: Irrelevant to the gist of the article. |
|  | Creamer PMDM. Osteoarthritis pain and its treatment. [Miscellaneous]: Current Opinion in Rheumatology September 2000;12(5):450-455, 2000  . | Contra to protocol：Study on Non-acupuncture or Non-moxibustion interventions |
|  | Walker-Bone Kcrf, Javaid Kshoir, Arden Nslir, et al. Medical management of osteoarthritis. BMJ 2000;321(7266):936-40 | Contra to protocol:not a systematic review |
|  | Madhok Rcr, Kerr Hsrir, Capell HAcr. Rheumatology. BMJ 2000;321(7265):882-85 | Contra to protocol:did not focused on knee osteoarthritis |
|  | Felson DTMDMPH, Lawrence RCMPH, Hochberg MCMDMPH, et al. Osteoarthritis: New Insights: Part 2: Treatment Approaches. Annals of Internal Medicine 2000;133(9):726-37 | Contra to protocol: Irrelevant to the gist of the article. |
|  | Huang SHK. Rheumatology: 7. Basics of therapy. CMAJ Canadian Medical Association Journal 2000;163(4):417-23 | Contra to protocol: Irrelevant to the gist of the article. |
|  | Tallon D, Chard J, Dieppe P. Relation between agendas of the research community and the research consumer. Lancet 2000;355(9220):2037-40 | Contra to protocol: Irrelevant to the gist of the article. |
|  | Vickers Aaarm. Complementary medicine. BMJ 2000;321(7262):683-86 | Contra to protocol: Irrelevant to the gist of the article. |
|  | Atlas SJMDMPH, Deyo RAMDMPH. Evaluating and Managing Acute Low Back Pain in the Primary Care Setting. Journal of General Internal Medicine 2001;16(2):120-31 | Contra to protocol:did not focused on knee osteoarthritis |
|  | Easton BTMD. Evaluation and Treatment of the Patient with Osteoarthritis. Journal of Family Practice 2001;50(9):791-97 | Contra to protocol:not a systematic review |
|  | Recently completed postgraduate and honours research. Australian Journal of Physiotherapy 2001;47(1):75-79 | Contra to protocol: Irrelevant to the gist of the article. |
|  | Carlsson CPOMDPD, Sjolund BHMDPD. Acupuncture for Chronic Low Back Pain: A Randomized Placebo-Controlled Study With Long-Term Follow-Up. [Article]: Clinical Journal of Pain December 2001;17(4):296-305, 2001. | Contra to protocol:did not focused on knee osteoarthritis |
|  | White PFPMDF, Li SMDa, Chiu JWMBMD. Electroanalgesia: Its Role in Acute and Chronic Pain Management. Anesthesia & Analgesia 2001;92(2):505-13 | Contra to protocol:not a systematic review |
|  | Gentz BAM. Alternative Therapies for the Management of Pain in Labor and Delivery. [Miscellaneous]: Clinical Obstetrics & Gynecology December 2001;44(4):704-732, 2001. | Contra to protocol: Irrelevant to the gist of the article. |
|  | Crossley KBGDP, Bennell KBP, Green SBPGD, et al. A Systematic Review of Physical Interventions for Patellofemoral Pain Syndrome. [Review]: Clinical Journal of Sport Medicine April 2001;11(2):103-110, 2001. | Contra to protocol:did not focused on knee osteoarthritis |
|  | Wright APD, Sluka KAPD. Nonpharmacological Treatments for Musculoskeletal Pain. Clinical Journal of Pain 2001;17(1):33-46 | Contra to protocol:not a systematic review |
|  | Hochberg MC. What a difference a year makes: reflections on the ACR recommendations for the medical management of osteoarthritis. Current Rheumatology Reports 2001;3(6):473-8 | Contra to protocol: Irrelevant to the gist of the article. |
|  | Gentz BAM. Alternative Therapies for the Management of Pain in Labor and Delivery. Clinical Obstetrics & Gynecology 2001;44(4):704-32 | Contra to protocol:did not focused on knee osteoarthritis |
|  | McConnell S, Kolopack P, Davis AM. The Western Ontario and McMaster Universities Osteoarthritis Index (WOMAC): A Review of Its Utility and Measurement Properties. Arthritis & Rheumatism 2001;45(5) Arthritis Care &(Research):453-61 | Contra to protocol: Irrelevant to the gist of the article. |
|  | White PFPMDF, Li SMDa, Chiu JWMBMD. Electroanalgesia: Its Role in Acute and Chronic Pain Management. [Review]: Anesthesia & Analgesia February 2001;92(2):505-513, 2001 | Contra to protocol:did not focused on knee osteoarthritis |
|  | White AR, Filshie J, Cummings TM, et al. Clinical trials of acupuncture: consensus recommendations for optimal treatment, sham controls and blinding. Complementary Therapies in Medicine 2001;9(4):237-45 | Contra to protocol: Irrelevant to the gist of the article. |
|  | Wright APD, Sluka KAPD. Nonpharmacological Treatments for Musculoskeletal Pain. [Article]: Clinical Journal of Pain March 2001;17(1):33-46, 2001. | Contra to protocol: Irrelevant to the gist of the article. |
|  | Ernst EMDPF. Complementary and alternative medicine for pain management in rheumatic disease. Current Opinion in Rheumatology 2002;14(1):58-62 | Contra to protocol:did not focused on knee osteoarthritis |
|  | Dougados M, Doherty M, Pendleton A, et al. EULAR recommendations for the management of knee osteoarthritis. Report of a task force of the Standing Committee for International Clinical Studies Including Therapeutic Trials (ESCISIT). [German] Empfehlungen der EULAR zur behandlung der gonarthrose. Bericht einer kommission des "Standing Committee for International Clinical Studies Including Therapeutic Trials (ESCISIT)". Zeitschrift fur Rheumatologie 2002;61(3):229-43 | Contra to protocol:not a systematic review |
|  | Harris GR, Susman JL. Managing musculoskeletal complaints with rehabilitation therapy: summary of the Philadelphia Panel evidence-based clinical practice guidelines on musculoskeletal rehabilitation interventions. Journal of Family Practice 2002;51(12):1042-6 | Contra to protocol：Study on Non-acupuncture or Non-moxibustion interventions |
|  | Berman BMMD, Bausell RBP, Lee W-LP. Use and Referral Patterns for 22 Complementary and Alternative Medical Therapies by Members of the American College of Rheumatology : Results of a National Survey. Archives of Internal Medicine 2002;162(7):766-70 | Contra to protocol: Irrelevant to the gist of the article. |
|  | Kao CL, Chang JP. Pseudoaneurysm of the popliteal artery: a rare sequela of acupuncture. Texas Heart Institute Journal 2002;29(2):126-9 | Contra to protocol:did not focused on knee osteoarthritis |
|  | Ernst EMDPF. Complementary and alternative medicine for pain management in rheumatic disease. [Miscellaneous]: Current Opinion in Rheumatology January 2002;14(1):58-62, 2002. | Contra to protocol:not a systematic review |
|  | Hulme J, Robinson V, DeBie R, et al. Electromagnetic fields for the treatment of osteoarthritis. Cochrane Database of Systematic Reviews 2002(1):CD003523 | Contra to protocol：Study on Non-acupuncture or Non-moxibustion interventions |
|  | Ferrandez Infante A, Garcia Olmos L, Gonzalez Gamarra A, et al. [Effectiveness of acupuncture in the treatment of pain from osteoarthritis of the knee]. Atencion Primaria 2002;30(10):602-8; discussion 09-10 | Contra to protocol:not a systematic review |
|  | Sharma LMD. Nonpharmacologic management of osteoarthritis. [Miscellaneous]: Current Opinion in Rheumatology September 2002;14(5):603-607, 2002. | Contra to protocol:not a systematic review |
|  | Howard R. Late post-polio functional deterioration. Practical Neurology 2003;3(2):66-77 | Contra to protocol:did not focused on knee osteoarthritis |
|  | Flipo RM, Conrozier T. Current management of osteoarthritis part II: Non pharmacological strategies. [French] La prise en charge therapeutique de l'arthrose en ce debut de 3<sup>e</sup> millenaire seconde partie: Les traitements non medicamenteux. Revue de Medecine Interne 2003;24(4):243-52 | Contra to protocol:included studies involving patients with other diseases. Interventions are not acupuncture and moxibustion. |
|  | Cheing GLY, Tsui AYY, Lo SK, et al. OPTIMAL STIMULATION DURATION OF TENS IN THE MANAGEMENT OF OSTEOARTHRITIC KNEE PAIN. Journal of Rehabilitation Medicine 2003;35(2):62-68 | Contra to protocol:not a systematic review |
|  | Brosseau L, Yonge KA, Welch V, et al. Thermotherapy for treatment of osteoarthritis. Cochrane Database of Systematic Reviews 2003; (4). http://onlinelibrary.wiley.com/doi/10.1002/14651858.CD004522/abstract. | Contra to protocol：Study on Non-acupuncture or Non-moxibustion interventions |
|  | Boutron I, Tubach F, Giraudeau B, et al. Methodological Differences in Clinical Trials Evaluating Nonpharmacological and Pharmacological Treatments of Hip and Knee Osteoarthritis. Journal of the American Medical Association 2003;290(8):1062-70 | Contra to protocol: included studies involving patients with other chronic diseases and not a systematic review. |
|  | *II. REGIONAL ANESTHESIA. [Miscellaneous]*: Obstetric Anesthesia Digest July/September 2003;23(3):143-156, 2003. | Contra to protocol: Irrelevant to the gist of the article. |
|  | Katz NMD, Ju WDMD, Krupa DAMS, et al. Efficacy and Safety of Rofecoxib in Patients with Chronic Low Back Pain: Results from Two 4-Week, Randomized, Placebo-Controlled, Parallel-Group, Double-Blind Trials. [Miscellaneous Article]: Spine May 1, 2003;28(9):851-858, 2003. | Contra to protocol:did not focused on knee osteoarthritis |
|  | Grober JS, Thethi AK. Osteoarthritis: When are alternative therapies a good alternative? Consultant 2003;43(2):197-202 | Contra to protocol:included studies involving patients with other diseases. Interventions are not acupuncture and moxibustion. |
|  | Ehrlich GE. Global treatment of osteoarthritis. Inflammopharmacology 2003;11(4-5-6):333-36 | Contra to protocol:not a systematic review |
|  | Hochberg MC. Multidisciplinary Integrative Approach to Treating Knee Pain in Patients with Osteoarthritis. Annals of Internal Medicine 2003;139(9):781-83 | Contra to protocol：Study on Non-acupuncture or Non-moxibustion interventions |
|  | *The Adult Knee*, 2003. | Contra to protocol: Irrelevant to the gist of the article. |
|  | Licciardone JCDO, Stoll STDO, Fulda KGMPH, et al. Osteopathic Manipulative Treatment for Chronic Low Back Pain: A Randomized Controlled Trial. Spine 2003;28(13):1355-62 | Contra to protocol:did not focused on knee osteoarthritis |
|  | Hochberg MCMDMPH. Multidisciplinary Integrative Approach to Treating Knee Pain in Patients with Osteoarthritis. Annals of Internal Medicine 2003;139(9):781-83 | Contra to protocol:not a systematic review |
|  | Podichetty VK, Mazanec DJ, Biscup RS. Chronic non-malignant musculoskeletal pain in older adults: clinical issues and opioid intervention. Postgraduate Medical Journal 2003;79(937):627-33 | Contra to protocol：Study on Non-acupuncture or Non-moxibustion interventions |
|  | Ernst EMDPF. Complementary medicine. Current Opinion in Rheumatology 2003;15(2):151-55 | Contra to protocol: Irrelevant to the gist of the article. |
|  | Horng SBA, Miller FGP. Ethical framework for the use of sham procedures in clinical trials. Critical Care Medicine 2003;31(3)(Supplement):S126-S30 | Contra to protocol:not a systematic review |
|  | Strasser FMD, Driver LCMD, Burton AWMD. Update on Adjuvant Medications for Chronic Nonmalignant Pain. Pain Practice 2003;3(4):282-97 | Contra to protocol：Study on Non-acupuncture or Non-moxibustion interventions |
|  | Flipo RM, Conrozier T. [Therapeutic management of osteoarthritis in the beginning of the third millennium. Part II: non pharmacological strategies]. Revue de Medecine Interne 2003;24(4):243-52 | Contra to protocol: Irrelevant to the gist of the article. |
|  | Lonner JHMDD. A 57-Year-Old Man With Osteoarthritis of the Knee. JAMA 2003;289(8):1016-25 | Contra to protocol:not a systematic review |
|  | Taibi DMMSNRN, Bourguignon CPRN. The Role of Complementary and Alternative Therapies in Managing Rheumatoid Arthritis. [Miscellaneous]: Family & Community Health January 2003;26(1):41-52, 2003. | Contra to protocol：Study on Non-acupuncture or Non-moxibustion interventions |
|  | Horng SBA, Miller FGP. *Ethical framework for the use of sham procedures in clinical trials. [Review]*: Critical Care Medicine March 2003;31(3) Supplement:S126-S130, 2003. | Contra to protocol: Irrelevant to the gist of the article. |
|  | Markow MJ, Secor ER. Acupuncture for the pain management of osteoarthritis of the knee. Techniques in Orthopaedics 2003;18(1):33-36 | Contra to protocol:not a systematic review |
|  | Lewith GT, Breen A, Filshie J, et al. Complementary medicine: evidence base, competence to practice and regulation. Clinical Medicine May/June 2003;3(3):235-40 | Contra to protocol: Irrelevant to the gist of the article. |
|  | Taibi DMMSNRN, Bourguignon CPRN. The Role of Complementary and Alternative Therapies in Managing Rheumatoid Arthritis. Family & Community Health 2003;26(1):41-52 | Contra to protocol:not a systematic review |
|  | McAfee PC, Fedder IL, Saiedy S, et al. SB Charite Disc Replacement: Report of 60 Prospective Randomized Cases in a U.S. Center. [Article]: Journal of Spinal Disorders & Techniques Focus Issue: Disc Arthroplasty. August 2003;16(4):424-433, 2003. | Contra to protocol: Irrelevant to the gist of the article. |
|  | Niggemann B, Gruber C. Side-effects of complementary and alternative medicine. Allergy 2003;58(8):707-16 | Contra to protocol: Irrelevant to the gist of the article. |
|  | Tunis SRMDM, Stryer DBMD, Clancy CMMD. Practical Clinical Trials: Increasing the Value of Clinical Research for Decision Making in Clinical and Health Policy. JAMA 2003;290(12):1624-32 | Contra to protocol: Irrelevant to the gist of the article. |
|  | Birch S, Hesselink JK, Jonkman FAM, et al. Clinical research on acupuncture: Part 1. What have reviews of the efficacy and safety of acupuncture told us so far? Journal of Alternative and Complementary Medicine 2004;10(3):468-80 | Contra to protocol:did not focused on knee osteoarthritis |
|  | Madry H, Kohn D. Pharmacological and non-pharmacological treatment of knee osteoarthritis. [German] Konservative therapie der kniegelenkarthrose. Unfallchirurg 2004;107(8):689-700 | Contra to protocol:included studies involving patients with other diseases. Interventions are not acupuncture and moxibustion. |
|  | Berman BMMD, Lao LP, Langenberg PP, et al. Effectiveness of Acupuncture as Adjunctive Therapy in Osteoarthritis of the Knee: A Randomized, Controlled Trial. Annals of Internal Medicine 2004;141(12):901-10 | Contra to protocol:not a systematic review |
|  | Cheing GLY, Hui-Chan CWY. Would the addition of TENS to exercise training produce better physical performance outcomes in people with knee osteoarthritis than either intervention alone? Clinical Rehabilitation 2004;18(5):487-97 | Contra to protocol：Study on Non-acupuncture or Non-moxibustion interventions |
|  | Fan PT. Fibromyalgia and chronic fatigue syndrome. APLAR Journal of Rheumatology 2004;7(3):219-31 | Contra to protocol: included studies involving patients with other chronic diseases and not a systematic review. |
|  | *Adult & Pediatric Spine, The*, 2004. | Contra to protocol: Irrelevant to the gist of the article. |
|  | Denison BMSNAHNCQP. Touch the Pain Away: New Research on Therapeutic Touch and Persons With Fibromyalgia Syndrome. Holistic Nursing Practice May/June 2004;18(3):142-50 | Contra to protocol:did not focused on knee osteoarthritis |
|  | Denison BMSNAHNCQP. Touch the Pain Away: New Research on Therapeutic Touch and Persons With Fibromyalgia Syndrome. [Article]: Holistic Nursing Practice May/June 2004;18(3):142-150, 2004. | Contra to protocol: Irrelevant to the gist of the article. |
|  | Fitzgerald GK, Oatis C. Role of physical therapy in management of knee osteoarthritis. [Miscellaneous]: Current Opinion in Rheumatology March 2004;16(2):143-147, 2004. | Contra to protocol:not a systematic review |
|  | Fitzgerald Gnt Opinion in RheumatoloK, Oatis C. Role of physical therapy in management of knee osteoarthritis. Curregy 2004;16(2):143-47 | Contra to protocol：Study on Non-acupuncture or Non-moxibustion interventions |
|  | Schumacher HRJMD. Management Strategies for Osteoarthritis, Ankylosing Spondylitis, and Gouty Arthritis. JCR: Journal of Clinical Rheumatology 2004;10(3)(Supplement):S18-S25 | Contra to protocol: included studies involving patients with other chronic diseases and not a systematic review. |
|  | Birch S, Hesselink JK, Jonkman FA, et al. Clinical research on acupuncture. Part 1. What have reviews of the efficacy and safety of acupuncture told us so far? Journal of Alternative & Complementary Medicine 2004;10(3):468-80 | Contra to protocol: Irrelevant to the gist of the article. |
|  | Hurley DABMPMM, McDonough SMBPM, Dempster MBP, et al. A Randomized Clinical Trial of Manipulative Therapy and Interferential Therapy for Acute Low Back Pain. Spine 2004;29(20):2207-16 | Contra to protocol:did not focused on knee osteoarthritis |
|  | Goldenberg DLMD, Burckhardt CP, Crofford LMD. Management of Fibromyalgia Syndrome. JAMA 2004;292(19):2388-95 | Contra to protocol:not a systematic review |
|  | Weiner DKMD, Ernst EMDPF. Complementary and Alternative Approaches to the Treatment of Persistent Musculoskeletal Pain. Clinical Journal of Pain July/August 2004;20(4):244-55 | Contra to protocol: included studies involving patients with other chronic diseases and not a systematic review. |
|  | Hrycaj P. Serotonin type 3 receptor antagonist tropisetron in the treatment of chronic inflammatory rheumatic conditions - preliminary clinical experience. Scandinavian Journal of Rheumatology - Supplement 2004;33 Supplement(119):55-58 | Contra to protocol：Study on Non-acupuncture or Non-moxibustion interventions |
|  | Musich SA, Schultz AB, Burton WN, et al. Overview of Disease Management Approaches: Implications for Corporate-Sponsored Programs. Disease Management & Health Outcomes 2004;12(5):299-326 | Contra to protocol: Irrelevant to the gist of the article. |
|  | MacAuley Dgp. Managing osteoarthritis of the knee. BMJ 2004;329(7478):1300-01 | Contra to protocol:not a systematic review |
|  | Roos EM, Engstrom M, Lagerquist A, et al. Clinical improvement after 6 weeks of eccentric exercise in patients with mid-portion Achilles tendinopathy - a randomized trial with 1-year follow-up. Scandinavian Journal of Medicine & Science in Sports 2004;14(5):286-95 | Contra to protocol:did not focused on knee osteoarthritis |
|  | Vas Jcmo, Mendez Ce, Perea-Milla Ecmo, et al. Acupuncture as a complementary therapy to the pharmacological treatment of osteoarthritis of the knee: randomised controlled trial. BMJ 2004;329(7476):1216 | Contra to protocol:not a systematic review |
|  | Weiner DKMD, Ernst EMDPF. Complementary and Alternative Approaches to the Treatment of Persistent Musculoskeletal Pain. [Article]: Clinical Journal of Pain July/August 2004;20(4):244-255, 2004. | Contra to protocol：Study on Non-acupuncture or Non-moxibustion interventions |
|  | Schumacher HRJMD. Management Strategies for Osteoarthritis, Ankylosing Spondylitis, and Gouty Arthritis. [Article]: JCR: Journal of Clinical Rheumatology June 2004;10(3) Supplement:S18-S25, 2004. | Contra to protocol: Irrelevant to the gist of the article. |
|  | Soeken KLP. Selected CAM Therapies for Arthritis-Related Pain: The Evidence From Systematic Reviews. Clinical Journal of Pain January/February 2004;20(1):13-18 | Contra to protocol:included studies involving patients with other diseases and is a summary. |
|  | Soeken KLP. Selected CAM Therapies for Arthritis-Related Pain: The Evidence From Systematic Reviews. [Article]: Clinical Journal of Pain January/February 2004;20(1):13-18, 2004 | Contra to protocol:not a systematic review |
|  | Tasto JPMD, Cummings JMD, Medlock VMD, et al. Tendon Treatment Center. [Review]: Sports Medicine & Arthroscopy Review December 2004;12(4):210-219, 2004 | Contra to protocol: Irrelevant to the gist of the article. |
|  | Chow RTMBBSFMAS, Barnsley LBMPGDEFF. Systematic Review of the Literature of Low-Level Laser Therapy (LLLT) in the Management of Neck Pain. Lasers in Surgery & Medicine 2005;37(1):46-52 | Contra to protocol:did not focused on knee osteoarthritis |
|  | Ahmed S, Anuntiyo J, Malemud CJ, et al. Biological basis for the use of botanicals in osteoarthritis and rheumatoid arthritis: A review. Evidence-based Complementary and Alternative Medicine 2005;2(3):301-08 | Contra to protocol:not a systematic review |
|  | Horowitz S. Alternatives to COX-2 inhibitors for treating arthritis: Acupuncture and other traditional Chinese modalities. Alternative and Complementary Therapies 2005;11(4):173-78 | Contra to protocol: included studies involving patients with other chronic diseases and not a systematic review. |
|  | *Merritt's Neurology*, 2005. | Contra to protocol: Irrelevant to the gist of the article. |
|  | Dobkin BH. Rehabilitation after Stroke. New England Journal of Medicine 2005;352(16):1677-84 | Contra to protocol:did not focused on knee osteoarthritis |
|  | Bayat N, Keen HI, Hill CL. Randomized clinical trials of osteoarthritis: a review. APLAR Journal of Rheumatology 2005;8(3):171-76 | Contra to protocol:not a systematic review |
|  | Physical Medicine & Rehabilitation: Principles and Practice, 2005. | Contra to protocol: Irrelevant to the gist of the article. |
|  | Grabois MMD. Management of Chronic Low Back Pain. American Journal of Physical Medicine & Rehabilitation PAIN SCIENCE AND RATIONAL POLYPHARMACY 2005;84(3):S29-S41 | Contra to protocol:did not focused on knee osteoarthritis |
|  | Turek's Orthopaedics: Principles and Their Application, 2005. | Contra to protocol: Irrelevant to the gist of the article. |
|  | Howard RScn. Poliomyelitis and the postpolio syndrome. BMJ 2005;330(7503):1314-18 | Contra to protocol:did not focused on knee osteoarthritis |
|  | Arthritis & Allied Conditions, 2005. | Contra to protocol: Irrelevant to the gist of the article. |
|  | Fibromyalgia and Other Central Pain Syndromes, 2005 | Contra to protocol: Irrelevant to the gist of the article. |
|  | Chard J, Lohmander S, Smith C, et al. Osteoarthritis of the knee. Clinical Evidence 2005(14):1506-22 | Contra to protocol:not a systematic review |
|  | Bodnar RJ, Klein GE. Endogenous opiates and behavior: 2004. Peptides 2005;26(12):2629-711 | Contra to protocol: Irrelevant to the gist of the article. |
|  | Fitzcharles M-AMBCF, Almahrezi AMBC, Shir YMD. Pain: Understanding and Challenges for the Rheumatologist. Arthritis & Rheumatism 2005;52(12):3685-92 | Contra to protocol: Irrelevant to the gist of the article. |
|  | Hadler NM. Occupational Musculoskeletal Disorders, 2005. | Contra to protocol: Irrelevant to the gist of the article. |
|  | Kim Y-HMDMPHD. Efficacy of Acupuncture for Treating Knee Osteoarthritis. Alternative Medicine Alert 2005;8(5):49-53 | Contra to protocol:not a systematic review |
|  | Katz WAMD, Rothenberg RMD. Section 4: Treating the Patient in Pain. [Article]: JCR: Journal of Clinical Rheumatology DIALOGUES IN RHEUMATOLOGY: Reconsidering Pain Management for Patients With Rheumatic Diseases and the Role of Atypical Opioids. April 2005;11(2) Supplement:S16-S28, 2005. | Contra to protocol: Irrelevant to the gist of the article. |
|  | Lucas B. Treatment options for patients with osteoarthritis of the knee. British Journal of Nursing 2005;14(18):976-81 | Contra to protocol:not a systematic review |
|  | Keefe FJ, Abernethy AP, Campbell LC. Psychological Approaches to Understanding and Treating Disease-Related Pain. Annual Review of Psychology 2005;56:601-30 | Contra to protocol: Irrelevant to the gist of the article. |
|  | McQuay HJ, Moore RA. Placebo. Postgraduate Medical Journal 2005;81(953):155-60 | Contra to protocol:not a systematic review |
|  | Lehman RMM. Evidently. Evidence Based Medicine 2005;10(5):135 | Contra to protocol: Irrelevant to the gist of the article. |
|  | Wu SS, Tuan K. Current concepts in nonoperative management of knee osteoarthritis. Orthopedics 2005;28(2):134-9; quiz 40-1 | Contra to protocol:not a systematic review |
|  | Samanta AMDF, Samanta JBA, Johnson MRDMAPCHE, et al. Rheumatoid arthritis in minority ethnic groups: patterns of disease, clinical and sociocultural features among British South Asians. Diversity in Health & Social Care 2005;2(2):99-118 | Contra to protocol: Irrelevant to the gist of the article. |
|  | Lewith GTMADMFM, White PJP, Kaptchuk TJ. Developing a Research Strategy for Acupuncture. [Review]: Clinical Journal of Pain September 2006;22(7):632-638, 2006. | Contra to protocol:did not focused on knee osteoarthritis |
|  | Current World Literature. [Article]: Current Opinion in Orthopaedics February 2006;17(1):68-84, 2006. | Contra to protocol:not a systematic review |
|  | Bellamy N, Campbell J, Welch V, et al. Viscosupplementation for the treatment of osteoarthritis of the knee. Cochrane Database of Systematic Reviews 2006; (2). http://onlinelibrary.wiley.com/doi/10.1002/14651858.CD005321.pub2/abstract. | Contra to protocol：Study on Non-acupuncture or Non-moxibustion interventions |
|  | Linde K, Streng A, Hoppe A, et al. The programme for the evaluation of patient care with acupuncture (PEP-Ac) - A project sponsored by ten German social health insurance funds. Acupuncture in Medicine 2006;24(SUPPL.):S25-S32 | Contra to protocol: included studies involving patients with other chronic diseases and not a systematic review. |
|  | Acupuncture. [Letter]: Obstetrics & Gynecology October 2006;108(4):1024-1026, 2006. | Contra to protocol: Irrelevant to the gist of the article. |
|  | McCarthy CJ, Callaghan MJ, Oldham JA. Pulsed electromagnetic energy treatment offers no clinical benefit in reducing the pain of knee osteoarthritis: a systematic review. BMC Musculoskeletal Disorders 2006;7:51 | Contra to protocol:did not focused on knee osteoarthritis |
|  | Bjordal JMprf, Lopes-Martins RABap, Bogen Bp, et al. Physical treatments have valuable role in osteoarthritis. BMJ 2006;332(7545):853 | Contra to protocol:not a systematic review |
|  | Bjordal J. NSAIDs in osteoarthritis: irreplaceable or troublesome guidelines? British Journal of Sports Medicine 2006;40(4):285-86 | Contra to protocol：Study on Non-acupuncture or Non-moxibustion interventions |
|  | Current World Literature. Current Opinion in Orthopaedics 2006;17(1):68-84 | Contra to protocol: Irrelevant to the gist of the article. |
|  | Wadsworth LTMD. Acupuncture in Sports Medicine. Current Sports Medicine Reports 2006;5(1):1-3 | Contra to protocol:did not focused on knee osteoarthritis |
|  | Brinkhaus B, Witt CM, Linde K, et al. Efficacy of acupuncture in patients with osteoarthritis of the knee. A randomized controlled trial. [German]  Wirksamkeit der akupunkturbehandlung bei osteoarthrose. Ergebnisse einer randomisierten kontrollierten studie. Internistische Praxis 2006;46(3):637-47 | Contra to protocol:not a systematic review |
|  | Distler JMSFNPCDPAF, Anguelouch AMSNFNPC. Evidence-based practice: Review of clinical evidence on the efficacy of glucosamine and chondroitin in the treatment of osteoarthritis. Journal of the American Academy of Nurse Practitioners 2006;18(10):487-93 | Contra to protocol：Study on Non-acupuncture or Non-moxibustion interventions |
|  | Office Care Geriatrics, 2006. | Contra to protocol: Irrelevant to the gist of the article. |
|  | Cassileth BRPF, Deng GEMDP, Gomez JEMD, et al. Complementary Therapies and Integrative Oncology in Lung Cancer*: ACCP Evidence-Based Clinical Practice Guidelines (2nd Edition). Chest Diagnosis and Management of Lung Cancer: ACCP Evidence-Based Clinical Practice Guidelines (2nd Edition) 2007;132(3):340S-54S | Contra to protocol:did not focused on knee osteoarthritis |
|  | Derry CJ, Derry S, McQuay HJ, et al. Systematic review of systematic reviews of acupuncture published 1996-2005. Clinical Medicine July/August 2006;6(4):381-86 | Contra to protocol:not a systematic review |
|  | Psychosomatic Medicine, 2006. | Contra to protocol: Irrelevant to the gist of the article. |
|  | Hospital for Special Surgery Manual of Rheumatology and Outpatient Orthopedic Disorders: Diagnosis and Therapy, 2006. | Contra to protocol: Irrelevant to the gist of the article. |
|  | . Ernst E. Acupuncture - a critical analysis. Journal of Internal Medicine 2006;259(2):125-37 | Contra to protocol:not a systematic review |
|  | Archibeck MJMD, White REJMD. What's New in Adult Reconstructive Knee Surgery. Journal of Bone & Joint Surgery - American Volume 2006;88(7):1677-86 | Contra to protocol: Irrelevant to the gist of the article. |
|  | Ernst E. Complementary or alternative therapies for osteoarthritis. Nature Clinical Practice Rheumatology 2006;2(2):74-80 | Contra to protocol:not a systematic review |
|  | Bodnar RJ, Klein GE. Endogenous opiates and behavior: 2005. Peptides 2006;27(12):3391-478 | Contra to protocol: Irrelevant to the gist of the article. |
|  | Felson DT. Osteoarthritis of the Knee. New England Journal of Medicine 2006;354(8):841-48 | Contra to protocol:not a systematic review |
|  | Fritz J, Gaissmaier C, Schewe B, et al. Cartilage repair in the knee joint. [German]  Biologische knorpelrekonstruktion im kniegelenk. Unfallchirurg 2006;109(7):563-76 | Contra to protocol:not a systematic review |
|  | Breivik H, Collett B, Ventafridda V, et al. Survey of chronic pain in Europe: Prevalence, impact on daily life, and treatment. European Journal of Pain 2006;10(4):287-333 | Contra to protocol: Irrelevant to the gist of the article. |
|  | Garrow Jrp, former chairman of H. Alternative Medicine. BMJ 2006;332(7535):241 | Contra to protocol:not a systematic review |
|  | Caprilli R, Gassull MA, Escher JC, et al. European evidence based consensus on the diagnosis and management of Crohn's disease: special situations. Gut 2006;55 Supplement(1):i36-i58 | Contra to protocol: Irrelevant to the gist of the article. |
|  | Hoskins WMa, McHardy AMb, Pollard HGDCPc, et al. CHIROPRACTIC TREATMENT OF LOWER EXTREMITY CONDITIONS: A LITERATURE REVIEW. Journal of Manipulative & Physiological Therapeutics 2006;29(8):658-71 | Contra to protocol:not a systematic review |
|  | Lewith G, Berman B, Cummings M, et al. Systematic review of systematic reviews of acupuncture published 1996-2005. Clinical Medicine November/December 2006;6(6):623-25 | Contra to protocol:not a systematic review |
|  | Curtis LAMDF, Morrell TDMD, Godwin SAMDF, et al. Pain Management in the Emergency Department. Emergency Medicine Practice 2006;8(7):1-26 | Contra to protocol: Irrelevant to the gist of the article. |
|  | Linde K, Weidenhammer W, Streng A, et al. Acupuncture for osteoarthritic pain: an observational study in routine care. Rheumatology 2006;45(2):222-27 | Contra to protocol:not a systematic review |
|  | Evans CJ, Parthan A, Le K. Economic and humanistic burden of fibromyalgia in the USA. Expert Review of Pharmacoeconomics & Outcomes Research 2006;6(3):303-14 | Contra to protocol: Irrelevant to the gist of the article. |
|  | Lund I, Lundeberg T. Are minimal, superficial or sham acupuncture procedures acceptable as inert placebo controls? Acupuncture in Medicine 2006;24(1):13-5 | Contra to protocol:not a systematic review |
|  | Melchart D, Streng A, Hoppe A, et al. Acupuncture for chronic pain - Results from the research program of ten health insurance funds. [German]  Akupunkture bei chronischen Schmerzen - Ergebnisse aus dem Modellvorhaben der Ersatzkassen. Deutsches Arzteblatt 2006;103(4):A187-95 | Contra to protocol:not a systematic review |
|  | Lewith GTMADMFM, White PJP, Kaptchuk TJ. Developing a Research Strategy for Acupuncture. Clinical Journal of Pain 2006;22(7):632-38 | Contra to protocol: Irrelevant to the gist of the article. |
|  | Shaughnessy AFapoph, family m, Gordon AEcapoph, et al. Life without COX 2 inhibitors. BMJ 2006;332(7553):1287-88 | Contra to protocol: Irrelevant to the gist of the article. |
|  | Ravaud P, Boutron I. Primer: assessing the efficacy and safety of nonpharmacologic treatments for chronic rheumatic diseases. Nature Clinical Practice Rheumatology 2006;2(6):313-19 | Contra to protocol:not a systematic review |
|  | Roberts JR. A mapping exercise of the systematic review evidence of effectiveness for acupuncture. Focus on Alternative & Complementary Therapies an Evidence-Based Approach 2006;11 Supplement(1):40 | Contra to protocol:not a systematic review |
|  | Cohen SPMD, Raja SNMD. Pathogenesis, Diagnosis, and Treatment of Lumbar Zygapophysial (Facet) Joint Pain. Anesthesiology 2007;106(3):591-614 | Contra to protocol:did not focused on knee osteoarthritis |
|  | Roddy E, Doherty M. Changing life-styles and osteoarthritis: What is the evidence? Best Practice and Research: Clinical Rheumatology 2006;20(1):81-97 | Contra to protocol:not a systematic review |
|  | Scharf H-PMD, Mansmann UP, Streitberger KMD, et al. Acupuncture and Knee Osteoarthritis: A Three-Armed Randomized Trial. Annals of Internal Medicine 2006;145(1):12-20 | Contra to protocol:not a systematic review |
|  | White A, Tough E, Cummings M. A review of acupuncture clinical trials indexed during 2005. Acupuncture in Medicine 2006;24(1):39-49 | Contra to protocol:not a systematic review |
|  | Witt CM, Jena S, Brinkhaus B, et al. Acupuncture in Patients with Osteoarthritis of the Knee or Hip: A randomized, controlled trial with an additional nonrandomized arm. Arthritis & Rheumatism 2006;54(11):3485-93 | Contra to protocol:not a systematic review |
|  | Lane NE. Osteoarthritis of the Hip. New England Journal of Medicine 2007;357(14):1413-21 | Contra to protocol:did not focused on knee osteoarthritis |
|  | Acupuncture. Focus on Alternative & Complementary Therapies an Evidence-Based Approach 2007;12(4):294 | Contra to protocol:not a systematic review |
|  | Bodnar RJ. Endogenous opiates and behavior: 2006. Peptides 2007;28(12):2435-513 | Contra to protocol：Study on Non-acupuncture or Non-moxibustion interventions |
|  | Jones K, Piterman L. Promoting best practice in general practitioner management of osteoarthritis of the hip and knee: Arthritis and Musculoskeletal Quality Improvement Program (AMQuIP). Australian Journal of Primary Health 2007;13(2):104-12 | Contra to protocol: included studies involving patients with other chronic diseases and not a systematic review. |
|  | FULL TEXT Chapter 1: Introduction. European Journal of Cardiovascular Prevention & Rehabilitation 2007;14 Supplement(2):S2-S113 | Contra to protocol: Irrelevant to the gist of the article. |
|  | Mazanec DMD, Reddy AMD. MEDICAL MANAGEMENT OF CERVICAL SPONDYLOSIS. Neurosurgery DIAGNOSIS AND TREATMENT OF CERVICAL SPONDYLOSIS 2007;60(1):S1-43-S1-50 | Contra to protocol:did not focused on knee osteoarthritis |
|  | Barron MC, Rubin BR. Managing osteoarthritic knee pain. Journal of the American Osteopathic Association 2007;107(10 Suppl 6):ES21-7 | Contra to protocol:not a systematic review |
|  | Broughton GIIMDPDCMCUSA, Crosby MAMD, Coleman JMD, et al. Use of Herbal Supplements and Vitamins in Plastic Surgery: A Practical Review. Plastic & Reconstructive Surgery 2007;119(3):48e-66e | Contra to protocol：Study on Non-acupuncture or Non-moxibustion interventions |
|  | THIS WEEK IN JAMA: APRIL 18, 2007. JAMA 2007;297(15):1625 | Contra to protocol: Irrelevant to the gist of the article. |
|  | Berman B. A 60-year-old woman considering acupuncture for knee pain. JAMA 2007;297(15):1697-707 | Contra to protocol:not a systematic review |
|  | Guindon J, Walczak J-S, Beaulieu P. Recent Advances in the Pharmacological Management of Pain. Drugs 2007;67(15):2121-33 | Contra to protocol：Study on Non-acupuncture or Non-moxibustion interventions |
|  | My Knee is Stuck!! Acupuncture and OA. Alternative Medicine Alert 2007;10(10):118-19 | Contra to protocol: Irrelevant to the gist of the article. |
|  | Kang RWMDMS, Lewis PBMDMS, Kramer AATC, et al. Prospective Randomized Single-blinded Controlled Clinical Trial of Percutaneous Neuromodulation Pain Therapy Device Versus Sham for the Osteoarthritic Knee: A Pilot Study. Orthopedics 2007;30(6):439-45 | Contra to protocol：Study on Non-acupuncture or Non-moxibustion interventions |
|  | Principles & Practice of Palliative Care & Supportive Oncology, 2007. | Contra to protocol: Irrelevant to the gist of the article. |
|  | Cohen SPMD, Raja SNMD. Pathogenesis, Diagnosis, and Treatment of Lumbar Zygapophysial (Facet) Joint Pain. [Review]: Anesthesiology March 2007;106(3):591-614, 2007. | Contra to protocol:not a systematic review |
|  | Dowd GSE, Hussein R, Khanduja V, et al. Complex regional pain syndrome with special emphasis on the knee. Journal of Bone & Joint Surgery - British Volume 2007;89-B(3):285-90 | Contra to protocol:not a systematic review |
|  | Herbst KLMDP, Asare-Bediako SBS. Adiposis Dolorosa Is More Than Painful Fat. [Review]: Endocrinologist November/December 2007;17(6):326-334, 2007. | Contra to protocol: Irrelevant to the gist of the article. |
|  | Michel J. J. Hooiveld GR, Marieke E. Vianen, H. Marijke Van Den Berg, Johannes W. J. . Spa water more effective than tap water for knee osteoarthritis. Focus on Alternative & Complementary Therapies an Evidence-Based Approach 2007;12(2):124 | Contra to protocol：Study on Non-acupuncture or Non-moxibustion interventions |
|  | Principles of Ambulatory Medicine, 2007. | Contra to protocol: Irrelevant to the gist of the article. |
|  | Foster NEslit, Thomas Eslib, Barlas Prf, et al. Acupuncture as an adjunct to exercise based physiotherapy for osteoarthritis of the knee: randomised controlled trial. BMJ 2007;335(7617):436-47 | Contra to protocol:not a systematic review |
|  | Porcheret M, Jordan K, Croft P, et al. Treatment of knee pain in older adults in primary care: development of an evidence-based model of care. Rheumatology 2007;46(4):638-48 | Contra to protocol：Study on Non-acupuncture or Non-moxibustion interventions |
|  | Rehabilitation of the Spine: A Practitioner's Manual, 2007. | Contra to protocol: Irrelevant to the gist of the article. |
|  | Herbert Rap, Fransen Msrf. Management of chronic knee pain. BMJ 2007;335(7624):786 | Contra to protocol:not a systematic review |
|  | Porcheret M, Jordan K, Jinks C, et al. Primary care treatment of knee pain-a survey in older adults. Rheumatology 2007;46(11):1694-700 | Contra to protocol：Study on Non-acupuncture or Non-moxibustion interventions |
|  | 5-Minute Orthopaedic Consult, 2007. | Contra to protocol: Irrelevant to the gist of the article. |
|  | Herbst KLMDP, Asare-Bediako SBS. Adiposis Dolorosa Is More Than Painful Fat. Endocrinologist November/December 2007;17(6):326-34 | Contra to protocol:not a systematic review |
|  | Touma Z, Chen L, Arayssi T. Topical nonsteroidal anti-inflammatory drugs in the treatment of osteoarthritis. Future Rheumatology 2007;2(2):163-75 | Contra to protocol：Study on Non-acupuncture or Non-moxibustion interventions |
|  | Bratton RL. Bratton's Family Medicine Board Review, 2007. | Contra to protocol: Irrelevant to the gist of the article. |
|  | . Ibanez AE. The traditional Chinese acupuncture does not improve the symptomatology of knee osteoarthritis. [Spanish]  La acupuntura tradicional china no mejora la sintomatologia de la artrosis de rodilla. FMC Formacion Medica Continuada en Atencion Primaria 2007;14(9):585 | Contra to protocol:not a systematic review |
|  | Zhang W, Moskowitz RW, Nuki G, et al. OARSI recommendations for the management of hip and knee osteoarthritis, Part I: Critical appraisal of existing treatment guidelines and systematic review of current research evidence. Osteoarthritis and Cartilage 2007;15(9):981-1000 | Contra to protocol：Study on Non-acupuncture or Non-moxibustion interventions |
|  | Broughton GIIMDPDCMCUSA, Crosby MAMD, Coleman JMD, et al. Use of Herbal Supplements and Vitamins in Plastic Surgery: A Practical Review. [Miscellaneous]: Plastic & Reconstructive Surgery March 2007;119(3):48e-66e, 2007. | Contra to protocol: Irrelevant to the gist of the article. |
|  | Lev-Ari S, Hasner A, Amir H, et al. Acupuncture as complementary medicine in patients with osteoarthritis of the knee. [Hebrew]. Harefuah 2007;146(5):354-57, 406 | Contra to protocol:not a systematic review |
|  | Callaghan JJ. The Adult Hip, 2007. | Contra to protocol: Irrelevant to the gist of the article. |
|  | Li L, Li N, Wu B. [Bibliometric analysis of literature on acupuncture and moxibustion for treatment of knee osteoarthritis]. Zhongguo Zhenjiu 2007;27(11):862-4 | Contra to protocol:not a systematic review |
|  | Combe B, Landewe R, Lukas C, et al. EULAR recommendations for the management of early arthritis: report of a task force of the European Standing Committee for International Clinical Studies Including Therapeutics (ESCISIT). Annals of the Rheumatic Diseases 2007;66(1):34-45 | Contra to protocol: Irrelevant to the gist of the article. |
|  | Linde Ka, Witt CMb, Streng Aa, et al. The impact of patient expectations on outcomes in four randomized controlled trials of acupuncture in patients with chronic pain. [Article]: Pain April 2007;128(3):264-271, 2007. | Contra to protocol:not a systematic review |
|  | Hurley M, Bearne LM. Physiotherapy for musculoskeletal conditions: more difficult than rocket science. Future Rheumatology 2007;2(2):185-92 | Contra to protocol: Irrelevant to the gist of the article. |
|  | Porcheret M, Jordan K, Croft P. Treatment of knee pain in older adults in primary care: Development of an evidence-based model of care. Rheumatology 2007;46(4):638-48 | Contra to protocol:not a systematic review |
|  | Li L, Li N, Wu B. Bibliometric analysis of literature on acupuncture and moxibustion for treatment of knee osteoarthritis. [Chinese]. Zhongguo zhen jiu = Chinese acupuncture & moxibustion 2007;27(11):862-64 | Contra to protocol: Original from Chinese database |
|  | Scott D, Kowalczyk A. Osteoarthritis of the knee. Clinical Evidence 2007;01:01 | Contra to protocol:not a systematic review |
|  | Luz FBMDP, Gaspar NKMDP, Gaspar APMDP, et al. Multicentric Reticulohistiocytosis: A Proliferation of Macrophages With Tropism for Skin and Joints, Part II. SKINmed September/October 2007;6(5):227-33 | Contra to protocol: Irrelevant to the gist of the article. |
|  | Staud R. Mechanisms of acupuncture analgesia: effective therapy for musculoskeletal pain? Current Rheumatology Reports 2007;9(6):473-81 | Contra to protocol:not a systematic review |
|  | Tsang RC-C, Tsang P-L, Ko C-Y, et al. Effects of acupuncture and sham acupuncture in addition to physiotherapy in patients undergoing bilateral total knee arthroplasty - a randomized controlled trial. Clinical Rehabilitation 2007;2(8):719-28 | Contra to protocol:not a systematic review |
|  | Miller FG, Kaptchuk TJ. Acupuncture trials and informed consent. Journal of Medical Ethics 2007;33(1):43-44 | Contra to protocol: Irrelevant to the gist of the article. |
|  | Moskowitz RW, Altman RD, Hochberg MC, et al. Osteoarthritis: Diagnosis and Medical/Surgical Management, 2007. | Contra to protocol: Irrelevant to the gist of the article. |
|  | Vas J, White A. Evidence from RCTs on optimal acupuncture treatment for knee osteoarthritis--an exploratory review. Acupuncture in Medicine 2007;25(1-2):29-35 | Contra to protocol:not a systematic review |
|  | Yap EC. Myofascial pain - An overview. Annals of the Academy of Medicine Singapore 2007;36(1):43-48 | Contra to protocol:not a systematic review |
|  | Pavelka K. World Congress on Osteoarthritis (Osteoarthritis Research Society International). Future Rheumatology 2007;2(2):129-32 | Contra to protocol: Irrelevant to the gist of the article. |
|  | Stratton TDP, Benn RKP, Lie DAMDM, et al. Evaluating CAM Education in Health Professions Programs. Academic Medicine 2007;82(10):956-61 | Contra to protocol: Irrelevant to the gist of the article. |
|  | Wilkinson JMBCMF, Faleiro RBDCHF. Acupuncture in pain management. Continuing Education in Anaesthesia, Critical Care & Pain 2007;7(4):135-38 | Contra to protocol: Irrelevant to the gist of the article. |
|  | 鶴岡 浩, 中村,行雄. Acupuncture (real or minimal) works better than conservative therapy in chronic knee osteoarthritis. Focus on Alternative & Complementary Therapies an Evidence-Based Approach 2007;12(1):38-39 | Contra to protocol:not a systematic review |
|  | Stratton TDP, Benn RKP, Lie DAMDM, et al. Evaluating CAM Education in Health Professions Programs. [Miscellaneous]: Academic Medicine October 2007;82(10):956-961, 2007 | Contra to protocol: Irrelevant to the gist of the article. |
|  | Endres HGMD, Molsberger APMD, Haake MPMD. Acupuncture Ineffective, Attention Effective? Archives of Internal Medicine 2008;168(5):551-52 | Contra to protocol:did not focused on knee osteoarthritis |
|  | Acupuncture. Focus on Alternative & Complementary Therapies an Evidence-Based Approach 2008;13(4):300 | Contra to protocol:not a systematic review |
|  | Zhou JH, Wu YC. Acupuncture and moxibustion for knee osteoarthritis: Five-year data review. [Chinese]. Journal of Clinical Rehabilitative Tissue Engineering Research 2008;12(7):1337-40 | Contra to protocol: Original from Chinese database |
|  |  |  |
|  | Wang S-MMD, Kain ZNMDMBA, White PFPMDF. Acupuncture Analgesia: II. Clinical Considerations. Anesthesia & Analgesia 2008;106(2):611-21 | Contra to protocol:not a systematic review |
|  | Silverman SLMDFF. Paget Disease of Bone: Therapeutic Options. [Review]: JCR: Journal of Clinical Rheumatology October 2008;14(5):299-305, 2008. | Contra to protocol: Irrelevant to the gist of the article. |
|  | Basford JR, Baxter DG. Low level laser therapy: current status. Focus on Alternative & Complementary Therapies an Evidence-Based Approach 2008;13(1):11-13 | Contra to protocol：Study on Non-acupuncture or Non-moxibustion interventions |
|  | Selfe TKDCP, Taylor AGERNF. Acupuncture and Osteoarthritis of the Knee: A Review of Randomized, Controlled Trials. Family & Community Health Complementary Practice and Products July/September 2008;31(3):247-54 | Contra to protocol:not a systematic review |
|  | Wang S-MMD, Kain ZNMDMBA, White PFPMDF. Acupuncture Analgesia: II. Clinical Considerations. [Review]: Anesthesia & Analgesia February 2008;106(2):611-621, 2008. | Contra to protocol: Irrelevant to the gist of the article. |
|  | Brauer S. Hip and knee osteoarthritis. Australian Journal of Physiotherapy 2008;54(4):286 | Contra to protocol: included studies involving patients with other chronic diseases and not a systematic review. |
|  | Maa S-H, Sun M-F, Wu C-C. The Effectiveness of Acupuncture on Pain and Mobility in Patients With Osteoarthritis of the Knee: A Pilot Study. [Article]: Journal of Nursing Research June 2008;16(2):140-148, 2008 | Contra to protocol:not a systematic review |
|  | WESTERN REGIONAL MEETING AT A GLANCE. [Abstract]: Journal of Investigative Medicine January 2008;56(1):103-267, 2008. | Contra to protocol: Irrelevant to the gist of the article. |
|  | Selfe TKDCP, Taylor AGERNF. Acupuncture and Osteoarthritis of the Knee: A Review of Randomized, Controlled Trials. [Article]: Family & Community Health Complementary Practice and Products. July/September 2008;31(3):247-254, 2008 | Contra to protocol:not a systematic review |
|  | Goldman RHMDMPH, Stason WBMD, Park SKS, et al. Acupuncture for Treatment of Persistent Arm Pain Due to Repetitive Use: A Randomized Controlled Clinical Trial. [Article]: Clinical Journal of Pain March/April 2008;24(3):211-218, 2008. | Contra to protocol:did not focused on knee osteoarthritis |
|  | Rosemann T. Evidence based therapy of degenerative joint diseases - Surgical treatment options. [German]  Evidenzbasierte therapie degenerativer gelenkerkrankungen: Teil 1: Allgemeine und operative therapieverfahren. Zeitschrift fur Allgemeinmedizin 2008;84(1):21-27 | Contra to protocol:not a systematic review |
|  | Reid MCMDP, Papaleontiou MMD, Ong AP, et al. Self-Management Strategies to Reduce Pain and Improve Function among Older Adults in Community Settings: A Review of the Evidence. Pain Medicine May/June 2008;9(4):409-24 | Contra to protocol: Irrelevant to the gist of the article. |
|  | Bijlsma JWJ, Welsing PMJ. The art of medicine in treating osteoarthritis: I will please. Annals of the Rheumatic Diseases 2008;67(12):1653-55 | Contra to protocol:not a systematic review |
|  | Park J, Linde K, Manheimer E, et al. The status and future of acupuncture clinical research. Journal of Alternative & Complementary Medicine 2008;14(7):871-81 | Contra to protocol: Irrelevant to the gist of the article. |
|  | Khan NMD, Husain SMD, Haak MMD. Thoracolumbar Injuries in the Athlete. [Review]: Sports Medicine & Arthroscopy Review March 2008;16(1):16-25, 2008 | Contra to protocol: Irrelevant to the gist of the article. |
|  | Pittler MH, Ernst E. Is acupuncture an effective treatment for knee osteoarthritis? [Miscellaneous]: Nature Clinical Practice Rheumatology March 2008;4(3):124-125, 2008. | Contra to protocol:not a systematic review |
|  | Svensson P, Jadidi F, Arima T, et al. Relationships between craniofacial pain and bruxism *. Journal of Oral Rehabilitation 2008;35(7):524-47 | Contra to protocol：Study on Non-acupuncture or Non-moxibustion interventions |
|  | Zhang W, Moskowitz RW, Nuki G, et al. OARSI recommendations for the management of hip and knee osteoarthritis, Part II: OARSI evidence-based, expert consensus guidelines. Osteoarthritis & Cartilage 2008;16(2):137-62 | Contra to protocol: Irrelevant to the gist of the article. |
|  | Das SK, Farooqi A. Osteoarthritis. Best Practice and Research: Clinical Rheumatology 2008;22(4):657-75 | Contra to protocol: included studies involving patients with other chronic diseases and not a systematic review. |
|  | Other articles noted. Evidence Based Medicine 2008;13(3):95-96 | Contra to protocol: Irrelevant to the gist of the article. |
|  | Hernandez-Molina G, Reichenbach S, Zhang BIN, et al. Effect of Therapeutic Exercise for Hip Osteoarthritis Pain: Results of a Meta-Analysis. Arthritis & Rheumatism Arthritis Care & Research 2008;59(9):1221-28 | Contra to protocol:did not focused on knee osteoarthritis |
|  | Meier W, Mizner RL, Marcus RL, et al. Total knee arthroplasty: muscle impairments, functional limitations, and recommended rehabilitation approaches. Journal of Orthopaedic & Sports Physical Therapy 2008;38(5):246-56 | Contra to protocol:not a systematic review |
|  | Oken BS. Placebo effects: clinical aspects and neurobiology. Brain 2008;131(11):2812-23 | Contra to protocol: Irrelevant to the gist of the article. |
|  | Goldman RHMDMPH, Stason WBMD, Park SKS, et al. Acupuncture for Treatment of Persistent Arm Pain Due to Repetitive Use: A Randomized Controlled Clinical Trial. Clinical Journal of Pain March/April 2008;24(3):211-18 | Contra to protocol:not a systematic review |
|  | Lee YC, Shmerling RH. The benefit of nonpharmacologic therapy to treat symptomatic osteoarthritis. Current Rheumatology Reports 2008;10(1):5-10 | Contra to protocol: Irrelevant to the gist of the article. |
|  | Gutierrez GPMD. Managing osteoarthritis: What's best for your patient?: New guidelines rate the evidence for hip and knee OA treatments. Handy chart provides at-a-glance summary. Journal of Family Practice 2008;57(10):644-50 | Contra to protocol: included studies involving patients with other chronic diseases and not a systematic review. |
|  | Jamtvedt G, Dahm KT, Holm I, et al. Measuring physiotherapy performance in patients with osteoarthritis of the knee: a prospective study. BMC Health Services Research 2008;8:145 | Contra to protocol:not a systematic review |
|  | Herbal Medicine. Focus on Alternative & Complementary Therapies an Evidence-Based Approach 2008;13(1):49 | Contra to protocol: Irrelevant to the gist of the article. |
|  | Maa S-H, Sun M-F, Wu C-C. The Effectiveness of Acupuncture on Pain and Mobility in Patients With Osteoarthritis of the Knee: A Pilot Study. Journal of Nursing Research 2008;16(2):140-48 | Contra to protocol:not a systematic review |
|  | Pollmann W, Feneberg W. Current Management of Pain Associated with Multiple Sclerosis. CNS Drugs 2008;22(4):291-324 | Contra to protocol:did not focused on knee osteoarthritis |
|  | Gonzalez EBMDFF, Nguyen-Oghalai TMD. Acupuncture of the Knee. Southern Medical Journal 2008;101(2):113 | Contra to protocol:not a systematic review |
|  | Zhang W, Robertson J, Jones AC, et al. The placebo effect and its determinants in osteoarthritis: meta-analysis of randomised controlled trials. Annals of the Rheumatic Diseases 2008;67(12):1716-23 | Contra to protocol:included studies involving patients with other diseases. Diabetes results not reported separately. |
|  | WESTERN REGIONAL MEETING AT A GLANCE. Journal of Investigative Medicine 2008;56(1):103-267 | Contra to protocol: Irrelevant to the gist of the article. |
|  | Perez and Brady's Principles and Practice of Radiation Oncology, 2008. | Contra to protocol: Irrelevant to the gist of the article. |
|  | Silverman SLMDFF. Paget Disease of Bone: Therapeutic Options. JCR: Journal of Clinical Rheumatology 2008;14(5):299-305 | Contra to protocol:did not focused on knee osteoarthritis |
|  | Principles and Practice of Gastrointestinal Oncology, 2008. | Contra to protocol: Irrelevant to the gist of the article. |
|  | Wang C, De Pablo P, Chen X, et al. Acupuncture For Pain Relief in Patients With Rheumatoid Arthritis: A Systematic Review. Arthritis & Rheumatism Arthritis Care & Research 2008;59(9):1249-56 | Contra to protocol:did not focused on knee osteoarthritis |
|  | Hochberg MC. Traditional Chinese medicine in the management of osteoarthritis. Future Rheumatology 2008;3(1):7-9 | Contra to protocol:not a systematic review |
|  | Bodnar RJ. Endogenous opiates and behavior: 2007. Peptides 2008;29(12):2292-375 | Contra to protocol: Irrelevant to the gist of the article. |
|  | Khan NMD, Husain SMD, Haak MMD. Thoracolumbar Injuries in the Athlete. Sports Medicine & Arthroscopy Review 2008;16(1):16-25 | Contra to protocol: Irrelevant to the gist of the article. |
|  | Kim YHMDMPH, Bowers J. Health Benefits of Tai Chi. Alternative Medicine Alert 2008;11(5):53-56 | Contra to protocol:not a systematic review |
|  | Other Complementary Therapies. Focus on Alternative & Complementary Therapies an Evidence-Based Approach 2009;14(4):345 | Contra to protocol:did not focused on knee osteoarthritis |
|  | Needling Questions About Acupuncture for Pain. Lippincott's Bone and Joint Newsletter 2009;15(6):65-66 | Contra to protocol:not a systematic review |
|  | Benjamin M. The fascia of the limbs and back - a review. Journal of Anatomy 2009;214(1):1-18 | Contra to protocol:did not focused on knee osteoarthritis |
|  | Kreitzer MJPRNF, Kligler BMDMPH, Meeker WCDCMPH. Health Professions Education and Integrative Healthcare. Explore: The Journal of Science & Healing July/August 2009;5(4):212-27 | Contra to protocol: Irrelevant to the gist of the article. |
|  | . Acupuncture is cost effective in chronic osteoarthritis pain, particularly in women. Focus on Alternative & Complementary Therapies an Evidence-Based Approach 2009;14(1):33-34 | Contra to protocol:not a systematic review |
|  | Bodnar RJ. Endogenous opiates and behavior: 2008. Peptides 2009;30(12):2432-79 | Contra to protocol：Study on Non-acupuncture or Non-moxibustion interventions |
|  | Marcus DMMD, McCullough LP. An Evaluation of the Evidence in "Evidence-Based" Integrative Medicine Programs. Academic Medicine 2009;84(9):1229-34 | Contra to protocol: Irrelevant to the gist of the article. |
|  | Ahsin Sab, Saleem Sc, Bhatti AMd, et al. Clinical and endocrinological changes after electro-acupuncture treatment in patients with osteoarthritis of the knee. Pain 2009;147(1-2-3):60-66 | Contra to protocol:not a systematic review |
|  | Butoescu N, Jordan O, Doelker E. Intra-articular drug delivery systems for the treatment of rheumatic diseases: A review of the factors influencing their performance. European Journal of Pharmaceutics & Biopharmaceutics 2009;73(2):205-18 | Contra to protocol：Study on Non-acupuncture or Non-moxibustion interventions |
|  | Conaghan PG, Sharma L. Fast Facts: Osteoarthritis, 2009. | Contra to protocol:not a systematic review |
|  | Hagen KB, Smedslund G, Moe RH, et al. The evidence for non-pharmacological therapy of hand and hip OA. Nature Reviews Rheumatology 2009;5(9):517-19 | Contra to protocol:did not focused on knee osteoarthritis |
|  | Colbert AP, Wahbeh H, Harling N, et al. Static Magnetic Field Therapy: A Critical Review of Treatment Parameters. Evidence-Based Complementary & Alternative Medicine: eCAM 2009;6(2):133-39 | Contra to protocol：Study on Non-acupuncture or Non-moxibustion interventions |
|  | Cooper G, Kahn S, Zucker P. Acupuncture for Musculoskeletal Medicine, 2009. | Contra to protocol:not a systematic review |
|  | Tanenbaum SJP. Comparative effectiveness research: evidence-based medicine meets health care reform in the USA. Journal of Evaluation in Clinical Practice 2009;15(6):976-84 | Contra to protocol: Irrelevant to the gist of the article. |
|  | Cox F. Managing pain in osteoarthritis. Primary Health Care 2009;19(7):38-45 | Contra to protocol:not a systematic review |
|  | Stor W, Irnich D. Acupuncture: Basics, practice, and evidence. [German]  Akupunktur : Grundlagen, Praxis und Evidenz. Anaesthesist 2009;58(3):311-24 | Contra to protocol:did not focused on knee osteoarthritis |
|  | Croom KF, Siddiqui MAA. Etoricoxib: A Review of its Use in the Symptomatic Treatment of Osteoarthritis, Rheumatoid Arthritis, Ankylosing Spondylitis and Acute Gouty Arthritis. Drugs 2009;69(11):1513-32 | Contra to protocol：Study on Non-acupuncture or Non-moxibustion interventions |
|  | Cousins & Bridenbaugh's Neural Blockade in Clinical Anesthesia and Pain Medicine, 2009. | Contra to protocol: Irrelevant to the gist of the article. |
|  | Crosby JMD. Osteoarthritis: Managing without surgery: To help patients who want to "try everything" before considering joint replacement, turn to the STEPs approach. Journal of Family Practice 2009;58(7):354-61 | Contra to protocol:not a systematic review |
|  | White Acrf, Cummings Mmd. Does acupuncture relieve pain? BMJ 2009;338(7690):303-04 | Contra to protocol: Irrelevant to the gist of the article. |
|  | Hepper CTMD, Halvorson JJMD, Duncan STMD, et al. The Efficacy and Duration of Intra-articular Corticosteroid Injection for Knee Osteoarthritis: A Systematic Review of Level I Studies. [Review]: Journal of the American Academy of Orthopaedic Surgeons October 2009;17(10):638-646, 2009. | Contra to protocol：Study on Non-acupuncture or Non-moxibustion interventions |
|  | Lee MS, Shin BC, Choi SM, et al. Randomized clinical trials of constitutional acupuncture: a systematic review. Evidence Based Complementary & Alternative Medicine: eCAM 2009;1:59-64 | Contra to protocol:did not focused on knee osteoarthritis |
|  | Hurley MVa, Walsh NEb. Effectiveness and clinical applicability of integrated rehabilitation programs for knee osteoarthritis. Current Opinion in Rheumatology 2009;21(2):171-76 | Contra to protocol:not a systematic review |
|  | Fiechtner J, Dinning D. Non-Pharmacologic Treatment Options in Rheumatologic Disease. Current Rheumatology Reviews 2009;5(4):199-203 | Contra to protocol:did not focused on knee osteoarthritis |
|  | Jones IMBCF, Johnson MIPPoP, Analgesia. Transcutaneous electrical nerve stimulation. Continuing Education in Anaesthesia, Critical Care & Pain 2009;9(4):130-35 | Contra to protocol：Study on Non-acupuncture or Non-moxibustion interventions |
|  | Principles of Addiction Medicine, 2009. | Contra to protocol: Irrelevant to the gist of the article. |
|  | Kelly RB. Acupuncture for pain. American Family Physician 2009;80(5):481-4 | Contra to protocol:not a systematic review |
|  | Anesthesiologist's Manual of Surgical Procedures, 2009. | Contra to protocol: Irrelevant to the gist of the article. |
|  | Lee H-J, Park H-J, Chae Y, et al. Tai Chi Qigong for the quality of life of patients with knee osteoarthritis: a pilot, randomized, waiting list controlled trial. Clinical Rehabilitation 2009;23(6):504-11 | Contra to protocol:not a systematic review |
|  | Nuesch E, Reichenbach S, Trelle S, et al. The importance of allocation concealment and patient blinding in osteoarthritis trials: A meta-epidemiologic study. Arthritis & Rheumatism 2009;61(12):1633-41 | Contra to protocol：Study on Non-acupuncture or Non-moxibustion interventions |
|  | Primary Care Medicine: Office Evaluation and Management of the Adult Patient, 2009. | Contra to protocol: Irrelevant to the gist of the article. |
|  | Fernandez-de-las-Penas Cabc, Schoenen Jd. Chronic tension-type headache: what is new? Current Opinion in Neurology 2009;22(3):254-61 | Contra to protocol:did not focused on knee osteoarthritis |
|  | Lin JG, Chen WL. Review: acupuncture analgesia in clinical trials. American Journal of Chinese Medicine 2009;37(1):1-18 | Contra to protocol:not a systematic review |
|  | Rutjes AW, Nuesch E, Sterchi R, et al. Transcutaneous electrostimulation for osteoarthritis of the knee. Cochrane Database of Systematic Reviews 2009(4):CD002823 | Contra to protocol：Study on Non-acupuncture or Non-moxibustion interventions |
|  | Marcus DMMD, McCullough LP. An Evaluation of the Evidence in "Evidence-Based" Integrative Medicine Programs. [Miscellaneous]: Academic Medicine September 2009;84(9):1229-1234, 2009 | Contra to protocol: Irrelevant to the gist of the article. |
|  | Scott ANP, Guo BMD, Barton PMMD, et al. Trigger Point Injections for Chronic Non-Malignant Musculoskeletal Pain: A Systematic Review. Pain Medicine January/February 2009;10(1):54-69 | Contra to protocol:did not focused on knee osteoarthritis |
|  | White A. NICE guideline on osteoarthritis: is it fair to acupuncture? No. Acupuncture in Medicine 2009;27(2):70-72 | Contra to protocol: Irrelevant to the gist of the article. |
|  | Richmond JMD, Hunter DMDP, Irrgang JPTPATC, et al. Treatment of Osteoarthritis of the Knee (Nonarthroplasty). Journal of the American Academy of Orthopaedic Surgeons 2009;17(9):591-600 | Contra to protocol:not a systematic review |
|  | Cummings M. Why recommend acupuncture for low back pain but not for osteoarthritis? A commentary on recent NICE guidelines. Acupuncture in Medicine 2009;27(3):128-29 | Contra to protocol:did not focused on knee osteoarthritis |
|  | Acupuncture. Focus on Alternative & Complementary Therapies an Evidence-Based Approach 2009;14(1):56 | Contra to protocol: Irrelevant to the gist of the article. |
|  | Rosenthal AK. Osteoarthritis: The Need for Innovative Therapeutic Interventions. Current Rheumatology Reviews 2009;5(1):3-7 | Contra to protocol:not a systematic review |
|  | Stor W, Irnich D. [Acupuncture. Basics, practice, and evidence]. Anaesthesist 2009;58(3):311-23; quiz 24 | Contra to protocol:not a systematic review |
|  | Madsen MVp, Gotzsche PCd, Hrobjartsson Asr. Acupuncture treatment for pain: systematic review of randomised clinical trials with acupuncture, placebo acupuncture, and no acupuncture groups. BMJ 2009;338(7690):330-37 | Contra to protocol:did not focused on knee osteoarthritis |
|  | Summaries and commentaries by editor Adrian White on a selection of recent acupuncture research articles. Acupuncture in Medicine 2009;27(2):86-89 | Contra to protocol: Irrelevant to the gist of the article. |
|  | Taechaarpornkul W, Suvapan D, Theppanom C, et al. Comparison of the effectiveness of six and two acupuncture point regimens in osteoarthritis of the knee: a randomised trial. Acupuncture in Medicine 2009;27(1):3-8 | Contra to protocol:not a systematic review |
|  | McNair PJ, Simmonds MA, Boocock MG, et al. Exercise therapy for the management of osteoarthritis of the hip joint: A systematic review. Arthritis Research and Therapy 2009;11 (3) (no pagination)(R98) | Contra to protocol:did not focused on knee osteoarthritis |
|  | Thomas A, Eichenberger G, Kempton C, et al. Recommendations for the treatment of knee osteoarthritis, using various therapy techniques, based on categorizations of a literature review. Journal of Geriatric Physical Therapy 2009;32(1):33-38 | Contra to protocol:not a systematic review |
|  | Chung W, Xu S, Eken A, et al. Current Status of Complementary and Alternative Medicine in the Treatment of Rheumatic Disease Pain. Current Rheumatology Reviews 2009;5(4):194-98 | Contra to protocol:did not focused on knee osteoarthritis |
|  | Thomas A, Eichenberger G, Kempton C, et al. Recommendations for the Treatment of Knee Osteoarthritis, Using Various Therapy Techniques, Based on Categorizations of a Literature Review. Journal of Geriatric Physical Therapy 2009;32(1):33-38 | Contra to protocol:not a systematic review |
|  | Abstracts of the XXVIII Annual European Society of Regional Anaesthesia Congress, Salzburg, Austria, September 9-12, 2009. Regional Anesthesia & Pain Medicine September/October 2009;34(5):1-192 | Contra to protocol: Irrelevant to the gist of the article. |
|  | Celiker R. Current treatment approaches for osteoarthritis in the elderly. [Turkish]  Yaslilarda osteoartrit tedavisine guncel yaklasimlar. Turkiye Fiziksel Tip ve Rehabilitasyon Dergisi 2009;55(SUPP.2):75-79 | Contra to protocol: included studies involving patients with other chronic diseases and not a systematic review. |
|  | Molsberger AMD. Commentary on the Cochrane Review of Acupuncture for Tension-Type Headache. Explore: The Journal of Science & Healing November/December 2009;5(6):356-58 | Contra to protocol:did not focused on knee osteoarthritis |
|  | Thompson JM, Chiasson R, Loisel P, et al. A sailor's pain: Veterans' musculoskeletal disorders, chronic pain, and disability. Canadian Family Physician 2009;55(11):1085-8 | Contra to protocol:not a systematic review |
|  | Nuesch E, Reichenbach S, Trelle S, et al. The importance of allocation concealment and patient blinding in osteoarthritis trials: A meta-epidemiologic study. Arthritis Care and Research 2009;61(12):1633-41 | Contra to protocol: included studies involving patients with other chronic diseases and not a systematic review. |
|  | Marx RCMD, Mizel MSMD. What's New in Foot and Ankle Surgery. Journal of Bone & Joint Surgery - American Volume 2009;91(4):1023-31 | Contra to protocol:did not focused on knee osteoarthritis |
|  | La douleur d'un marin: Troubles musculosquelettiques, douleur chronique et invalidite chez les veterans militaires. Canadian Family Physician 2009;55(11):1085-88+e50-e54 | Contra to protocol:not a systematic review |
|  | Summaries and commentaries by editor Adrian White on a selection of recent acupuncture research studies. Acupuncture in Medicine 2009;27(4):191-94 | Contra to protocol: Irrelevant to the gist of the article. |
|  | Chan DKCM, Johnson MIP, Sun KOMFFF, et al. Electrical Acustimulation of the Wrist for Chronic Neck Pain: A Randomized, Sham-controlled Trial Using a Wrist-Ankle Acustimulation Device. Clinical Journal of Pain 2009;25(4):320-26 | Contra to protocol:did not focused on knee osteoarthritis |
|  | Abstracts of the XXVIII Annual European Society of Regional Anaesthesia Congress, Salzburg, Austria, September 9-12, 2009. [Miscellaneous]: Regional Anesthesia & Pain Medicine September/October 2009;34(5):1-192, 2009. | Contra to protocol: Irrelevant to the gist of the article. |
|  | Seed SM, Dunican KC, Lynch AM. Osteoarthritis: A review of treatment options. Geriatrics 2009;64(10):20-29 | Contra to protocol:included studies involving patients with other diseases. Interventions are not acupuncture and moxibustion. |
|  | Wagner E. [Non-surgical treatment of osteoarthritis of large joints - new aspects]. Wiener Medizinische Wochenschrift 2009;159(3-4):76-86 | Contra to protocol:not a systematic review |
|  | Zhang W, Nuki G, Moskowitz RW, et al. OARSI recommendations for the management of hip and knee osteoarthritis. Part III: Changes in evidence following systematic cumulative update of research published through January 2009. Osteoarthritis and Cartilage 2010;18(4):476-99 | Contra to protocol：Study on Non-acupuncture or Non-moxibustion interventions |
|  | Tosi LLM, Maher NP, Moore WDB, et al. Adults with cerebral palsy: a workshop to define the challenges of treating and preventing secondary musculoskeletal and neuromuscular complications in this rapidly growing population. Developmental Medicine & Child Neurology 2009;51 Supplement(4):2-11 | Contra to protocol:did not focused on knee osteoarthritis |
|  | Ahsin Sab, Saleem Sc, Bhatti AMd, et al. Clinical and endocrinological changes after electro-acupuncture treatment in patients with osteoarthritis of the knee. [Article]: Pain December 2009;147(1-2-3):60-66, 2009 | Contra to protocol:not a systematic review |
|  | Shin SYMSRN, Kolanowski AMPRNF. Best Evidence of Psychosocially Focused Nonpharmacologic Therapies for Symptom Management in Older Adults with Osteoarthritis. Pain Management Nursing 2010;11(4):234-44 | Contra to protocol：Study on Non-acupuncture or Non-moxibustion interventions |
|  | McCarron JA. Shoulder arthritis and the young patient. Current Orthopaedic Practice 2009;20(4):382-87 | Contra to protocol:did not focused on knee osteoarthritis |
|  | Richmond JMD, Hunter DMDP, Irrgang JPTPATC, et al. Treatment of Osteoarthritis of the Knee (Nonarthroplasty). [Miscellaneous Article]: Journal of the American Academy of Orthopaedic Surgeons September 2009;17(9):591-600, 2009 | Contra to protocol:not a systematic review |
|  | DeSantana JMa, da Silva LFSb, Sluka KAb. Cholecystokinin receptors mediate tolerance to the analgesic effect of TENS in arthritic rats. [Article]: Pain January 2010;148(1):84-93, 2010. | Contra to protocol:did not focused on knee osteoarthritis |
|  | Acupuncture. Focus on Alternative & Complementary Therapies an Evidence-Based Approach 2010;15(2):163-69 | Contra to protocol: Irrelevant to the gist of the article. |
|  | Fulop AMDPT, Dhimmer SDPT, Deluca JRDPT, et al. A Meta-analysis of the Efficacy of Laser Phototherapy on Pain Relief. Clinical Journal of Pain 2010;26(8):729-36 | Contra to protocol：Study on Non-acupuncture or Non-moxibustion interventions |
|  | Finckh A, Tramer MR. Small studies overestimate the benefit of therapies for OA. Nature Reviews Rheumatology 2010;6(11):617-18 | Contra to protocol:not a systematic review |
|  | Abstracts of the XXIX Annual European Society of Regional Anaesthesia (ESRA) Congress 2010. [Miscellaneous]: Regional Anesthesia & Pain Medicine September/October 2010;35(5):E1-E195, 2010. | Contra to protocol: Irrelevant to the gist of the article. |
|  | Rannou F, Poiraudeau S. Non-pharmacological approaches for the treatment of osteoarthritis. Best Practice and Research: Clinical Rheumatology 2010;24(1):93-106 | Contra to protocol:not a systematic review |
|  | Bruyre O, Reginster JY, Croisier JL, et al. Rehabilitation in osteoarthritis. Therapy 2010;7(6):669-74 | Contra to protocol: included studies involving patients with other chronic diseases and not a systematic review. |
|  | Cherniack PE. Would the elderly be better off if they were given more placebos? Geriatrics & Gerontology International 2010;10(2):131-37 | Contra to protocol: Irrelevant to the gist of the article. |
|  | Forestier R, Desfour H, Tessier JM, et al. Spa therapy in the treatment of knee osteoarthritis: a large randomised multicentre trial. Annals of the Rheumatic Diseases 2010;69(4):660-65 | Contra to protocol：Study on Non-acupuncture or Non-moxibustion interventions |
|  | Bruyere O, Reginster J-Y, Croisier J-L, et al. Rehabilitation in osteoarthritis. Therapy 2010;7(6):669-74 | Contra to protocol:not a systematic review |
|  | Bennell Kp, Wee Ep, research a, et al. Efficacy of standardised manual therapy and home exercise programme for chronic rotator cuff disease: randomised placebo controlled trial. BMJ June 2010;12(340) | Contra to protocol:did not focused on knee osteoarthritis |
|  | Brown C, Jones A. A response to O'Connell et al. letter "A failure of the review process? Comment on Ahsin et al. Clinical and endocrinological changes after electro-acupuncture treatment in patients with osteoarthritis of the knee. Pain 2009;147: 60-6". Pain 2010;149(1):161 | Contra to protocol: Irrelevant to the gist of the article. |
|  | Brown C, Jones A. A response to O'Connell et al. letter "A failure of the review process? Comment on Ahsin et al. Clinical and endocrinological changes after electro-acupuncture treatment in patients with osteoarthritis of the knee. Pain 2009;147: 60-6". [Letter]: Pain April 2010;149(1):161, 2010. | Contra to protocol:not a systematic review |
|  | Washington Manual of Outpatient Internal Medicine, The, 2010. | Contra to protocol: Irrelevant to the gist of the article. |
|  | Henrotin Y, Chevalier X. Guidelines for the management of knee and hip osteoarthritis: For Whom? Why? To do what?. [French]  ecommandations sur la prise en charge de l'arthrose de la hanche et du genou. Pour qui ? Pourquoi ? Pour quoi faire ? Presse Medicale 2010;39(11):1180-88 | Contra to protocol: included studies involving patients with other chronic diseases and not a systematic review. |
|  | Fazzino DLDNPRNFNPBCC, Griffin MTQPRN, McNulty SRDNPRNA-BC, et al. Energy Healing and Pain: A Review of the Literature. Holistic Nursing Practice March/April 2010;24(2):79-88 | Contra to protocol: Irrelevant to the gist of the article. |
|  | DeSantana JMa, da Silva LFSb, Sluka KAb. Cholecystokinin receptors mediate tolerance to the analgesic effect of TENS in arthritic rats. Pain 2010;148(1):84-93 | Contra to protocol：Study on Non-acupuncture or Non-moxibustion interventions |
|  | Capili BDNPC, Anastasi JKPDNPFLA, Geiger JNMSFNP. Adverse Event Reporting in Acupuncture Clinical Trials Focusing on Pain. Clinical Journal of Pain 2010;26(1):43-48 | Contra to protocol:did not focused on knee osteoarthritis |
|  | Bodnar RJ. Endogenous opiates and behavior: 2009. Peptides 2010;31(12):2325-59 | Contra to protocol: Irrelevant to the gist of the article. |
|  | Capili BDNPC, Anastasi JKPDNPFLA, Geiger JNMSFNP. Adverse Event Reporting in Acupuncture Clinical Trials Focusing on Pain. [Article]: Clinical Journal of Pain January 2010;26(1):43-48, 2010. | Contra to protocol:not a systematic review |
|  | Merritt's Neurology, 2010. | Contra to protocol: Irrelevant to the gist of the article. |
|  | Chaudhury S, Gwilym SE, Moser J, et al. Surgical options for patients with shoulder pain. Nature Reviews Rheumatology 2010;6(4):217-26 | Contra to protocol:did not focused on knee osteoarthritis |
|  | March L, Amatya B, Osborne RH, et al. Developing a minimum standard of care for treating people with osteoarthritis of the hip and knee. Best Practice and Research: Clinical Rheumatology 2010;24(1):121-45 | Contra to protocol: included studies involving patients with other chronic diseases and not a systematic review. |
|  | Bonica's Management of Pain, 2010. | Contra to protocol: Irrelevant to the gist of the article. |
|  | O'Connell NE, Wand BM, Colquhoun D. A failure of the review process? Comment on Ahsin et al. Clinical and endocrinological changes after electro-acupuncture treatment in patients with osteoarthritis of the knee. Pain 2009;147:60-6. Pain 2010;149(1):160 | Contra to protocol:not a systematic review |
|  | Castillo-Bueno MDR, Moreno-Pina JPRNM, Martinez-Puente MVRNB, et al. Effectiveness of nursing intervention for adult patients experiencing chronic pain: a systematic review. JBI Library of Systematic Reviews 2010;8(28):1112-68 | Contra to protocol：Study on Non-acupuncture or Non-moxibustion interventions |
|  | Delisa's Physical Medicine & Rehabilitation: Principles And Practice, 2010. | Contra to protocol: Irrelevant to the gist of the article. |
|  | D. I. Summaries and commentaries by Adrian White on a selection of recent acupuncture research studies. Acupuncture in Medicine 2010;28(1):52-55 | Contra to protocol:not a systematic review |
|  | Other Complementary Therapies. Focus on Alternative & Complementary Therapies an Evidence-Based Approach 2010;15(1):63-68 | Contra to protocol: Irrelevant to the gist of the article. |
|  | Izquierdo RMD, Voloshin IMD, Edwards SMD, et al. Treatment of Glenohumeral Osteoarthritis. Journal of the American Academy of Orthopaedic Surgeons 2010;18(6):375-82  La Touche RMPT, Goddard GDDS, De-la-Hoz JLMDDDS, et al. Acupuncture in the Treatment of Pain in Temporomandibular Disorders: A Systematic Review and Meta-analysis of Randomized Controlled Trials. Clinical Journal of Pain July/August 2010;26(6):541-50 | Contra to protocol:did not focused on knee osteoarthritis |
|  | Summaries and commentaries by Adrian White on a selection of recent acupuncture studies. Acupuncture in Medicine 2010;28(4):207-10 | Contra to protocol: Irrelevant to the gist of the article. |
|  | Uhlig T, Slatkowsky-Christensen B, Moe RH, et al. The burden of osteoarthritis:the societal and the patient perspective. Therapy 2010;7(6):605-19 | Contra to protocol:not a systematic review |
|  | RECENT LITERATURE. Focus on Alternative & Complementary Therapies an Evidence-Based Approach 2010;15(4):324-33 | Contra to protocol: Irrelevant to the gist of the article. |
|  | Fazzino DLDNPRNFNPBCC, Griffin MTQPRN, McNulty SRDNPRNA-BC, et al. Energy Healing and Pain: A Review of the Literature. [Article]: Holistic Nursing Practice March/April 2010;24(2):79-88, 2010. | Contra to protocol:not a systematic review |
|  | Toupin-April K, Hochberg M, Tugwell P, et al. Development of the 2009 revised ACR recommendations for the management of osteoarthritis. Journal of Rheumatology 2010;2):1336 | Contra to protocol:included studies involving patients with other diseases. Interventions are not acupuncture and moxibustion. |
|  | Whitehead ML. Use of acupuncture in veterinary medicine. Veterinary Record 2010;167(26):1018 | Contra to protocol: Irrelevant to the gist of the article. |
|  | Fraenkel L. Unpacking the effects of acupuncture. Arthritis Care and Research 2010;62(9):1203-05 | Contra to protocol:not a systematic review |
|  | Abstracts of the XXIX Annual European Society of Regional Anaesthesia (ESRA) Congress 2010. Regional Anesthesia & Pain Medicine September/October 2010;35(5):E1-E195 | Contra to protocol: Irrelevant to the gist of the article. |
|  | Li HY, Cui L, Cui M, et al. Active research fields of acupuncture research: a document co-citation clustering analysis of acupuncture literature. Alternative Therapies in Health & Medicine 2010;16(6):38-45 | Contra to protocol:did not focused on knee osteoarthritis |
|  | Accessing FastTrack articles - online ahead of print. Annals of the Royal College of Surgeons of England 2010;92(5):432 | Contra to protocol: Irrelevant to the gist of the article. |
|  | Beadle C, Howie CR, Nuki G. OARSI recommendations for the management of hip and knee osteoarthritis: Which treatments are being used? Audit of patients coming to arthroplasty in Scotland. Osteoarthritis and Cartilage 2010;18:S152 | Contra to protocol：Study on Non-acupuncture or Non-moxibustion interventions |
|  | Nuesch E, Trelle S, Reichenbach S, et al. Small study effects in meta-analyses of osteoarthritis trials: Meta-epidemiological study. BMJ (Online) 2010;341(7766):241 | Contra to protocol:included studies involving patients with other diseases. Interventions are not acupuncture and moxibustion. |
|  | Pelletier KRPMD, Herman PMNDP, Metz DRDC, et al. Health and Medical Economics Applied to Integrative Medicine. Explore: The Journal of Science & Healing March/April 2010;6(2):86-99 | Contra to protocol: Irrelevant to the gist of the article. |
|  | Langworthy MJ, Saad A, Langworthy NM. Conservative treatment modalities and outcomes for osteoarthritis: the concomitant pyramid of treatment. Physician & Sportsmedicine 2010;38(2):133-45 | Contra to protocol:not a systematic review |
|  | Nuesch Erf, Trelle Sad, Reichenbach Ssrf, et al. Small study effects in meta-analyses of osteoarthritis trials: meta-epidemiological study. BMJ July 2010;17(341) | Contra to protocol :included studies involving patients with other chronic diseases and Meta analysis article |
|  | Fregni FMDP, Imamura MMDP, Chien HFMDP, et al. Challenges and Recommendations for Placebo Controls in Randomized Trials in Physical and Rehabilitation Medicine: A Report of the International Placebo Symposium Working Group. [Review]: American Journal of Physical Medicine & Rehabilitation February 2010;89(2):160-172, 2010 | Contra to protocol: Irrelevant to the gist of the article. |
|  | Johnson MI, Benham AE. Acupuncture needle sensation: the emerging evidence. Acupuncture in Medicine 2010;28(3):111-14 | Contra to protocol: Irrelevant to the gist of the article. |
|  | Baird CLDNSCNS, Murawski MMRP, Wu JMS. Efficacy of Guided Imagery with Relaxation for Osteoarthritis Symptoms and Medication Intake. Pain Management Nursing 2010;11(1):56-65 | Contra to protocol：Study on Non-acupuncture or Non-moxibustion interventions |
|  | Mao JJ, Kapur R. Acupuncture in primary care. Primary Care; Clinics in Office Practice 2010;37(1):105-17 | Contra to protocol:not a systematic review |
|  | Wong M. Pocket Orthopaedics: Evidence-Based Survival Guide, 2010. | Contra to protocol: Irrelevant to the gist of the article. |
|  | Marini IMDDDS, Gatto MRP, Bonetti GAMDDDS. Effects of Superpulsed Low-level Laser Therapy on Temporomandibular Joint Pain. Clinical Journal of Pain 2010;26(7):611-16 | Contra to protocol:did not focused on knee osteoarthritis |
|  | Fulop AMDPT, Dhimmer SDPT, Deluca JRDPT, et al. A Meta-analysis of the Efficacy of Laser Phototherapy on Pain Relief. [Article]: Clinical Journal of Pain October 2010;26(8):729-736, 2010. | Contra to protocol: Irrelevant to the gist of the article. |
|  | Foster NE, Thomas E, Hill JC, et al. The relationship between patient and practitioner expectations and preferences and clinical outcomes in a trial of exercise and acupuncture for knee osteoarthritis. European Journal of Pain 2010;14(4):402-09 | Contra to protocol:not a systematic review |
|  | Zhang W, Nuki G, Moskowitz RW, et al. OARSI recommendations for the management of hip and knee osteoarthritis: part III: Changes in evidence following systematic cumulative update of research published through January 2009. Osteoarthritis & Cartilage 2010;18(4):476-99 | Contra to protocol: Irrelevant to the gist of the article. |
|  | Molsberger AFa, Schneider Tb, Gotthardt Hc, et al. German Randomized Acupuncture Trial for chronic shoulder pain (GRASP) - A pragmatic, controlled, patient-blinded, multi-centre trial in an outpatient care environment. Pain 2010;151(1):146-54 | Contra to protocol:did not focused on knee osteoarthritis |
|  | Nunez M, Nunez E, Lozano L, et al. Quality of life after joint replacement for osteoarthritis. Aging Health 2010;6(4):481-94 | Contra to protocol: Irrelevant to the gist of the article. |
|  | Jamtvedt G, Dahm KT, Holm I, et al. Choice of treatment modalities was not influenced by pain, severity or co-morbidity in patients with knee osteoarthritis. Physiotherapy Research International 2010;15(1):16-23 | Contra to protocol:not a systematic review |
|  | Fregni FMDP, Imamura MMDP, Chien HFMDP, et al. Challenges and Recommendations for Placebo Controls in Randomized Trials in Physical and Rehabilitation Medicine: A Report of the International Placebo Symposium Working Group. American Journal of Physical Medicine & Rehabilitation 2010;89(2):160-72 | Contra to protocol: Irrelevant to the gist of the article. |
|  | Foster NED. Commentary on the Cochrane Review of Acupuncture for Peripheral Joint Osteoarthritis. Explore: The Journal of Science & Healing May/June 2010;6(3):189-91 | Contra to protocol:not a systematic review |
|  | Li JH, Su YS, Jing XH, et al. [Analysis and strategy report on overseas large-scale systematic evaluation on clinical effectiveness of acupuncture]. [Chinese]. Zhongguo zhen jiu = Chinese acupuncture & moxibustion 2011;31(7):665-69 | Contra to protocol: Original from Chinese database |
|  | Bjordal JM, Demmink JH. Do systematic reviews of physical agents and pharmacological agents in the Cochrane library interpret scientific evidence differently? Physiotherapy (United Kingdom) 2011;97:eS135 | Contra to protocol:did not focused on knee osteoarthritis |
|  | Hsu ESMD. Acute and Chronic Pain Management in Fibromyalgia: Updates on Pharmacotherapy. [Review]: American Journal of Therapeutics November 2011;18(6):487-509, 2011. | Contra to protocol:not a systematic review |
|  | Cao Y, Zhan H, Pang J, et al. Individually integrated traditional Chinese medicine approach in the management of knee osteoarthritis: study protocol for a randomized controlled trial. Trials [Electronic Resource] 2011;12:160 | Contra to protocol：included studies of protocol |
|  | deWeber KMDF, Lynch JHMDMS. Sideline Acupuncture for Acute Pain Control: A Case Series. Current Sports Medicine Reports November/December 2011;10(6):320-23 | Contra to protocol:not a systematic review |
|  | Abstracts of the 30th Annual European Society of Regional Anaesthesia (ESRA) Congress 2011. Regional Anesthesia & Pain Medicine September/October 2011;36(5):E1-E279 | Contra to protocol: Irrelevant to the gist of the article. |
|  | Johnson MI, Bjordal JM. Transcutaneous electrical nerve stimulation for the management of painful conditions: focus on neuropathic pain. Expert Review of Neurotherapeutics 2011;11(5):735-53 | Contra to protocol：Study on Non-acupuncture or Non-moxibustion interventions |
|  | Li JH, Su YS, Jing XH, et al. [Analysis and strategy report on overseas large-scale systematic evaluation on clinical effectiveness of acupuncture]. Zhongguo Zhenjiu 2011;31(7):665-9 | Contra to protocol:did not focused on knee osteoarthritis |
|  | Mukaimi AA, Salawi EE, Lindstrand A. Osteoarthritis of the knee: Review of risk factors and treatment programs with special reference to evidence-based research. Kuwait Medical Journal 2011;43(3):176-88 | Contra to protocol:not a systematic review |
|  | O'Mathuna DPP. Glucosamine Sulfate for Osteoarthritis. Alternative Medicine Alert 2011;14(2):13-16 | Contra to protocol：Study on Non-acupuncture or Non-moxibustion interventions |
|  | Yuelong C, Hongsheng Z, Jian P, et al. Individually integrated traditional chinese medicine approach in the management of knee osteoarthritis: Study protocol for a randomized controlled trial. Trials 2011;12 (no pagination)(160) | Contra to protocol：included studies of protocol |
|  | Claydon LSBP, Chesterton LSPBBA, Barlas PPB, et al. Dose-specific Effects of Transcutaneous Electrical Nerve Stimulation (TENS) on Experimental Pain: A Systematic Review. [Review]: Clinical Journal of Pain September 2011;27(7):635-647, 2011 | Contra to protocol：Study on Non-acupuncture or Non-moxibustion interventions |
|  | Hunter DJ. Lower extremity osteoarthritis management needs a paradigm shift. British Journal of Sports Medicine 2011;45(4):283-88 | Contra to protocol:not a systematic review |
|  | Davies PSMSA. New Developments in the Treatment of Osteoarthritis: A Focus on Women. Pain Management Nursing 2011;12 Supplement(1):S17-S22 | Contra to protocol:not a systematic review |
|  | RECENT LITERATURE. Focus on Alternative & Complementary Therapies an Evidence-Based Approach 2011;16(3):e1-e6 | Contra to protocol: Irrelevant to the gist of the article. |
|  | Iannitti T, Lodi D, Palmieri B. Intra-Articular Injections for the Treatment of Osteoarthritis: Focus on the Clinical Use of Hyaluronic Acid. Drugs in R & D (Open Access) 2011;11(1):13-27 | Contra to protocol：Study on Non-acupuncture or Non-moxibustion interventions |
|  | McCarberg BHMD, Ruoff GEMD, Tenzer-Iglesias PMD, et al. Diagnosis and Treatment of Low-Back Pain Because of Paraspinous Muscle Spasm: A Physician Roundtable. Pain Medicine 2011;12 Supplement(4):S119-S27 | Contra to protocol:did not focused on knee osteoarthritis |
|  | Oral A, Ilieva E. Physiatric approaches to pain management in osteoarthritis: a review of the evidence of effectiveness. Pain Management 2011;1(5):451-71 | Contra to protocol:not a systematic review |
|  | Yamada EMD, Thomas DCMDMS. Common Musculoskeletal Diagnoses of Upper and Lower Extremities in Older Patients. Mount Sinai Journal of Medicine: A Journal of Personalized & Translational Medicine July/August 2011;78(4):546-57 | Contra to protocol：Study on Non-acupuncture or Non-moxibustion interventions |
|  | Pain Medicine Pocketpedia, 2011. | Contra to protocol: Irrelevant to the gist of the article. |
|  | Textbook of Spinal Surgery, The, 2011. | Contra to protocol: Irrelevant to the gist of the article. |
|  | Backer MMD, Ludtke RM, Afra DMD, et al. Effectiveness of Leech Therapy in Chronic Lateral Epicondylitis: A Randomized Controlled Trial. Clinical Journal of Pain 2011;27(5):442-47 | Contra to protocol:not a systematic review |
|  | Hsu ESMD. Acute and Chronic Pain Management in Fibromyalgia: Updates on Pharmacotherapy. American Journal of Therapeutics 2011;18(6):487-509 | Contra to protocol：Study on Non-acupuncture or Non-moxibustion interventions |
|  | Juhakoski R, Tenhonen S, Kiviniemi It V, et al. A pragmatic randomized controlled study of the effectiveness and cost consequences of exercise therapy in hip osteoarthritis. Clinical Rehabilitation 2011;25(4):370-83 | Contra to protocol:did not focused on knee osteoarthritis |
|  | 5-Minute Sports Medicine Consult, The, 2011 | Contra to protocol: Irrelevant to the gist of the article. |
|  | Witt CMMDMBA, Schutzler LM, Ludtke RM, et al. Patient Characteristics and Variation in Treatment Outcomes: Which Patients Benefit Most From Acupuncture for Chronic Pain? Clinical Journal of Pain July/August 2011;27(6):550-55 | Contra to protocol:not a systematic review |
|  | Hawker GA, Mian S, Bednis K, et al. Osteoarthritis year 2010 in review: non-pharmacologic therapy. Osteoarthritis & Cartilage 2011;19(4):366-74 | Contra to protocol：Study on Non-acupuncture or Non-moxibustion interventions |
|  | Clinical Gynecologic Endocrinology and Infertility, 2011. | Contra to protocol: Irrelevant to the gist of the article. |
|  | Paice JA. Chronic treatment-related pain in cancer survivors. [Review]: Pain March 2011;152(3) Supplement:S84-S89, 2011 | Contra to protocol:did not focused on knee osteoarthritis |
|  | Woolf CJ. Central sensitization: Implications for the diagnosis and treatment of pain. [Review]: Pain March 2011;152(3) Supplement:S2-S15, 2011. | Contra to protocol:not a systematic review |
|  | Alper BSMDM, Glickman-Simon RMD. Tai chi, glucosamine, probiotics, acupressure, and pelargonium sidoides. Explore: The Journal of Science & Healing November/December 2011;7(6):392-95 | Contra to protocol: Irrelevant to the gist of the article. |
|  | De Silva V, El-Metwally A, Ernst E, et al. Evidence for the efficacy of complementary and alternative medicines in the management of osteoarthritis: a systematic review. Rheumatology 2011;50(5):911-20 | Contra to protocol：Study on Non-acupuncture or Non-moxibustion interventions |
|  | Bratton RL. Bratton's Family Medicine Board Review, 2011. | Contra to protocol: Irrelevant to the gist of the article. |
|  | Ross SM. Osteoarthritis of the knee: an integrative therapies approach. Holistic Nursing Practice 2011;25(6):327-31 | Contra to protocol:not a systematic review |
|  | Berger A, Bozic K, Stacey B, et al. Patterns of pharmacotherapy and health care utilization and costs prior to total hip or total knee replacement in patients with osteoarthritis. Arthritis & Rheumatism 2011;63(8):2268-75 | Contra to protocol: included studies involving patients with other chronic diseases and not a systematic review. |
|  | Hollifield M. Acupuncture for Posttraumatic Stress Disorder: Conceptual, Clinical, and Biological Data Support Further Research. CNS: Neuroscience & Therapeutics 2011;17(6):769-79 | Contra to protocol:did not focused on knee osteoarthritis |
|  | Bennell KLp, Hunter DJp, Hinman RSap. Management of osteoarthritis of the knee. BMJ August 2012;4(345) | Contra to protocol:not a systematic review |
|  | Ru TZBSNBS, Associate APEPWHPcMPCBADNNRN, Hegney DGPBADNECCRNRM. A qualitative systematic review on the experiences of self-management in community-dwelling older women living with chronic illnesses. JBI Library of Systematic Reviews 2011;9(62):2778-828 | Contra to protocol：Study on Non-acupuncture or Non-moxibustion interventions |
|  | Coronado RAPCF, Wurtzel WA, Simon CBPDF, et al. Content and Bibliometric Analysis of Articles Published in the Journal of Orthopaedic & Sports Physical Therapy. Journal of Orthopaedic & Sports Physical Therapy 2011;41(12):920-31 | Contra to protocol: Irrelevant to the gist of the article. |
|  | Spicka C, Fenlon D, Adams J. A review of the literature for non-pharmacological interventions for arthralgia in non-cancer conditions. European Journal of Cancer 2011;47:S317 | Contra to protocol:not a systematic review |
|  | Wallis JA, Taylor NF. Pre-operative interventions (non-surgical and non-pharmacological) for patients with hip or knee osteoarthritis awaiting joint replacement surgery - a systematic review and meta-analysis. Osteoarthritis and Cartilage 2011;19(12):1381-95 | Contra to protocol：Study on Non-acupuncture or Non-moxibustion interventions |
|  | deWeber KMDF, Lynch JHMDMS. Sideline Acupuncture for Acute Pain Control: A Case Series. [Report]: Current Sports Medicine Reports November/December 2011;10(6):320-323, 2011. | Contra to protocol: Irrelevant to the gist of the article. |
|  | Costantino C, Kwarecki J, Samokhin AV, et al. Diclofenac Epolamine plus Heparin Plaster versus Diclofenac Epolamine Plaster in Mild to Moderate Ankle Sprain: A Randomized, Double-Blind, Parallel-Group, Placebo-Controlled, Multicentre, Phase III Trial. Clinical Drug Investigation 2011;31(1):15-26 | Contra to protocol：Study on Non-acupuncture or Non-moxibustion interventions |
|  | Choi TY, Kim TH, Kang JW, et al. Moxibustion for rheumatic conditions: a systematic review and meta-analysis. Clinical Rheumatology 2011;30(7):937-45 | Contra to protocol:did not focused on knee osteoarthritis |
|  | Hadjistavropoulos T, Craig KD, Duck S, et al. A Biopsychosocial Formulation of Pain Communication. Psychological Bulletin 2011;137(6):910-39 | Contra to protocol: Irrelevant to the gist of the article. |
|  | Han J-S. Acupuncture analgesia: Areas of consensus and controversy. Pain 2011;152(3)(Supplement):S41-S48 | Contra to protocol: Irrelevant to the gist of the article. |
|  | Baraf HSB, Gloth FM, Barthel HR, et al. Safety and Efficacy of Topical Diclofenac Sodium Gel for Knee Osteoarthritis in Elderly and Younger Patients: Pooled Data from Three Randomized, Double-Blind, Parallel-Group, Placebo-Controlled, Multicentre Trials. Drugs & Aging 2011;28(1):27-40 | Contra to protocol：Study on Non-acupuncture or Non-moxibustion interventions |
|  | Israel HFNPP, Richter RRPTP. A Guide to Understanding Meta-Analysis. Journal of Orthopaedic & Sports Physical Therapy 2011;41(7):496-504 | Contra to protocol: Irrelevant to the gist of the article. |
|  | Jablonski AMPRN, DuPen ARMNAA, Ersek MPRNF. The Use of Algorithms in Assessing and Managing Persistent Pain in Older Adults. [Article]: AJN, American Journal of Nursing March 2011;111(3):34-43, 2011. | Contra to protocol: Irrelevant to the gist of the article. |
|  | Kienle GSMD, Albonico H-UMD, Fischer LMD, et al. Complementary Therapy Systems and Their Integrative Evaluation. Explore: The Journal of Science & Healing 2011;7(3):175-87 | Contra to protocol: Irrelevant to the gist of the article. |
|  | Claydon LSBP, Chesterton LSPBBA, Barlas PPB, et al. Dose-specific Effects of Transcutaneous Electrical Nerve Stimulation (TENS) on Experimental Pain: A Systematic Review. Clinical Journal of Pain 2011;27(7):635-47 | Contra to protocol：Study on Non-acupuncture or Non-moxibustion interventions |
|  | White A. Recent papers summarised by Adrian White. Acupuncture in Medicine 2011;29(3):234-39 | Contra to protocol: Irrelevant to the gist of the article. |
|  | Acharya SS. Exploration of the pathogenesis of haemophilic joint arthropathy: understanding implications for optimal clinical management. British Journal of Haematology 2012;156(1):13-23 | Contra to protocol:did not focused on knee osteoarthritis |
|  | Fouladbakhsh J. Complementary and Alternative Modalities to Relieve Osteoarthritis Symptoms: A review of the evidence on several therapies often used for osteoarthritis management. [Miscellaneous Article]: Orthopaedic Nursing March/April 2012;31(2):115-121, 2012. | Contra to protocol: included studies involving patients with other chronic diseases and not a systematic review. |
|  | A W. Exploring the evidence for using TENS to relieve pain. Nursing Times 2012;108(11):20-23 | Contra to protocol：Study on Non-acupuncture or Non-moxibustion interventions |
|  | Collins NJ, Bisset LM, Crossley KM, et al. Efficacy of Nonsurgical Interventions for Anterior Knee Pain: Systematic Review and Meta-Analysis of Randomized Trials. Sports Medicine 2012;42(1):31-49 | Contra to protocol:did not focused on knee osteoarthritis |
|  | Glickman-Simon RMD, Ehrlich AMD. Leeches, Creatine, Xylitol, Spinal Manipulation, Acupuncture. Explore: The Journal of Science & Healing 2012;8(3):206-09 | Contra to protocol:did not focused on knee osteoarthritis |
|  | Iversen MD. Rehabilitation Interventions for Pain and Disability in Osteoarthritis: A review of interventions including exercise, manual techniques, and assistive devices. Orthopaedic Nursing March/April 2012;31(2):103-08 | Contra to protocol:not a systematic review |
|  | Roos EM, Juhl CB. Osteoarthritis 2012 year in review: Rehabilitation and outcomes. Osteoarthritis and Cartilage 2012;20(12):1477-83 | Contra to protocol: included studies involving patients with other chronic diseases and not a systematic review. |
|  | Mavrommatis CIa, Argyra Eb, Vadalouka Ab, et al. Acupuncture as an adjunctive therapy to pharmacological treatment in patients with chronic pain due to osteoarthritis of the knee: A 3-armed, randomized, placebo-controlled trial. Pain 2012;153(8):1720-26 | Contra to protocol:not a systematic review |
|  | Balachandar V, Barton C, Morrissey D. THE EFFICACY OF PATELLAR TAPING IN INDIVIDUALS WITH PATELLOFEMORAL PAIN SYNDROME: A SYSTEMATIC REVIEW. Journal of Orthopaedic & Sports Physical Therapy 2012;42(6):A54 | Contra to protocol：Study on Non-acupuncture or Non-moxibustion interventions |
|  | Roos EM, Juhl C. Osteoarthritis year in review: Rehabilitation and outcomes. Osteoarthritis and Cartilage 2012;20:S8 | Contra to protocol:included studies involving patients with other diseases. Interventions are not acupuncture and moxibustion. |
|  | Holstein K, Klamroth R, Richards M, et al. Pain management in patients with haemophilia: a European survey. Haemophilia 2012;18(5):743-52 | Contra to protocol:did not focused on knee osteoarthritis |
|  | Hopton AK, Curnoe S, Kanaan M, et al. Acupuncture in practice: Mapping the providers, the patients and the settings in a national cross-sectional survey. BMJ open 2012;2 (1) (no pagination)(e000456) | Contra to protocol:did not focused on knee osteoarthritis |
|  | Cummings M. The development of group acupuncture for chronic knee pain was all about providing frequent electroacupuncture. Acupuncture in Medicine 2012;30(4):363-64 | Contra to protocol:not a systematic review |
|  | Carnes DBP, Homer KEBM, Miles CLBP, et al. Effective Delivery Styles and Content for Self-management Interventions for Chronic Musculoskeletal Pain: A Systematic Literature Review. Clinical Journal of Pain 2012;28(4):344-54 | Contra to protocol：Study on Non-acupuncture or Non-moxibustion interventions |
|  | Lambing A, Kohn-Converse B, Hanagavadi S, et al. Use of acupuncture in the management of chronic haemophilia pain. Haemophilia 2012;18(4):613-17 | Contra to protocol:did not focused on knee osteoarthritis |
|  | Gore MP, Sadosky ABP, Leslie DLP, et al. Therapy Switching, Augmentation, and Discontinuation in Patients with Osteoarthritis and Chronic Low Back Pain. Pain Practice 2012;12(6):457-68 | Contra to protocol:included studies involving patients with other diseases. Interventions are not acupuncture and moxibustion. |
|  | Giggins O, Fullen B, Coughlan G. Neuromuscular electrical stimulation in the treatment of knee osteoarthritis: a systematic review and meta-analysis. Clinical Rehabilitation 2012;26(10):867-81 | Contra to protocol：Study on Non-acupuncture or Non-moxibustion interventions |
|  | Fouladbakhsh J. Complementary and Alternative Modalities to Relieve Osteoarthritis Symptoms: A review of the evidence on several therapies often used for osteoarthritis management. Orthopaedic Nursing March/April 2012;31(2):115-21 | Contra to protocol:not a systematic review |
|  | Marchalik D, Lipsky A, Petrov D, et al. Dermatologic Presentations of Orthopedic Pathologies: A Review of Diagnosis and Treatment. American Journal of Clinical Dermatology 2012;13(5):293-310 | Contra to protocol:did not focused on knee osteoarthritis |
|  | Iversen MDSDDPTMPHBPT. Rehabilitation Interventions for Pain and Disability in Osteoarthritis. AJN, American Journal of Nursing 2012;112(3) Supplement(1):S32-S37 | Contra to protocol:not a systematic review |
|  | Macfarlane GJ, Paudyal P, Doherty M, et al. A systematic review of evidence for the effectiveness of practitioner-based complementary and alternative therapies in the management of rheumatic diseases: osteoarthritis. Rheumatology 2012;51(12):2224-33 | Contra to protocol：Study on Non-acupuncture or Non-moxibustion interventions |
|  | Ragle RL, Sawitzke AD. Nutraceuticals in the Management of Osteoarthritis: A Critical Review. Drugs & Aging 2012;29(9):717-31 | Contra to protocol:not a systematic review |
|  | Musial F, Choi K-E, Gabriel T, et al. The effect of electroacupuncture and tramadol on experimental tourniquet pain. Acupuncture in Medicine 2012;30(1):21-26 | Contra to protocol:did not focused on knee osteoarthritis |
|  | Fouladbakhsh JPA-BCAHNBCC. Complementary and Alternative Modalities to Relieve Osteoarthritis Symptoms. AJN, American Journal of Nursing 2012;112(3) Supplement(1):S44-S51 | Contra to protocol:not a systematic review |
|  | Martin MS, Van Sell S, Danter J. Glucosamine and Chondroitin: An Appropriate Adjunct Treatment of Symptomatic Osteoarthritis of the Knee. Orthopaedic Nursing May/June 2012;31(3):160-66 | Contra to protocol：Study on Non-acupuncture or Non-moxibustion interventions |
|  | Pinto D, Robertson MC, Hansen P, et al. Cost-effectiveness of nonpharmacologic, nonsurgical interventions for hip and/or knee osteoarthritis: systematic review. Value in Health 2012;15(1):1-12 | Contra to protocol:included studies involving patients with other diseases. Interventions are not acupuncture and moxibustion. |
|  | Petrou S. Rationale and methodology for trial-based economic evaluation. Clinical Investigation 2012;2(12):1191-200 | Contra to protocol: Irrelevant to the gist of the article. |
|  | Robbins L, Kulesa MG. The State of the Science in the Prevention and Management of Osteoarthritis: Experts Recommend Ways to Increase Nurses' Awareness and Knowledge of Osteoarthritis. Orthopaedic Nursing March/April 2012;31(2):74-81 | Contra to protocol:not a systematic review |
|  | Bodnar RJ. Endogenous opiates and behavior: 2011. Peptides 2012;38(2):463-522 | Contra to protocol: Irrelevant to the gist of the article. |
|  | Abstracts and Highlight Papers of the 31st Annual European Society of Regional Anaesthesia (ESRA) Congress 2012. Regional Anesthesia & Pain Medicine September/October 2012;37(5):E1-E311 | Contra to protocol: Irrelevant to the gist of the article. |
|  | Peng PWHMF. Tai Chi and Chronic Pain. Regional Anesthesia & Pain Medicine July/August 2012;37(4):372-82 | Contra to protocol：Study on Non-acupuncture or Non-moxibustion interventions |
|  | Sophie M, Ford B. Management of Pain in Parkinson's Disease. CNS Drugs 2012;26(11):937-48 | Contra to protocol:did not focused on knee osteoarthritis |
|  | DrugScan series. Journal of Pharmacy Practice & Research 2012;42(3):235-45 | Contra to protocol: Irrelevant to the gist of the article. |
|  | Winters-Stone KMP, Schwartz ALFNPP, Hayes SCP, et al. A prospective model of care for breast cancer rehabilitation: Bone health and arthralgias. Cancer 2012;118 Supplement(8):2288-99 | Contra to protocol:did not focused on knee osteoarthritis |
|  | Kirkendall DT, Garrett WE. Management of the Retired Athlete with Osteoarthritis of the Knee. Cartilage 2012;3(1_suppl):69S-76S | Contra to protocol:not a systematic review |
|  | Abst D-FreeCommPosters. Medicine & Science in Sports & Exercise 2012;44(5S) Supplement(2):267-958 | Contra to protocol: Irrelevant to the gist of the article. |
|  | Mithoefer K, Peterson L, Saris D, et al. Management of the Retired Athlete with Osteoarthritis of the Knee. Cartilage 2012;3(1_suppl) Supplement(1):69S-76S | Contra to protocol:not a systematic review |
|  | Pongparadee C, Penserga E, Lee DJ-S, et al. Current considerations for the management of musculoskeletal pain in Asian countries: a special focus on cyclooxygenase-2 inhibitors and non-steroid anti-inflammation drugs. International Journal of Rheumatic Diseases 2012;15(4):341-47 | Contra to protocol：Study on Non-acupuncture or Non-moxibustion interventions |
|  | Latimer NR, Bhanu AC, Whitehurst DGT. Inconsistencies in NICE guidance for acupuncture: reanalysis and discussion. Acupuncture in Medicine 2012;30(3):182-86 | Contra to protocol: Irrelevant to the gist of the article. |
|  | Yachoui R, Kolasinski SL. Complementary and alternative medicine for rheumatic diseases. Aging Health 2012;8(4):403-12 | Contra to protocol:did not focused on knee osteoarthritis |
|  | Park JP, Hughes AKP. Nonpharmacological Approaches to the Management of Chronic Pain in Community-Dwelling Older Adults: A Review of Empirical Evidence. Journal of the American Geriatrics Society 2012;60(3):555-68 | Contra to protocol:not a systematic review |
|  | Abst A-FreeCommSlides. Medicine & Science in Sports & Exercise 2012;44(5S) Supplement(2):84-117 | Contra to protocol: Irrelevant to the gist of the article. |
|  | Smith BH, Torrance N, Johnson M. Assessment and management of neuropathic pain in primary care. Pain Management 2012;2(6):553-59 | Contra to protocol: Irrelevant to the gist of the article. |
|  | Purepong N, Jitvimonrat A, Sitthipornvorakul E, et al. External validity in randomised controlled trials of acupuncture for osteoarthritis knee pain. Acupuncture in Medicine 2012;30(3):187-94 | Contra to protocol:not a systematic review |
|  | Woolacott NF, Corbett MS, Rice SJC. The use and reporting of WOMAC in the assessment of the benefit of physical therapies for the pain of osteoarthritis of the knee: findings from a systematic review of clinical trials. Rheumatology 2012;51(8):1440-46 | Contra to protocol：Study on Non-acupuncture or Non-moxibustion interventions |
|  | 5-Minute Clinical Consult 2012, The, 2012. | Contra to protocol: Irrelevant to the gist of the article. |
|  | da Costa BR, Rutjes AWS, Johnston BC, et al. Methods to convert continuous outcomes into odds ratios of treatment response and numbers needed to treat: meta-epidemiological study. International Journal of Epidemiology 2012;41(5):1445-59 | Contra to protocol: Irrelevant to the gist of the article. |
|  | Robbins LDSW, Kulesa MGBSRNONCC. The State of the Science in the Prevention and Management of Osteoarthritis. AJN, American Journal of Nursing 2012;112(3) Supplement(1):S3-S11 | Contra to protocol:not a systematic review |
|  | Soni A, Joshi A, Mudge N, et al. Supervised exercise plus acupuncture for moderate to severe knee osteoarthritis: a small randomised controlled trial. Acupuncture in Medicine 2012;30(3):176-81 | Contra to protocol:not a systematic review |
|  | Program. Medicine & Science in Sports & Exercise 2012;44(5S) Supplement(2):1-83 | Contra to protocol: Irrelevant to the gist of the article. |
|  | Urruela MA, Suarez-Almazor ME. Acupuncture in the treatment of rheumatic diseases. Current Rheumatology Reports 2012;14(6):589-97 | Contra to protocol:not a systematic review |
|  | . Wenham CM, Conaghan PPFF. Concise guide to the diagnosis and management of osteoarthritis. Prescriber 2012;23(1-2):16-28 | Contra to protocol:not a systematic review |
|  | Day MAMA, Thorn BEPA, Burns JWP. The Continuing Evolution of Biopsychosocial Interventions for Chronic Pain. Journal of Cognitive Psychotherapy 2012;26(2):114-29 | Contra to protocol: Irrelevant to the gist of the article. |
|  | Street RL, Elwyn G, Epstein RM. Patient preferences and healthcare outcomes: an ecological perspective. Expert Review of Pharmacoeconomics & Outcomes Research 2012;12(2):167-80 | Contra to protocol: Irrelevant to the gist of the article. |
|  | Fortney LMD, Abraham NRMDF. Managing Noncancer-related Chronic Pain Without Opioids. Primary Care Reports 2012;18(11):137-50 | Contra to protocol: Irrelevant to the gist of the article. |
|  | Coeytaux RRMDP, Park JJDKMPL. Acupuncture Research in the Era of Comparative Effectiveness Research. Annals of Internal Medicine 2013;158(4):287-88 | Contra to protocol:not a systematic review |
|  | Malfait A-M, Schnitzer TJ. Towards a mechanism-based approach to pain management in osteoarthritis. Nature Reviews Rheumatology 2013;9(11):654-64 | Contra to protocol:not a systematic review |
|  | Cameron M, Chrubasik S. Topical herbal therapies for treating osteoarthritis. Cochrane Database of Systematic Reviews 2013; (5). http://onlinelibrary.wiley.com/doi/10.1002/14651858.CD010538/abstract. | Contra to protocol：Study on Non-acupuncture or Non-moxibustion interventions |
|  | Davis AM, MacKay C. Osteoarthritis year in review: Outcome of rehabilitation. Osteoarthritis and Cartilage 2013;21(10):1414-24 | Contra to protocol: included studies involving patients with other chronic diseases and not a systematic review. |
|  | Niravath P. Aromatase inhibitor-induced arthralgia: a review. Annals of Oncology 2013;24(6):1443-49 | Contra to protocol:not a systematic review |
|  | Shelton LRDNPRNFNPBCCNE. A closer look at osteoarthritis. Nurse Practitioner 2013;38(7):30-36 | Contra to protocol:not a systematic review |
|  | Recent Literature. Focus on Alternative & Complementary Therapies an Evidence-Based Approach 2013;18(4):e23-e30 | Contra to protocol: Irrelevant to the gist of the article. |
|  | Borczuk PMD, Burns BDDOF, Henry GLMDF. An Evidence-Based Approach To The Evaluation And Treatment Of Low Back Pain In The Emergency Department. Emergency Medicine Practice 2013;15(7):1-23 | Contra to protocol:did not focused on knee osteoarthritis |
|  | Stemberger R, Kerschan-Schindl K. Osteoarthritis: physical medicine and rehabilitation--nonpharmacological management. Wiener Medizinische Wochenschrift 2013;163(9-10):228-35 | Contra to protocol:not a systematic review |
|  | Colquhoun DP, Novella SPMD. Acupuncture Is Theatrical Placebo. Anesthesia & Analgesia 2013;116(6):1360-63 | Contra to protocol:not a systematic review |
|  | Saramago P, Weatherly H, Manca A, et al. Comparative effectiveness of active versus sham acupuncture versus usual care in the management of chronic, non-cancer pain in primary care. Value in Health 2013;16 (7):A556-A57 | Contra to protocol:not a systematic review |
|  | Wluka AE, Lombard CB, Cicuttini FM. Tackling obesity in knee osteoarthritis. Nature Reviews Rheumatology 2013;9(4):225-35 | Contra to protocol: included studies involving patients with other chronic diseases and not a systematic review. |
|  | Singh Jasvinder A, Dohm M, Borkhoff C. Total joint replacement surgery versus conservative care for knee osteoarthritis and other non-traumatic diseases. Cochrane Database of Systematic Reviews 2013; (9). http://onlinelibrary.wiley.com/doi/10.1002/14651858.CD010732/abstract. | Contra to protocol：included studies of protocol |
|  | Lu D-FPRNa, Hart LKPRNa, Lutgendorf SKPb, et al. The effect of healing touch on the pain and mobility of persons with osteoarthritis: A feasibility study. Geriatric Nursing 2013;34(4):314-22 | Contra to protocol:not a systematic review |
|  | Borenstein D. Mechanical low back pain-a rheumatologist's view. Nature Reviews Rheumatology 2013;9(11):643-53 | Contra to protocol:did not focused on knee osteoarthritis |
|  | Abdulla A, Adams N, Bone M, et al. Guidance on the management of pain in older people. Age & Ageing 2013;42 Suppl 1:i1-57 | Contra to protocol:not a systematic review |
|  | Abdulla A, Bone M, Adams N, et al. Evidence-based clinical practice guidelines on management of pain in older people. Age & Ageing 2013;42(2):151-53 | Contra to protocol: Irrelevant to the gist of the article. |
|  | de Oliveira Melo M, Aragao FA, Vaz MA. Neuromuscular electrical stimulation for muscle strengthening in elderly with knee osteoarthritis - a systematic review. Complementary Therapies in Clinical Practice 2013;19(1):27-31 | Contra to protocol：Study on Non-acupuncture or Non-moxibustion interventions |
|  | Crosby SSMDP. Primary Care Management of Non-English-Speaking Refugees Who Have Experienced Trauma: A Clinical Review. JAMA 2013;310(5):519-28 | Contra to protocol: Irrelevant to the gist of the article. |
|  | Chen LXMDP, Mao JJMDM, Fernandes S, et al. Integrating Acupuncture With Exercise-Based Physical Therapy for Knee Osteoarthritis: A Randomized Controlled Trial. JCR: Journal of Clinical Rheumatology 2013;19(6):308-16 | Contra to protocol:not a systematic review |
|  | Holistic Nursing: A Handbook for Practice, 2013. |  |
|  | Dean BJF, Gwilym SE, Carr AJ. Why does my shoulder hurt? A review of the neuroanatomical and biochemical basis of shoulder pain. British Journal of Sports Medicine 2013;47(17):1095&hyhen;104 | Contra to protocol:did not focused on knee osteoarthritis |
|  | Thiberville S-Dab, Moyen Nab, Dupuis-Maguiraga Lcd, et al. Chikungunya fever: Epidemiology, clinical syndrome, pathogenesis and therapy. Antiviral Research 2013;99(3):345-70 | Contra to protocol: Irrelevant to the gist of the article. |
|  | Vas J, Aguilar I. Towards an ideal treatment of the degenerative osteoarthritis of knee with acupuncture: Review of the different approaches, technologies, and his effects. [Spanish]  Hacia un tratamiento optimo de la artrosis de rodilla con acupuntura: revision de los diferentes enfoques, tecnicas, y sus efectos. Revista Internacional de Acupuntura 2013;7(4):131-43 | Contra to protocol:not a systematic review |
|  | 5-Minute Clinical Consult 2013, The, 2013. |  |
|  | Glickman-Simon RMD, Alper BSMDMF. Acupuncture for PTSD, Naturopathy for Cardiovascular Risk; Yoga for Osteoarthritis; Chasteberry for PMS; and Antioxidants for Cardiovascular Events. Explore: The Journal of Science & Healing 2013;9(6):385-88 | Contra to protocol:did not focused on knee osteoarthritis |
|  | Bishop FL, Salmon C. Advertising, expectations and informed consent: the contents and functions of acupuncture leaflets. Acupuncture in Medicine 2013;31(4):351-57 | Contra to protocol: Irrelevant to the gist of the article. |
|  | Gaught AM, Carneiro KA. Evidence for determining the exercise prescription in patients with osteoarthritis. Physician & Sportsmedicine 2013;41(1):58-65 | Contra to protocol:not a systematic review |
|  | Vickers AJD, for the Acupuncture Trialists C. Placing Acupuncture in Perspective-Reply. JAMA Internal Medicine 2013;173(8):714 | Contra to protocol: Irrelevant to the gist of the article. |
|  | Uthman OAapiarsr, van der Windt DApopce, Jordan JLrim, et al. Exercise for lower limb osteoarthritis: systematic review incorporating trial sequential analysis and network meta-analysis. BMJ September 2013;21(347) | Contra to protocol:included studies involving patients with other diseases. Interventions are not acupuncture and moxibustion. |
|  | Perez and Brady's Principles and Practice of Radiation Oncology, 2013. | Contra to protocol: Irrelevant to the gist of the article. |
|  | Vickers AJ, Maschino AC, Lewith G, et al. Responses to the Acupuncture Trialists' Collaboration individual patient data meta-analysis. Acupuncture in Medicine 2013;31(1):98-100 | Contra to protocol:not a systematic review |
|  | Koog YH, Gil M, We SR, et al. Barriers to Participant Retention in Knee Osteoarthritis Clinical Trials: A Systematic Review. Seminars in Arthritis and Rheumatism 2013;42(4):346-54 | Contra to protocol：Study on Non-acupuncture or Non-moxibustion interventions |
|  | Spruit MA, Singh SJ, Garvey C, et al. An Official American Thoracic Society/European Respiratory Society Statement: Key Concepts and Advances in Pulmonary Rehabilitation. American Journal of Respiratory & Critical Care Medicine 2013;188(8):e13-e64 | Contra to protocol: Irrelevant to the gist of the article. |
|  | Southern Regional Program Abstracts. Journal of Investigative Medicine 2013;61(2):373-524 | Contra to protocol: Irrelevant to the gist of the article. |
|  | Anonymous. Abstracts - Society for Acupuncture Research 2013 International Conference: Impact of Acupuncture Research on 21st Century Healthcare. Journal of Alternative and Complementary Medicine Conference: Society for Acupuncture Research 2013;19(7) | Contra to protocol:not a systematic review |
|  | Wale JLP, Belizan MM, Nadel JMAJD, et al. The Cochrane Library review titles that are important to users of health care, a Cochrane Consumer Network project. Health Expectations 2013;16(4):e146-e63 | Contra to protocol: Irrelevant to the gist of the article. |
|  | Manara M, Bortoluzzi A, Favero M, et al. Italian society for Rheumatology recommendations for the management of hand osteoarthritis. Reumatismo 2013;65(4):167-85 | Contra to protocol:did not focused on knee osteoarthritis |
|  | Briggs JPMD, Killen JMD. Perspectives on Complementary and Alternative Medicine Research. JAMA 2013;310(7):691-92 | Contra to protocol: Irrelevant to the gist of the article. |
|  | Arya RK, Jain V. Osteoarthritis of the knee joint: An overview. Journal, Indian Academy of Clinical Medicine 2013;14(2):154-62 | Contra to protocol:not a systematic review |
|  | Guidance on the management of pain in older people. Age & Ageing 2013;42(suppl_1) Supplement(1):i1-i57 | Contra to protocol: Irrelevant to the gist of the article. |
|  | Park J, Manotas K, Hooyman N. Chronic pain management by ethnically and racially diverse older adults: pharmacological and nonpharmacological pain therapies. Pain Management 2013;3(6):435-54 | Contra to protocol:did not focused on knee osteoarthritis |
|  | Shengelia R, Parker SJ, Ballin M, et al. Complementary therapies for osteoarthritis: are they effective? Pain Management Nursing 2013;14(4):e274-88 | Contra to protocol:not a systematic review |
|  | Brim RL, Miller FG. The potential benefit of the placebo effect in sham-controlled trials: implications for risk-benefit assessments and informed consent. Journal of Medical Ethics 2013;39(11):703-07 | Contra to protocol: Irrelevant to the gist of the article. |
|  | Newshan GPNPRNCS, Staats JAMSANP. Evidence-Based Pain Guidelines in HIV Care. Journal of the Association of Nurses in AIDS Care January/February 2013;24(1):S112-S26 | Contra to protocol: Irrelevant to the gist of the article. |
|  | Li S, Yu B, Zhou D, et al. Electromagnetic fields for treating osteoarthritis. Cochrane Database of Systematic Reviews 2013; (12). http://onlinelibrary.wiley.com/doi/10.1002/14651858.CD003523.pub2/abstract. | Contra to protocol：Study on Non-acupuncture or Non-moxibustion interventions |
|  | Jevsevar DSMDMBA. Treatment of Osteoarthritis of the Knee: Evidence-Based Guideline, 2nd Edition. Journal of the American Academy of Orthopaedic Surgeons 2013;21(9):571-76 | Contra to protocol:not a systematic review |
|  | Burnstock G. Purinergic mechanisms and pain-An update. European Journal of Pharmacology 2013;716(1-2-3):24-40 | Contra to protocol: Irrelevant to the gist of the article. |
|  | Johnson CDCM, World Federation of C. Abstracts of the Scientific Sessions from the WFC'S 12th Biennial Congress Proceedings, Durban, South Africa, April 10-13, 2013. Journal of Chiropractic Medicine 2013;12(2):92-142 | Contra to protocol: Irrelevant to the gist of the article. |
|  | Da Costa BR, Nuesch E, Rutjes AW, et al. Combining follow-up and change data is valid in meta-analyses of continuous outcomes: A meta-epidemiological study. Journal of Clinical Epidemiology 2013;66(8):847-55 | Contra to protocol:included studies involving patients with other diseases. Interventions are not acupuncture and moxibustion. |
|  | Prady SL, Burch J, Crouch S, et al. Controlling practitioner-patient relationships in acupuncture trials: a systematic review and meta-regression. Acupuncture in Medicine 2013;31(2):162-71 | Contra to protocol:did not focused on knee osteoarthritis |
|  | Abstracts of Scientific Papers and Posters Presented at the Annual Meeting of the Association of Academic Physiatrists: New Orleans, Louisiana March 6 - 10, 2013. American Journal of Physical Medicine & Rehabilitation 2013;92(3) Supplement(1):a1-a80 | Contra to protocol: Irrelevant to the gist of the article. |
|  | Bhatia D, Bejarano T, Novo M. Current interventions in the management of knee osteoarthritis. Journal of Pharmacy and Bioallied Sciences 2013;5(1):30-38 | Contra to protocol:not a systematic review |
|  | Claydon LSP, Chesterton LSP, Barlas PD, et al. Alternating-Frequency TENS Effects on Experimental Pain in Healthy Human Participants: A Randomized Placebo-Controlled Trial. Clinical Journal of Pain 2013;29(6):533-39 | Contra to protocol: Irrelevant to the gist of the article. |
|  | Negm A, Lorbergs A, Macintyre NJ. Efficacy of low frequency pulsed subsensory threshold electrical stimulation vs placebo on pain and physical function in people with knee osteoarthritis: systematic review with meta-analysis. Osteoarthritis & Cartilage 2013;21(9):1281-9 | Contra to protocol：Study on Non-acupuncture or Non-moxibustion interventions |
|  | Wang S-MMD, Harris REP, Lin Y-CMDMPH, et al. Acupuncture in 21st Century Anesthesia: Is There a Needle in the Haystack? Anesthesia & Analgesia 2013;116(6):1356-59 | Contra to protocol: Irrelevant to the gist of the article. |
|  | Lingaraju R, Ashburn MA. Pain management in the elderly. Aging Health 2013;9(3):265-74 | Contra to protocol: Irrelevant to the gist of the article. |
|  | Shengelia RMD, Parker SJAB, Ballin MGNPBCCDE, et al. Complementary Therapies for Osteoarthritis: Are They Effective? Pain Management Nursing 2013;14(4):e274-e88 | Contra to protocol:not a systematic review |
|  | Qin Y, He J, Xia L, et al. Effects of electro-acupuncture on oestrogen levels, body weight, articular cartilage histology and MMP-13 expression in ovariectomised rabbits. Acupuncture in Medicine 2013;31(2):214-21 | Contra to protocol:did not focused on knee osteoarthritis |
|  | Abstracts and Highlight Papers of the 32nd Annual European Society of Regional Anaesthesia & Pain Therapy (ESRA) Congress 2013: Invited Speaker Highlight Papers. Regional Anesthesia & Pain Medicine September/October 2013;38(5):E1-E259 | Contra to protocol: Irrelevant to the gist of the article. |
|  | Orozco L, Munar A, Soler R, et al. Treatment of Knee Osteoarthritis With Autologous Mesenchymal Stem Cells: A Pilot Study. Transplantation 2013;95(12):1535-41 | Contra to protocol：Study on Non-acupuncture or Non-moxibustion interventions |
|  | Yang C-PMD, Chang M-HMD, Li T-CMDP, et al. Predicting Prognostic Factors in a Randomized Controlled Trial of Acupuncture Versus Topiramate Treatment in Patients With Chronic Migraine. Clinical Journal of Pain 2013;29(11):982-87 | Contra to protocol:did not focused on knee osteoarthritis |
|  | White A, Meinen M, Sheikh A. Summaries of recent papers. Acupuncture in Medicine 2013;31(4):443-49 | Contra to protocol: Irrelevant to the gist of the article. |
|  | Jubb J, Bensing JM. The sweetest pill to swallow: How patient neurobiology can be harnessed to maximise placebo effects. Neuroscience & Biobehavioral Reviews 2013;37(10Part2):2709-20 | Contra to protocol: Irrelevant to the gist of the article. |
|  | Jevsevar DSMDMBA, Brown GAMDP, Jones DLPTP, et al. The American Academy of Orthopaedic Surgeons Evidence-Based Guideline on: Treatment of Osteoarthritis of the Knee, 2nd Edition. Journal of Bone & Joint Surgery - American Volume 2013;95(20):1885-86 | Contra to protocol:not a systematic review |
|  | 184. 185. 186. 187.  188. Abstracts and Highlight Papers of the 33rd Annual European Society of Regional Anaesthesia & Pain Therapy (ESRA) Congress 2014. Regional Anesthesia & Pain Medicine September/October 2014;39(5):e1-E324 | Contra to protocol: Irrelevant to the gist of the article. |
|  | McCartney CJL, Nelligan K. Postoperative Pain Management After Total Knee Arthroplasty in Elderly Patients: Treatment Options. Drugs & Aging 2014;31(2):83-91 | Contra to protocol：Study on Non-acupuncture or Non-moxibustion interventions |
|  | Abstracts of Scientific Papers and Posters Presented at the Annual Meeting of the Association of Academic Physiatrists. American Journal of Physical Medicine & Rehabilitation 2014;93(3) Supplement(2):a1-a97 | Contra to protocol: Irrelevant to the gist of the article. |
|  | Kmietowicz Z. Acupuncture does not improve chronic knee pain, study finds. BMJ October 2014;4(349) | Contra to protocol:not a systematic review |
|  | Nelson AE, Allen KD, Golightly YM, et al. A systematic review of recommendations and guidelines for the management of osteoarthritis: The chronic osteoarthritis management initiative of the U.S. bone and joint initiative. Seminars in Arthritis & Rheumatism 2014;43(6):701-12 | Contra to protocol：Study on Non-acupuncture or Non-moxibustion interventions |
|  | Fishbain DAMDF, Cole BP, Lewis JEP, et al. Does Pain Interfere with Antidepressant Depression Treatment Response and Remission in Patients with Depression and Pain? An Evidence-Based Structured Review. Pain Medicine 2014;15(9):1522-39 | Contra to protocol:did not focused on knee osteoarthritis |
|  | McAlindon TE, Bannuru RR, Sullivan MC, et al. OARSI guidelines for the non-surgical management of knee osteoarthritis. Osteoarthritis & Cartilage 2014;22(3):363-88 | Contra to protocol：Study on Non-acupuncture or Non-moxibustion interventions |
|  | Primary Care Medicine: Office Evaluation and Management of the Adult Patient, 2014. | Contra to protocol: Irrelevant to the gist of the article. |
|  | 5 Minute Clinical Consult Standard 2015, The, 2014. | Contra to protocol: Irrelevant to the gist of the article. |
|  | Kiefer DMD. Needle (or Laser) the Knee for Relief from Chronic Osteoarthritis Pain? Integrative Medicine Alert 2014;17(11):121-23 | Contra to protocol:not a systematic review |
|  | Uhl RLMD, Roberts TTMD, Papaliodis DNMD, et al. Management of Chronic Musculoskeletal Pain. Journal of the American Academy of Orthopaedic Surgeons 2014;22(2):101-10 | Contra to protocol：Study on Non-acupuncture or Non-moxibustion interventions |
|  | ASAM Principles of Addiction Medicine, The, 2014. | Contra to protocol: Irrelevant to the gist of the article. |
|  | Bratton's Family Medicine Board Review, 2014. | Contra to protocol: Irrelevant to the gist of the article. |
|  | Fransen M, McConnell S, Hernandez-Molina G, et al. Exercise for osteoarthritis of the hip. Cochrane Database of Systematic Reviews 2014; (4). http://onlinelibrary.wiley.com/doi/10.1002/14651858.CD007912.pub2/abstract. | Contra to protocol:did not focused on knee osteoarthritis |
|  | Glass' Office Gynecology, 2014. | Contra to protocol: Irrelevant to the gist of the article. |
|  | Pettit JMD, Glickman-Simon RMD. Osteopathy and Low Back Pain, Acupuncture and in Vitro Fertilization, Tai Chi and Osteoarthritis, Andrographis Paniculata and Ulcerative Colitis, and Acupressure and Vertigo. Explore: The Journal of Science & Healing 2014;10(1):62-66 | Contra to protocol: included studies involving patients with other chronic diseases and not a systematic review. |
|  | Kroon Féline PB, van der Burg Lennart RA, Buchbinder R, et al. Self-management education programmes for osteoarthritis. Cochrane Database of Systematic Reviews 2014; (1). http://onlinelibrary.wiley.com/doi/10.1002/14651858.CD008963.pub2/abstract. | Contra to protocol:not a systematic review |
|  | M J. Transcutaneous electrical nerve stimulation: review of effectiveness. Nursing Standard 2014;28(40):44-53 | Contra to protocol：Study on Non-acupuncture or Non-moxibustion interventions |
|  | Lippincott Manual of Nursing Practice, 2014. | Contra to protocol: Irrelevant to the gist of the article. |
|  | Maly M. Osteoarthritis year in review: Rehabilitation and outcomes. Osteoarthritis and Cartilage 2014;22:S5-S6 | Contra to protocol:included studies involving patients with other diseases. Interventions are not acupuncture and moxibustion. |
|  | Barrie J, Loughlin D. Managing chronic pain in adults. Nursing Standard 2014;29(7):50-58 | Contra to protocol: Irrelevant to the gist of the article. |
|  | Vance CGT, Dailey DL, Rakel BA, et al. Using TENS for pain control: the state of the evidence. Pain Management 2014;4(3):197-209 | Contra to protocol：Study on Non-acupuncture or Non-moxibustion interventions |
|  | Martins F, Kaster TMD, Schutzler LM, et al. Factors Influencing Further Acupuncture Usage and a More Positive Outcome in Patients With Osteoarthritis of the Knee and the Hip: A 3-Year Follow-up of a Randomized Pragmatic Trial. Clinical Journal of Pain 2014;30(11):953-59 | Contra to protocol:not a systematic review |
|  | Li H, Zeng C, Lei GH. Comment on 'Neuromuscular electrical stimulation in the treatment of knee osteoarthritis: a systematic review and meta-analysis'. Clinical Rehabilitation 2014;28(11):1145-6 | Contra to protocol：Study on Non-acupuncture or Non-moxibustion interventions |
|  | Bhardwaj A, Nagandla K. Musculoskeletal symptoms and orthopaedic complications in pregnancy: pathophysiology, diagnostic approaches and modern management. Postgraduate Medical Journal 2014;90(1066):450-60 | Contra to protocol: Irrelevant to the gist of the article. |
|  | Demcoe ARMD, Bohm ERMMD. A 67-year-old woman with knee pain. CMAJ Canadian Medical Association Journal 2014;186(17):1311-14 | Contra to protocol:not a systematic review |
|  | Williams N, Lawler D, Dilworth P. It's all in the mind! Or is it? Positive acupuncture responses in patients with dementia: a series of case reports. Acupuncture in Medicine 2014;32(2):186-89 | Contra to protocol:did not focused on knee osteoarthritis |
|  | La Porta C, Bura SA, Negrete R, et al. Involvement of the endocannabinoid system in osteoarthritis pain. European Journal of Neuroscience 2014;39(3):485-500 | Contra to protocol：Study on Non-acupuncture or Non-moxibustion interventions |
|  | Bodnar RJ. Endogenous opiates and behavior: 2013. Peptides 2014;62 Supplement(C):67-136 | Contra to protocol: Irrelevant to the gist of the article. |
|  | Hinman RSP, McCrory PP, Pirotta MP, et al. Acupuncture for Chronic Knee Pain: A Randomized Clinical Trial. JAMA 2014;312(13):1313-22 | Contra to protocol:not a systematic review |
|  | Fry LMDVMa, Neary SMDVMb, Sharrock JDVMc, et al. Acupuncture for Analgesia in Veterinary Medicine. Topics in Companion Animal Medicine 2014;29(2):35-42 | Contra to protocol: Irrelevant to the gist of the article. |
|  | Zhang RPD, Lao LPD, Ren KPD, et al. Mechanisms of Acupuncture-Electroacupuncture on Persistent Pain. Anesthesiology 2014;120(2):482-503 | Contra to protocol:did not focused on knee osteoarthritis |
|  | Ashford SPFNPCNE, Williard JMSNA. Osteoarthritis: A review. Nurse Practitioner 2014;39(5):1-8 | Contra to protocol:not a systematic review |
|  | McGlothlin AEP, Lewis RJMDP. Minimal Clinically Important Difference: Defining What Really Matters to Patients. JAMA 2014;312(13):1342-43 | Contra to protocol: Irrelevant to the gist of the article. |
|  | Crawford CBA, Lee CMA, May TDO, et al. Physically Oriented Therapies for the Self-Management of Chronic Pain Symptoms. Pain Medicine 2014;15 Supplement(1):S54-S65 | Contra to protocol：Study on Non-acupuncture or Non-moxibustion interventions |
|  | Sierpina VSMD. The Harvard Medical School Guide to Tai Chi. Explore: The Journal of Science & Healing May/June 2014;10(3):196-97 | Contra to protocol: Irrelevant to the gist of the article. |
|  | Bruyere O, Cooper C, Pelletier JP, et al. An algorithm recommendation for the management of knee osteoarthritis in Europe and internationally: A report from a task force of the European Society for Clinical and Economic Aspects of Osteoporosis and Osteoarthritis (ESCEO). Seminars in Arthritis and Rheumatism 2014;44(3):253-63 | Contra to protocol:not a systematic review |
|  | Barton C, Balachandar V, Lack S, et al. Patellar taping for patellofemoral pain: a systematic review and meta-analysis to evaluate clinical outcomes and biomechanical mechanisms. British Journal of Sports Medicine 2014;48(6):417-24 | Contra to protocol：Study on Non-acupuncture or Non-moxibustion interventions |
|  | White A, Meinen M. Summaries of recent papers. Acupuncture in Medicine 2014;32(6):525-30 | Contra to protocol: Irrelevant to the gist of the article. |
|  | Zoorob RMDMPHF, Chakrabarty SMDMF, O'Hara HMDM, et al. Which CAM modalities are worth considering? Journal of Family Practice 2014;63(10):585-90 | Contra to protocol: Irrelevant to the gist of the article. |
|  | Zeng C, Gao SG, Lei GH. Comment on corbett etal. entitled "Acupuncture and other physical treatments for the relief of pain due to osteoarthritis of the knee: Network meta-analysis". Osteoarthritis and Cartilage 2014;22(5):710-11 | Contra to protocol:not a systematic review |
|  | Glickman-Simon RMD, Karp JMD, Sethi TMD. Ginkgo for Alzheimer's Disease, Tai Chi for Parkinson's Disease Revisited, Acupuncture for Postoperative Vomiting, Cranberry for Urinary Tract Infection, Curcuma domestica for knee osteoarthritis. Explore: The Journal of Science & Healing 2015;11(4):326-30 | Contra to protocol:included studies involving patients with other diseases. Interventions are not acupuncture and moxibustion. |
|  | Southern Regional Meeting Abstracts. Journal of Investigative Medicine 2015;63(2):324-482 | Contra to protocol: Irrelevant to the gist of the article. |
|  | Fransen M, McConnell S, Harmer Alison R, et al. Exercise for osteoarthritis of the knee. Cochrane Database of Systematic Reviews 2015; (1). http://onlinelibrary.wiley.com/doi/10.1002/14651858.CD004376.pub3/abstract. | Contra to protocol:included studies involving patients with other diseases. Interventions are not acupuncture and moxibustion. |
|  | Baxter GDTD, Dphil, Mba, et al. Treating Chronic Knee Pain With Acupuncture. JAMA 2015;313(6):626-27 | Contra to protocol:not a systematic review |
|  | Bennell K, Hinman R. Rehabilitation and outcomes. Osteoarthritis and Cartilage 2015;23:A25 | Contra to protocol: included studies involving patients with other chronic diseases and not a systematic review. |
|  | Pietrosimone B, Blackburn JT, Harkey MS, et al. Clinical strategies for addressing muscle weakness following knee injury. Clinics in Sports Medicine 2015;34(2):285-300 | Contra to protocol:not a systematic review |
|  | Humphries TJ, Kessler CM. Managing chronic pain in adults with haemophilia: current status and call to action. Haemophilia 2015;21(1):41-51 | Contra to protocol:did not focused on knee osteoarthritis |
|  | Pedersen BK, Saltin B. Exercise as medicine - evidence for prescribing exercise as therapy in 26 different chronic diseases. Scandinavian Journal of Medicine & Science in Sports 2015;25 Supplement(3):1-72 | Contra to protocol:not a systematic review |
|  | Glickman-Simon R, Wallace J. Acupuncture for knee osteoarthritis, chasteberry for premenstrual syndrome, probiotics for irritable bowel syndrome, yoga for hypertension, and trigger point dry needling for plantar fasciitis. Explore: The Journal of Science and Healing 2015;11(2):157-61 | Contra to protocol: included studies involving patients with other chronic diseases and not a systematic review. |
|  | Abstracts and Highlight Papers of the 34th Annual European Society of Regional Anaesthesia & Pain Therapy (ESRA) Congress 2015. Regional Anesthesia & Pain Medicine September/October 2015;40(5):e1-e208 | Contra to protocol: Irrelevant to the gist of the article. |
|  | Paley CA, Johnson MI. Investigation into the effects of using two or four acupuncture needles with bidirectional rotation on experimentally-induced contact heat pain in healthy subjects. Acupuncture in Medicine 2015;33(1):23-29 | Contra to protocol:not a systematic review |
|  | McGeeney BEMDMPH. Acupuncture Is All Placebo and Here Is Why. Headache 2015;55(3):465-69 | Contra to protocol:did not focused on knee osteoarthritis |
|  | Abstracts of Scientific Papers and Posters Presented at the Annual Meeting of the Association of Academic Physiatrists. American Journal of Physical Medicine & Rehabilitation March 2015;94(3):1 | Contra to protocol: Irrelevant to the gist of the article. |
|  | Sharma L. Clinical. Osteoarthritis and Cartilage 2015;23:A24 | Contra to protocol:not a systematic review |
|  | Taylor's Manual of Family Medicine, 2015. | Contra to protocol: Irrelevant to the gist of the article. |
|  | Hooten MWMDa, Cohen SPMDbc. Evaluation and Treatment of Low Back Pain: A Clinically Focused Review for Primary Care Specialists. Mayo Clinic Proceedings 2015;90(12):1699-718 | Contra to protocol:did not focused on knee osteoarthritis |
|  | Washington Manual of Outpatient Internal Medicine, The, 2015. | Contra to protocol: Irrelevant to the gist of the article. |
|  | Anonymous. 21st Annual International Integrative Medicine Conference. Advances in Integrative Medicine Conference: 21st Annual International Integrative Medicine Conference Australia 2015;2(2) | Contra to protocol:not a systematic review |
|  | DeVita, Hellman, and Rosenberg's Cancer: Principles & Practice of Oncology, 2015. | Contra to protocol: Irrelevant to the gist of the article. |
|  | Wong Lit Wan D, Wang Y, Xue CCL, et al. Local and distant acupuncture points stimulation for chronic musculoskeletal pain: A systematic review on the comparative effects. European Journal of Pain 2015;19(9):1232-47 | Contra to protocol:did not focused on knee osteoarthritis |
|  | A. Ennin KMDa, Coyner KJMDb. Treatment options other than total knee arthroplasty in young patients with knee osteoarthritis: Part I. Current Orthopaedic Practice May/June 2015;26(3):228-35 | Contra to protocol:not a systematic review |
|  | Alling FAMD. The Healing Effects of Belief in Medical Practices and Spirituality. Explore: The Journal of Science & Healing 2015;11(4):273-80 | Contra to protocol: Irrelevant to the gist of the article. |
|  | Barton CJ, Lack S, Hemmings S, et al. The 'Best Practice Guide to Conservative Management of Patellofemoral Pain': incorporating level 1 evidence with expert clinical reasoning. British Journal of Sports Medicine 2015;49(14):923-34 | Contra to protocol: Irrelevant to the gist of the article. |
|  | McNeill S, Fullen BM. Acupuncture for osteoarthritis of the knee: Common points and treatment parameters used. Physiotherapy (United Kingdom) 2015;101:eS968-eS69 | Contra to protocol:not a systematic review |
|  | Danelich IM, Wright SS, Lose JM, et al. Safety of Nonsteroidal Antiinflammatory Drugs in Patients with Cardiovascular Disease. Pharmacotherapy:The Journal of Human Pharmacology & Drug Therapy 2015;35(5):520-35 | Contra to protocol:did not focused on knee osteoarthritis |
|  | Bennell KL, Buchbinder R, Hinman RS. Physical therapies in the management of osteoarthritis: current state of the evidence. Current Opinion in Rheumatology 2015;27(3):304-11 | Contra to protocol:not a systematic review |
|  | Carr D. In this issue. Acupuncture in Medicine 2015;33(4):253 | Contra to protocol: Irrelevant to the gist of the article. |
|  | Lee S, Kim KH. Acupuncture for knee pain: is there no more room for further research? Acupuncture in Medicine 2015;33(6):499 | Contra to protocol:not a systematic review |
|  | Christiansen BA, Bhatti S, Goudarzi R, et al. Management of Osteoarthritis with Avocado/Soybean Unsaponifiables. Cartilage 2015;6(1):30-44 | Contra to protocol: Irrelevant to the gist of the article. |
|  | Zeng C, Li H, Yang T, et al. Electrical stimulation for pain relief in knee osteoarthritis: systematic review and network meta-analysis. Osteoarthritis & Cartilage 2015;23(2):189-202 | Contra to protocol：Study on Non-acupuncture or Non-moxibustion interventions |
|  | Crossley KM, Callaghan MJ, van Linschoten R. Patellofemoral pain. BMJ November 2015;7(351) | Contra to protocol: Irrelevant to the gist of the article. |
|  | Chen R, Chen M, Su T, et al. Heat-sensitive moxibustion in patients with osteoarthritis of the knee: a three-armed multicentre randomised active control trial. Acupuncture in Medicine 2015;33(4):262-69 | Contra to protocol:not a systematic review |
|  | D'Arcy YMSRNCCNS. Managing pain in obese patients: Meeting weighty challenges. Nursing 2015;45(2):42-49 | Contra to protocol:did not focused on knee osteoarthritis |
|  | Hanlon JTPMS, Semla TPMSP, Schmader KEMD. Alternative Medications for Medications in the Use of High-Risk Medications in the Elderly and Potentially Harmful Drug-Disease Interactions in the Elderly Quality Measures. Journal of the American Geriatrics Society 2015;63(12):e8-e18 | Contra to protocol: Irrelevant to the gist of the article. |
|  | Sasek CMPAC. An update on primary care management of knee osteoarthritis. Journal of the American Academy of Physician Assistants 2015;28(1):37-43 | Contra to protocol：Study on Non-acupuncture or Non-moxibustion interventions |
|  | Hou PW, Fu PK, Hsu HC, et al. Traditional Chinese medicine in patients with osteoarthritis of the knee. [Review]. Journal of Traditional & Complementary Medicine 2015;5(4):182-96 | Contra to protocol:not a systematic review |
|  | Palmer S, Cramp F, Lewis R, et al. Diagnosis, Management and Assessment of Adults with Joint Hypermobility Syndrome: A UK-Wide Survey of Physiotherapy Practice. Musculoskeletal Care 2015;13(2):101-11 | Contra to protocol: Irrelevant to the gist of the article. |
|  | Chang WJ, Bennell KL, Hodges PW, et al. Combined exercise and transcranial direct current stimulation intervention for knee osteoarthritis: protocol for a pilot randomised controlled trial. BMJ open 2015;5(8):e008482 | Contra to protocol：Study on Non-acupuncture or Non-moxibustion interventions |
|  | Hinman RSP, Pirotta MP, Bennell KLP. Treating Chronic Knee Pain With Acupuncture-Reply. JAMA 2015;313(6):628-29 | Contra to protocol:not a systematic review |
|  | Ropper AHMD, Zafonte RDDO. Sciatica. New England Journal of Medicine 2015;372(13):1240-48 | Contra to protocol: Irrelevant to the gist of the article. |
|  | Dommerholt J, Grieve R, Hooks T, et al. A critical overview of the current myofascial pain literature - October 2015. Journal of Bodywork and Movement Therapies 2015;19(4):736-46 | Contra to protocol:not a systematic review |
|  | Mallen CNrpigp, Hay Epocr. Managing back pain and osteoarthritis without paracetamol. BMJ April 2015;4(350) | Contra to protocol：Study on Non-acupuncture or Non-moxibustion interventions |
|  | Briani RVab, de Oliveira Silva Db, Pazzinatto MFb, et al. Comparison of frequency and time domain electromyography parameters in women with patellofemoral pain. Clinical Biomechanics 2015;30(3):302-07 | Contra to protocol:did not focused on knee osteoarthritis |
|  | Schulenburg JBSNRNNMDCLT. Considerations for Complementary and Alternative Interventions for Pain. AORN Journal 2015;101(3):319-26 | Contra to protocol: Irrelevant to the gist of the article. |
|  | Buchbinder Rdpoce, Harris IApoos, Sprowson Aapot, et al. Management of degenerative meniscal tears and the role of surgery. BMJ June 2015;6(350) | Contra to protocol：Study on Non-acupuncture or Non-moxibustion interventions |
|  | Halabchi FMD, Mazaheri RMD, Mansournia MAMDMPH, et al. Additional Effects of an Individualized Risk Factor-Based Approach on Pain and the Function of Patients With Patellofemoral Pain Syndrome: A Randomized Controlled Trial. Clinical Journal of Sport Medicine 2015;25(6):478-86 | Contra to protocol:not a systematic review |
|  | Glickman-Simon RMD, Mukherji AMDMPH. Moxibustion for Asthma, Acupuncture for Epilepsy, Psychological Therapies for Irritable Bowel Syndrome, Exercise Training for Multiple Sclerosis, and Comfrey Root for Acute Back Pain. Explore: The Journal of Science & Healing 2015;11(1):67-71 | Contra to protocol:did not focused on knee osteoarthritis |
|  | Callaghan MJ. Year in review: Rehabilitation and outcomes. Osteoarthritis and Cartilage 2016;24:S6 | Contra to protocol: included studies involving patients with other chronic diseases and not a systematic review. |
|  | Selvendran S, Aggarwal N, Vassiliou VMAMDMF, et al. Pirouetting Away the Pain With Music. JCR: Journal of Clinical Rheumatology 2015;21(5):263-66 | Contra to protocol: Irrelevant to the gist of the article. |
|  | Dwyer LMa, Parkin-Smith GFMMMDb, Brantingham JWDCPc, et al. Manual and Manipulative Therapy in Addition to Rehabilitation for Osteoarthritis of the Knee: Assessor-Blind Randomized Pilot Trial. Journal of Manipulative & Physiological Therapeutics 2015;38(1):1-21e2 | Contra to protocol:not a systematic review |
|  | Bervoets DC, Luijsterburg PA, Alessie JJ, et al. Massage therapy has short-term benefits for people with common musculoskeletal disorders compared to no treatment: a systematic review. Journal of Physiotherapy 2015;61(3):106-16 | Contra to protocol：Study on Non-acupuncture or Non-moxibustion interventions |
|  | Witteveen Angelique GH, Hofstad Cheriel J, Kerkhoffs Gino MMJ. Hyaluronic acid and other conservative treatment options for osteoarthritis of the ankle. Cochrane Database of Systematic Reviews 2015; (10). http://onlinelibrary.wiley.com/doi/10.1002/14651858.CD010643.pub2/abstract. | Contra to protocol:did not focused on knee osteoarthritis |
|  | White A, Cummings M. Acupuncture for knee osteoarthritis: study by Hinman et al represents missed opportunities. Acupuncture in Medicine 2015;33(1):84-86 | Contra to protocol:not a systematic review |
|  | Weiner DKMD, Fang MMD, Gentili AMD, et al. Deconstructing Chronic Low Back Pain in the Older Adult-Step by Step Evidence and Expert-Based Recommendations for Evaluation and Treatment: Part I: Hip Osteoarthritis. Pain Medicine 2015;16(5):886-97 | Contra to protocol: Irrelevant to the gist of the article. |
|  | Bannuru RRMDP, McAlindon TEMD, Sullivan MCBA, et al. Effectiveness and Implications of Alternative Placebo Treatments: A Systematic Review and Network Meta-analysis of Osteoarthritis Trials. Annals of Internal Medicine 2015;163(5):365-72 | Contra to protocol：Study on Non-acupuncture or Non-moxibustion interventions |
|  | Fleckenstein JMD, Banzer WMD, PhD. Treating Chronic Knee Pain With Acupuncture. JAMA 2015;313(6):627 | Contra to protocol:not a systematic review |
|  | Glickman-Simon RMD, Pettit JMD. Viscum album (mistletoe) for Pancreatic Cancer, Electromagnetic Field Therapy for Osteoarthritis, Homeopathy for Multidrug-Resistant Tuberculosis, Vitamin D for Depression, Acupuncture for Insomnia. Explore: The Journal of Science & Healing 2015;11(3):231-35 | Contra to protocol:did not focused on knee osteoarthritis |
|  | Edwards JJ, Khanna M, Jordan KP, et al. Quality indicators for the primary care of osteoarthritis: a systematic review. Annals of the Rheumatic Diseases 2015;74(3):490-98 | Contra to protocol：Study on Non-acupuncture or Non-moxibustion interventions |
|  | White A, Langweiler M, Meinen M. Summaries of recent papers. Acupuncture in Medicine 2015;33(3):242-47 | Contra to protocol: Irrelevant to the gist of the article. |
|  | Hinman RS, Forbes A, Williamson E, et al. Acupuncture for chronic knee pain: a randomised clinical trial. Authors' reply. Acupuncture in Medicine 2015;33(1):86-88 | Contra to protocol:not a systematic review |
|  | Narouze SMDP, Souzdalnitski DMDP. Obesity and Chronic Pain: Systematic Review of Prevalence and Implications for Pain Practice. Regional Anesthesia & Pain Medicine March/April 2015;40(2):91-111 | Contra to protocol:did not focused on knee osteoarthritis |
|  | Abstracts. Journal of Gastroenterology & Hepatology 2016;31 Asia Pacific Digestive Week (APDW) Innovative Approaches to Gastroenterology, Kobe, Japan, 2-5 November(2016S3):7-441 | Contra to protocol: Irrelevant to the gist of the article. |
|  | Pan R-YMDP, Hsu Y-CMD, Wong C-SMDP, et al. Comparing complementary alternative treatment for chronic shoulder pain of myofascial origin: Collateral meridian therapy versus local tender area-related meridians therapy. Medicine 2016;95(35):e4634 | Contra to protocol:not a systematic review |
|  | Abstracts and Highlight Papers of the 35th Annual European Society of Regional Anaesthesia & Pain Therapy (ESRA) Congress 2016. Regional Anesthesia & Pain Medicine September/October 2016;41(5):e1-e162 | Contra to protocol: Irrelevant to the gist of the article. |
|  | Fritz JMPTP, Rundell SDPTDPTP, Dougherty PDC, et al. Deconstructing Chronic Low Back Pain in the Older Adult-Step by Step Evidence and Expert-Based Recommendations for Evaluation and Treatment. Part VI: Lumbar Spinal Stenosis. Pain Medicine 2016;17(3):501-10 | Contra to protocol:did not focused on knee osteoarthritis |
|  | Poster Session. Movement Disorders 2016;31 Abstracts of the Twentieth International Congress of Parkinson's Disease and Movement(DisordersS2):S1-S697 | Contra to protocol: Irrelevant to the gist of the article. |
|  | Alsehimy M, Al-Dharrab A, Jamal B. The use of dextrose prolotherapy for myofascial pain dysfunction syndrome: a double-blind placebo-controlled study. Egyptian Journal of Oral & Maxillofacial Surgery 2016;7(3):75-80 | Contra to protocol:not a systematic review |
|  | Tang Y, Yin H-Y, Rubini P, et al. Acupuncture-Induced Analgesia: A Neurobiological Basis in Purinergic Signaling. Neuroscientist 2016;22(6):563-78 | Contra to protocol:did not focused on knee osteoarthritis |
|  | Duan-Porter WMDP, Goldstein KMMDM, McDuffie JRPMPH, et al. Reporting of Sex Effects by Systematic Reviews on Interventions for Depression, Diabetes, and Chronic Pain. Annals of Internal Medicine 2016;165(3):184-93 | Contra to protocol:included studies involving patients with other diseases. Interventions are not acupuncture and moxibustion. |
|  | Rodriguez-Merchan EC. Conservative treatment of acute knee osteoarthritis: A review of the Cochrane Library. Journal of Acute Disease 2016;5(3):190-93 | Contra to protocol:not a systematic review |
|  | Abstracts of Scientific Papers and Posters Presented at the Annual Meeting of the Association of Academic Physiatrists. American Journal of Physical Medicine & Rehabilitation 2016;95(3) Supplement(1):a1-a122 | Contra to protocol: Irrelevant to the gist of the article. |
|  | Bell AEMD, Falconi ADO. Acupuncture for the Treatment of Sports Injuries in an Austere Environment. Current Sports Medicine Reports March/April 2016;15(2):111-15 | Contra to protocol:not a systematic review |
|  | Lurie J, Tomkins-Lane C. Management of lumbar spinal stenosis. BMJ January 2016;9(352) | Contra to protocol:did not focused on knee osteoarthritis |
|  | Western Regional Meeting 2016, Carmel, California, January 28-30, 2016. Journal of Investigative Medicine 2016;64(1):135-232 | Contra to protocol: Irrelevant to the gist of the article. |
|  | Bell AE, Falconi A. Acupuncture for the Treatment of Sports Injuries in an Austere Environment. Current Sports Medicine Reports 2016;15(2):111-5 | Contra to protocol:did not focused on knee osteoarthritis |
|  | Chen LX, Zhou ZR, Li YL, et al. Transcutaneous Electrical Nerve Stimulation in Patients With Knee Osteoarthritis: Evidence From Randomized-controlled Trials. Clinical Journal of Pain 2016;32(2):146-54 | Contra to protocol：Study on Non-acupuncture or Non-moxibustion interventions |
|  | Bennell KL, Hall M, Hinman RS. Osteoarthritis year in review 2015: rehabilitation and outcomes. Osteoarthritis & Cartilage 2016;24(1):58-70 | Contra to protocol:not a systematic review |
|  | Western Regional Meeting Program At-A-Glance Carmel, California January 28-30, 2016. Journal of Investigative Medicine 2016;64(1):69-134 | Contra to protocol: Irrelevant to the gist of the article. |
|  | Mallen C, Hay E. Managing back pain and osteoarthritis without paracetamol: Physical treatments are the way forward. British Journal of Sports Medicine 2016;50(20):1286-87 | Contra to protocol:not a systematic review |
|  | Abbasi J. As Opioid Epidemic Rages, Complementary Health Approaches to Pain Gain Traction. JAMA 2016;316(22):2343-44 | Contra to protocol：Study on Non-acupuncture or Non-moxibustion interventions |
|  | Mechanisms and Management of Pain for the Physical Therapist, 2016. | Contra to protocol: Irrelevant to the gist of the article. |
|  | Zhou K, Fan AY, Wang T. Acupuncture for Chronic Knee Pain: A Critical Appraisal of an Australian Randomized Controlled Trial. Medical Acupuncture 2016;28(1):40-45 | Contra to protocol:not a systematic review |
|  | Volpato HB, Szego P, Lenza M, et al. Femoral quadriceps neuromuscular electrical stimulation after total knee arthroplasty: a systematic review. Einstein 2016;14(1):77-98 | Contra to protocol:did not focused on knee osteoarthritis |
|  | . Abbott JH, Mehta P, Winser S. Economic evaluations of physical therapy interventions for hip or knee osteoarthritis: A systematic review. Osteoarthritis and Cartilage 2016;24:S495 | Contra to protocol:included studies involving patients with other diseases. Interventions are not acupuncture and moxibustion. |
|  | Cherian JJ, Jauregui JJ, Leichliter AK, et al. The effects of various physical non-operative modalities on the pain in osteoarthritis of the knee. Bone & Joint Journal 2016;98-B(1 Suppl A):89-94 | Contra to protocol：Study on Non-acupuncture or Non-moxibustion interventions |
|  | Buchbinder R, Harris IA, Sprowson A. Management of degenerative meniscal tears and the role of surgery. British Journal of Sports Medicine 2016;50(22):1413-16 | Contra to protocol:not a systematic review |
|  | Polsunas PJBS, Sowa GMDP, Fritz JMPTP, et al. Deconstructing Chronic Low Back Pain in the Older Adult-Step by Step Evidence and Expert-Based Recommendations for Evaluation and Treatment: Part X: Sacroiliac Joint Syndrome. Pain Medicine 2016;17(9):1638-47 | Contra to protocol:did not focused on knee osteoarthritis |
|  | Schinhan MMD, Neubauer B, Pieber KMD, et al. Climbing Has a Positive Impact on Low Back Pain: A Prospective Randomized Controlled Trial. Clinical Journal of Sport Medicine 2016;26(3):199-205 | Contra to protocol:not a systematic review |
|  | Chughtai M, Elmallah RD, Mistry JB, et al. Nonpharmacologic Pain Management and Muscle Strengthening following Total Knee Arthroplasty. The Journal of Knee Surgery 2016;29(3):194-200 | Contra to protocol：Study on Non-acupuncture or Non-moxibustion interventions |
|  | Yao & Artusio's Anesthesiology: Problem-Oriented Patient Management, 2016. | Contra to protocol: Irrelevant to the gist of the article. |
|  | Lee G-CMDa. What's New in Adult Reconstructive Knee Surgery. Journal of Bone & Joint Surgery - American Volume 2016;98(2):156-65 | Contra to protocol:not a systematic review |
|  | Aresti Nsrit, orthopaedic s, National Medical Director's clinical f, et al. Hip osteoarthritis. BMJ July 2016;9(354) | Contra to protocol:did not focused on knee osteoarthritis |
|  | Bodnar RJ. Endogenous opiates and behavior: 2014. Peptides 2016;75 Supplement(C):18-70 | Contra to protocol: Irrelevant to the gist of the article. |
|  | Gabler CM, Lepley AS, Uhl TL, et al. Comparison of Transcutaneous Electrical Nerve Stimulation and Cryotherapy for Increasing Quadriceps Activation in Patients With Knee Pathologies. Journal of Sport Rehabilitation 2016;25(3):294-300 | Contra to protocol：Study on Non-acupuncture or Non-moxibustion interventions |
|  | Chen L-XMD, Zhou Z-RMD, Li Y-LMD, et al. Transcutaneous Electrical Nerve Stimulation in Patients With Knee Osteoarthritis: Evidence From Randomized-controlled Trials. Clinical Journal of Pain 2016;32(2):146-54 | Contra to protocol:not a systematic review |
|  | Crossley KM, Callaghan MJ, Linschoten Rv. Patellofemoral pain. British Journal of Sports Medicine 2016;50(4):247-50 | Contra to protocol: Irrelevant to the gist of the article. |
|  | Juel JMD, Liguori SMD, Liguori AMD, et al. A New Method for Sham-Controlled Acupuncture in Experimental Visceral Pain - a Randomized, Single-Blinded Study. Pain Practice 2016;16(6):669-79 | Contra to protocol:did not focused on knee osteoarthritis |
|  | Lauche RP, Graf N, Cramer HP, et al. Efficacy of Cabbage Leaf Wraps in the Treatment of Symptomatic Osteoarthritis of the Knee: A Randomized Controlled Trial. Clinical Journal of Pain 2016;32(11):961-71 | Contra to protocol:not a systematic review |
|  | Cox JD, Varatharajan SBM, Cote PDCP, et al. Effectiveness of Acupuncture Therapies to Manage Musculoskeletal Disorders of the Extremities: A Systematic Review. Journal of Orthopaedic & Sports Physical Therapy 2016;46(6):409-29 | included studies involving patients with other diseases. Cohort study and case control trial articles were included. |
|  | Crossley KM, van Middelkoop M, Callaghan MJ, et al. 2016 Patellofemoral pain consensus statement from the 4th International Patellofemoral Pain Research Retreat, Manchester. Part 2: recommended physical interventions (exercise, taping, bracing, foot orthoses and combined interventions). British Journal of Sports Medicine 2016;50(14):844-52 | Contra to protocol: Irrelevant to the gist of the article. |
|  | Sha T, Gao LL, Zhang CH, et al. An update on acupuncture point injection. Qjm 2016;109(10):639-41 | Contra to protocol:not a systematic review |
|  | Ai JW, Li DS, Liu Y, et al. Effectiveness of traditional Chinese medicine therapy for knee osteoarthritis: A network meta-analysis. [Chinese]. Chinese Journal of Evidence-Based Medicine 2016;16(5):532-42 | Contra to protocol: Original from Chinese database |
|  | Salehi AMDMPHP, Marzban MMP, Imanieh MH. The Evaluation of Curative Effect of Acupuncture: A Review of Systematic and Meta-Analysis Studies. Journal of Evidence-Based Complementary & Alternative Medicine 2016;21(3):202-14 | Contra to protocol:did not focused on knee osteoarthritis |
|  | Cooke MRNP, Walker RRNP, Aitken LMRNP, et al. Pre-operative self-efficacy education vs. usual care for patients undergoing joint replacement surgery: a pilot randomised controlled trial. Scandinavian Journal of Caring Sciences 2016;30(1):74-82 | Contra to protocol:not a systematic review |
|  | Mistiaen P, van Osch M, van Vliet L, et al. The effect of patient-practitioner communication on pain: a systematic review. European Journal of Pain 2016;20(5):675-88 | Contra to protocol: Irrelevant to the gist of the article. |
|  | Ai JW, Liu Y, Li DS, et al. Efficacy of traditional Chinese medicine therapy and hyaluronic acid for knee osteoarthritis: A network Meta-analysis. [Chinese]. Chinese Journal of Tissue Engineering Research 2016;20(20):3000-11 | Contra to protocol: Original from Chinese database |
|  | Sharma L. Osteoarthritis year in review 2015: clinical. Osteoarthritis & Cartilage 2016;24(1):36-48 | Contra to protocol:not a systematic review |
|  | Goldgrub RM, Cote PDCP, Sutton DMM, et al. The Effectiveness of Multimodal Care for the Management of Soft Tissue Injuries of the Shoulder: A Systematic Review by the Ontario Protocol for Traffic Injury Management (OPTIMa) Collaboration. Journal of Manipulative & Physiological Therapeutics 2016;39(2):121-39e1 | Contra to protocol:did not focused on knee osteoarthritis |
|  | Liao Y, Li X, Li N, et al. Electroacupuncture protects against articular cartilage erosion by inhibiting mitogen-activated protein kinases in a rat model of osteoarthritis. Acupuncture in Medicine 2016;34(4):290-95 | Contra to protocol:not a systematic review |
|  | Nahin RLPMPH, Boineau RMDMA, Khalsa PSDCP, et al. Evidence-Based Evaluation of Complementary Health Approaches for Pain Management in the United States. Mayo Clinic Proceedings 2016;91(9):1292-306 | Contra to protocol: Irrelevant to the gist of the article. |
|  | Edwards RR, Dworkin RH, Turk DC, et al. Patient phenotyping in clinical trials of chronic pain treatments: IMMPACT recommendations. Pain 2016;157(9):1851-71 | Contra to protocol:not a systematic review |
|  | Walach H. The Efficacy Paradox and Its Consequences for Research in Psychotherapy (and Elsewhere). Psychology of Consciousness: Theory, Research, and Practice 2016;3(2):154-61 | Contra to protocol: Irrelevant to the gist of the article. |
|  | Sutton DABMM, Nordin MD, Cote PDCP, et al. The Effectiveness of Multimodal Care for Soft Tissue Injuries of the Lower Extremity: A Systematic Review by the Ontario Protocol for Traffic Injury Management (OPTIMa) Collaboration. Journal of Manipulative & Physiological Therapeutics 2016;39(2):95-109e2 | Contra to protocol:did not focused on knee osteoarthritis |
|  | Grissa MHMD, Baccouche HMD, Boubaker HMD, et al. Acupuncture vs intravenous morphine in the management of acute pain in the ED[white star],[white star][white star],[black star],[black star][black star]. American Journal of Emergency Medicine 2016;34(11):2112-16 | Contra to protocol:not a systematic review |
|  | White A, Meinen M. Summaries of recent papers. Acupuncture in Medicine 2016;34(2):152-54 | Contra to protocol: Irrelevant to the gist of the article. |
|  | Gooberman-Hill R, Beswick A, Wylde V, et al. Interventions for the management of long-term post-surgical pain after total knee replacement: A systematic review of randomised controlled trials. Osteoporosis International 2016;1):S306-S07 | Contra to protocol:did not focused on knee osteoarthritis |
|  | Liu C, Su Y, He J, et al. Using data mining approaches to select acupoints in acupuncture and moxibustion for knee osteoarthritis. African Journal of Traditional, Complementary and Alternative Medicines 2016;13(2):120-32 | Contra to protocol:not a systematic review |
|  | Chen Lmd, associate p, Michalsen Amd, et al. Management of chronic pain using complementary and integrative medicine: State of the Art Review. BMJ April 2017;29(357) | Contra to protocol: included studies involving patients with other chronic diseases and not a systematic review. |
|  | 59th Annual Scientific Meeting American Headache Society(R) June 8 - 11, 2017 Westin Boston Waterfront Boston, MA. Headache 2017;57 S3, Program(Abstracts):The 59th Annual American Headache Society Meeting:113-226 | Contra to protocol: Irrelevant to the gist of the article. |
|  | Yang GSMSNRN, Kim HJPRN, Griffith KAPMPHC, et al. Interventions for the Treatment of Aromatase Inhibitor-Associated Arthralgia in Breast Cancer Survivors: A Systematic Review and Meta-analysis. [Article]: Cancer Nursing July/August 2017;40(4):E26-E41, 2017 | Contra to protocol:did not focused on knee osteoarthritis |
|  | Abstracts. Movement Disorders 2017;32 Abstracts of the 21st International Congress of Parkinson's Disease and Movement(DisordersS2):S1-S627 | Contra to protocol: Irrelevant to the gist of the article. |
|  | Wu JM, Zhou JM, Liu CM, et al. A Prospective Study Comparing Platelet-Rich Plasma and Local Anesthetic (LA)/Corticosteroid in Intra-Articular Injection for the Treatment of Lumbar Facet Joint Syndrome. [Article]: Pain Practice September 2017;17(7):914-924, 2017. | Contra to protocol:did not focused on knee osteoarthritis |
|  | Yang ZMDa, Zhao LPa, Xie XPb, et al. The effectiveness of acupuncture for chronic pain with depression: A systematic review protocol. [Article]: Medicine November 2017;96(47):e8800, 2017. | Contra to protocol：included studies of protocol |
|  | International Neuromodulation Society's 13th World Congress Neuromodulation: Technology Changing Lives Edinburgh, Scotland, United Kingdom May 27-June 1, 2017. [Miscellaneous]: Neuromodulation October 2017;20(7):e336-e783, 2017. | Contra to protocol: Irrelevant to the gist of the article. |
|  | Gupta A, Huettner DP, Dukewich M. Comparative Effectiveness Review of Cooled Versus Pulsed Radiofrequency Ablation for the Treatment of Knee Osteoarthritis: A Systematic Review. Pain Physician 2017;20(3):155-71 | Contra to protocol：Study on Non-acupuncture or Non-moxibustion interventions |
|  | Till SR, Wahl HN, As-Sanie S. The role of nonpharmacologic therapies in management of chronic pelvic pain: what to do when surgery fails. [Miscellaneous Article]: Current Opinion in Obstetrics & Gynecology August 2017;29(4):231-239, 2017. | Contra to protocol:did not focused on knee osteoarthritis |
|  | Abstracts From 2017 NANS 20th Annual Meeting, Las Vegas, NV, USA January 19-22, 2017. [Miscellaneous]: Neuromodulation October 2017;20(7):e122-e335, 2017. | Contra to protocol: Irrelevant to the gist of the article. |
|  | Woods B, Manca A, Weatherly H, et al. Cost-effectiveness of adjunct non-pharmacological interventions for osteoarthritis of the knee. PLoS ONE [Electronic Resource] 2017;12(3):e0172749 | Contra to protocol：Study on Non-acupuncture or Non-moxibustion interventions |
|  | Marcus DMMD. Noninvasive Treatments for Acute, Subacute, and Chronic Low Back Pain. [Letter]: Annals of Internal Medicine December 5, 2017;167(11):832, 2017. | Contra to protocol:did not focused on knee osteoarthritis |
|  | Ali ANDMPHMHS, Rosenberger LNDMS, Weiss TRMPH, et al. Massage Therapy and Quality of Life in Osteoarthritis of the Knee: A Qualitative Study. Pain Medicine 2017;18(6):1168-75 | Contra to protocol:not a systematic review |
|  | Abstracts and Highlight Papers of the 36th Annual European Society of Regional Anaesthesia & Pain Therapy (ESRA) Congress 2017. [Abstract]: Regional Anesthesia & Pain Medicine September/October 2017;42(5S) Supplement 1:e1-e200, 2017. | Contra to protocol: Irrelevant to the gist of the article. |
|  | Zhu LL, Zhou JY, Luo L, et al. Comparison of the efficacy between conventional moxibustion and smoke-free moxibustion on knee osteoarthritis: study protocol of a randomized controlled trial. Trials [Electronic Resource] 2017;18(1):24 | Contra to protocol：included studies of protocol |
|  | Yeung W-F, Chung K-F, Yu Y-MB, et al. What predicts a positive response to acupuncture? A secondary analysis of three randomised controlled trials of insomnia. Acupuncture in Medicine 2017;35(1):24-29 | Contra to protocol:did not focused on knee osteoarthritis |
|  | AAPM 2017 Annual Meeting Abstracts. Pain Medicine 2017;18(3):569-621 | Contra to protocol:not a systematic review |
|  | Paper Session. [Abstract]: Journal of the American Geriatrics Society September 2017;65 S2, Abstracts from the Sixth Chinese Congress on Gerontology and Health Industry:S295-S369, 2017. | Contra to protocol: Irrelevant to the gist of the article. |
|  | Xu Q, Chen B, Wang Y, et al. The effectiveness of manual therapy for relieving pain, stiffness, and dysfunction in knee osteoarthritis: A systematic review and meta-analysis. Pain Physician 2017;20(4):229-43 | Contra to protocol：Study on Non-acupuncture or Non-moxibustion interventions |
|  | Zachary SSMD, Danesh HMD. Acupuncture in the Management of Chronic Pain. Topics in Pain Management 2017;32(10):1-9 | Contra to protocol:not a systematic review |
|  | Tedesco DMD, Gori DMD, Desai KRP, et al. Drug-Free Interventions to Reduce Pain or Opioid Consumption After Total Knee Arthroplasty: A Systematic Review and Meta-analysis. [Article]: JAMA Surgery October 2017;152(10):e172872-, 2017. | Contra to protocol:included studies involving patients with other diseases. Interventions are not acupuncture and moxibustion. |
|  | Crowley RBSJ, Kirschner NP, Dunn ASMD, et al. Health and Public Policy to Facilitate Effective Prevention and Treatment of Substance Use Disorders Involving Illicit and Prescription Drugs: An American College of Physicians Position Paper. Annals of Internal Medicine 2017;166(10):733-36 | Contra to protocol: Irrelevant to the gist of the article. |
|  | Lin Y-CMDMPH, Wan LBS, Jamison RNP. Using Integrative Medicine in Pain Management: An Evaluation of Current Evidence. [Review]: Anesthesia & Analgesia December 2017;125(6):2081-2093, 2017. | Contra to protocol:not a systematic review |
|  | Walsh D. Guidelines and recommendations for managing challenging chronic pain conditionsI83. The National Institute for Health and Care Excellence Revised Guidelines for the Management of Non-Specific Low Back Pain and Sciatica. Rheumatology 2017;56(suppl_2) Supplement(2):ii18-ii25 | Contra to protocol: Irrelevant to the gist of the article. |
|  | Yeh CHRNP, Lin W-CMDMS, Suen LK-PRNMPHP, et al. Auricular Point Acupressure to Manage Aromatase Inhibitor-Induced Arthralgia in Postmenopausal Breast Cancer Survivors: A Pilot Study. Oncology Nursing Forum 2017;44(4):476-87 | Contra to protocol：Study on Non-acupuncture or Non-moxibustion interventions |
|  | Liu J, Ma S, Mu J, et al. Integration of white matter network is associated with interindividual differences in psychologically mediated placebo response in migraine patients. [Article]: Human Brain Mapping October 2017;38(10):5250-5259, 2017. | Contra to protocol:did not focused on knee osteoarthritis |
|  | MacPherson H, Vickers A, Bland M, et al. Acupuncture for chronic pain and depression in primary care: a programme of research: NIHR Journals Library. Programme Grants for Applied Research, 2017 01, 2017 | Contra to protocol: Irrelevant to the gist of the article. |
|  | Birch S, Lee MS, Robinson N, et al. The U.K. NICE 2014 Guidelines for Osteoarthritis of the Knee: Lessons Learned in a Narrative Review Addressing Inadvertent Limitations and Bias. Journal of Alternative & Complementary Medicine 2017;23(4):242-46 | Contra to protocol:not a systematic review |
|  | Abstract. [Abstract]: Pharmacoepidemiology & Drug Safety August 2017;26 S2, Abstracts of the 33rd International Conference on Pharmacoepidemiology & Therapeutic Risk Management, Palais des congres de Montreal, Montreal, Canada, August 26-30, 2017:3-636, 2017. | Contra to protocol: Irrelevant to the gist of the article. |
|  | Masala S, Marsico S. Intra-articular injections. CardioVascular and Interventional Radiology 2017;40 (2 Supplement 1):S84-S88 | Contra to protocol:not a systematic review |
|  | Chan DKCM, Johnson MIP, Sun KOMFFF, et al. Electrical Acustimulation of the Wrist for Chronic Neck Pain: A Randomized, Sham-controlled Trial Using a Wrist-Ankle Acustimulation Device. [Article]: Clinical Journal of Pain May 2009;25(4):320-326, 2009. | Contra to protocol:did not focused on knee osteoarthritis |
|  | N. SE. Eastern Nursing Research Society: 29th Annual Scientific Sessions Abstracts. Nursing Research March/April 2017;66(2):E1-E135 | Contra to protocol: Irrelevant to the gist of the article. |
|  | Cibrian K. Nondrug Interventions Reduce Pain and Opioid Use After Total Knee Arthroplasty. The American journal of nursing 2017;117(11):62 | Contra to protocol:not a systematic review |
|  | 5-Minute Clinical Consult 2017, 2017. | Contra to protocol: Irrelevant to the gist of the article. |
|  | Yin Cab, Buchheit TEbcd, Park JJb. Acupuncture for chronic pain: an update and critical overview. [Miscellaneous Article]: Current Opinion in Anaesthesiology October 2017;30(5):583-592, 2017. | Contra to protocol:not a systematic review |
|  | Tavee JOMD, Levin KHMDF. Low Back Pain. CONTINUUM: Lifelong Learning in Neurology 2017;23(2, Selected Topics in Outpatient Neurology):467-86 | Contra to protocol: Irrelevant to the gist of the article. |
|  | Lewis J, Sim J, Barlas P. Acupuncture and electro-acupuncture for people diagnosed with subacromial pain syndrome: A multicentre randomized trial. [Article]: European Journal of Pain July 2017;21(6):1007-1019, 2017. | Contra to protocol:did not focused on knee osteoarthritis |
|  | 5-Minute Clinical Consult Premium 2018, 2017. | Contra to protocol: Irrelevant to the gist of the article. |
|  | Ferket BSap, Feldman Zms, Zhou Jda, et al. Impact of total knee replacement practice: cost effectiveness analysis of data from the Osteoarthritis Initiative. BMJ April 2017;1(356) | Contra to protocol:not a systematic review |
|  | Guo MY, Tang YJ, He ZP, et al. Cupping therapy for Treating Knee Osteoarthritis: A protocol for systematic review and meta-analysis of randomized controlled trials. European Journal of Integrative Medicine 2017;12:131-34 | Contra to protocol:included studies involving patients with other diseases. Interventions are not acupuncture and moxibustion. |
|  | Bodnar RJ. Endogenous Opiates and Behavior: 2015. Peptides February 2017;88:126-88 | Contra to protocol: Irrelevant to the gist of the article. |
|  | Brown M, Farquhar-Smith P. Pain in cancer survivors; filling in the gaps. [Article]: BJA: British Journal of Anaesthesia October 01, 2017;119(4):723-736, 2017. | Contra to protocol: Irrelevant to the gist of the article. |
|  | Hu Bab, Bai Fb, Xiong Lb, et al. The endocannabinoid system, a novel and key participant in acupuncture's multiple beneficial effects. Neuroscience & Biobehavioral Reviews June 2017;77:340-57 | Contra to protocol:not a systematic review |
|  | Tekkatte R, Berntzen B, Erwood L, et al. BHPR research: qualitativeE01. Patient-Reported Long-Term Effects of Aprogressive Resistance Training Programme. Rheumatology 2017;56(suppl_2) Supplement(2):ii187-ii214 | Contra to protocol: Irrelevant to the gist of the article. |
|  | Cibulka MTD, Bloom NJD, Enseki KRPM, et al. Hip Pain and Mobility Deficits-Hip Osteoarthritis: Revision 2017. Journal of Orthopaedic & Sports Physical Therapy 2017;47(6):A1-A37 | Contra to protocol:did not focused on knee osteoarthritis |
|  | Bruce RD, Merlin J, Lum PJ, et al. 2017 HIVMA of IDSA Clinical Practice Guideline for the Management of Chronic Pain in Patients Living With HIV. [Article]: Clinical Infectious Diseases October 30, 2017;65(10):e1-e37, 2017. | Contra to protocol: Irrelevant to the gist of the article. |
|  | Walsh NE, Pearson J, Healey EL. Physiotherapy management of lower limb osteoarthritis. British Medical Bulletin 2017;122(1):151-61 | Contra to protocol:not a systematic review |
|  | Nagar V R SS, James C R, et al. Abstracts of Scientific Papers and Posters Presented at the Annual Meeting of the Association of Academic Physiatrists. American Journal of Physical Medicine & Rehabilitation March 2017;96(3):1 | Contra to protocol: Irrelevant to the gist of the article. |
|  | Gao C, Zhao J, Yang D. Efficacy of acupuncture in pain management of chronic diseases of bone and joint: A review of literature. International Journal of Clinical and Experimental Medicine 2017;10(6):8788-800 | Contra to protocol:not a systematic review |
|  | Sadock BJ, Sadock, Virginia A, Ruiz, Pedro. Kaplan & Sadock's Comprehensive Textbook of Psychiatry, 2017. | Contra to protocol: Irrelevant to the gist of the article. |
|  | Fernandopulle SB, Perry MP, Manlapaz DM, et al. Effect of Land-Based Generic Physical Activity Interventions on Pain, Physical Function, and Physical Performance in Hip and Knee Osteoarthritis: A Systematic Review and Meta-Analysis. [Article]: American Journal of Physical Medicine & Rehabilitation November 2017;96(11):773-792, 2017. | Contra to protocol:included studies involving patients with other diseases. Interventions are not acupuncture and moxibustion. |
|  | Lin Y-CM, MPH; Wan, Limeng BS; Jamison, Robert N. PhD. Using Integrative Medicine in Pain Management: An Evaluation of Current Evidence Anesthesia & Analgesia 2017;125(6):2081–93 | Contra to protocol: Irrelevant to the gist of the article. |
|  | Chesham RA, Shanmugam S. Does preoperative physiotherapy improve postoperative, patient-based outcomes in older adults who have undergone total knee arthroplasty? A systematic review. Physiotherapy Theory & Practice 2017;33(1):9-30 | Contra to protocol：Study on Non-acupuncture or Non-moxibustion interventions |
|  | Nunez-Cortes RP, Cruz-Montecinos CPTM, Vasquez-Rosel AP, et al. Dry Needling Combined With Physical Therapy in Patients With Chronic Postsurgical Pain Following Total Knee Arthroplasty: A Case Series. Journal of Orthopaedic & Sports Physical Therapy 2017;47(3):209-16 | Contra to protocol:not a systematic review |
|  | Hainline B, Derman W, Vernec A, et al. International Olympic Committee consensus statement on pain management in elite athletes. [Miscellaneous Article]: British Journal of Sports Medicine September 2017;51(17):1245-1258, 2017. | Contra to protocol: Irrelevant to the gist of the article. |
|  | Elbadawy MAMD. Effectiveness of Periosteal Stimulation Therapy and Home Exercise Program in the Rehabilitation of Patients With Advanced Knee Osteoarthritis. Clinical Journal of Pain 2017;33(3):254-63 | Contra to protocol：Study on Non-acupuncture or Non-moxibustion interventions |
|  | Jain NBMDMa, Schneider BJMD, Kuhn JEMD, et al. What's New in Orthopaedic Rehabilitation. [Miscellaneous Article]: Journal of Bone & Joint Surgery - American Volume November 15, 2017;99(22):1956-1963, 2017. | Contra to protocol:not a systematic review |
|  | Carr DB, Cohen RI. "Are perioperative opioids obsolete?" Proceedings of an IASP Acute Pain Special Interest Group Satellite Symposium September 25, 2016 Yokohama, Japan. [Abstract]: PAIN Reports July/August 2017;2(4):e604, 2017. | Contra to protocol: Irrelevant to the gist of the article. |
|  | Hausmann LRMP, Youk AP, Kwoh CKMD, et al. Testing a Positive Psychological Intervention for Osteoarthritis. [Miscellaneous Article]: Pain Medicine October 01, 2017;18(10):1908-1920, 2017. | Contra to protocol:not a systematic review |
|  | Dowling MPMRNTBNSRGN, McDonagh B, Meade E, et al. Arthralgia in Breast Cancer Survivors: An Integrative Review of Endocrine Therapy. Oncology Nursing Forum 2017;44(3):337-49 | Contra to protocol:did not focused on knee osteoarthritis |
|  | Coste J, Montel S. Placebo-related effects: a meta-narrative review of conceptualization, mechanisms and their relevance in rheumatology. Rheumatology 2017;56(3):334-43 | Contra to protocol: Irrelevant to the gist of the article. |
|  | Stern CMBS, Pepin MJMA, Stoler JMMD, et al. Musculoskeletal Conditions in a Pediatric Population with Ehlers-Danlos Syndrome. Journal of Pediatrics February 2017;181:261-66 | Contra to protocol:not a systematic review |
|  | Narayan N, Carlucci F, Dakin S, et al. Basic science235. The Translocator Protein as a Marker of Macrophage and Stromal Activation in Inflammatory Arthritis: An Initial Investigation. Rheumatology 2017;56(suppl_2) Supplement(2):ii144-ii86 | Contra to protocol: Irrelevant to the gist of the article. |
|  | Chen N , Wang J , Mucelli A , et al. Electro-Acupuncture is Beneficial for Knee Osteoarthritis: The Evidence from Meta-Analysis of Randomized Controlled Trials[J]. The American Journal of Chinese Medicine, 2017:1-21. | Contra to protocol: Study on Non-acupuncture or Non-moxibustion interventions |
